# Molecular Signature of Prospero Homeobox 1 (PROX1) in Follicular Thyroid Carcinoma Cells

**DOI:** 10.3390/ijms20092212

**Published:** 2019-05-05

**Authors:** Magdalena Rudzińska, Małgorzata Grzanka, Anna Stachurska, Michał Mikula, Katarzyna Paczkowska, Tomasz Stępień, Agnieszka Paziewska, Jerzy Ostrowski, Barbara Czarnocka

**Affiliations:** 1 Department of Biochemistry and Molecular Biology, Centre of Postgraduate Medical Education, 01-813 Warsaw, Poland; magdda.rudzinska@gmail.com (M.R.); mgrzanka@cmkp.edu.pl (M.G.); 2 Department of Immunohematology, Centre of Postgraduate Medical Education, 01-813 Warsaw, Poland; astachurska@cmkp.edu.pl; 3 Department of Genetics, Maria Sklodowska-Curie Memorial Cancer Center and Institute of Oncology, 02-781 Warsaw, Poland; mikula.michal@gmail.com (M.M.); paczkowska.km@gmail.com (K.P.); jostrow@warman.com.pl (J.O.); 4 Clinic of Endocrinological and General Surgery, Medical University of Lodz, 93-513 Lodz, Poland; tomsamaz@wp.pl; 5 Department of Gastroenterology, Hepatology and Clinical Oncology, Centre of Postgraduate Medical Education, 01-813 Warsaw, Poland; apaziewska@cmkp.edu.pl

**Keywords:** PROX1, follicular thyroid carcinoma, RNA-seq, migration, invasion, cytoskeleton

## Abstract

The prospero homeobox 1 (PROX1) transcription factor is a product of one of the lymphangiogenesis master genes. It has also been suggested to play a role in carcinogenesis, although its precise role in tumour development and metastasis remains unclear. The aim of this study was to gain more knowledge on the PROX1 function in thyroid tumorigenesis. Follicular thyroid cancer-derived cells—CGTH-W-1—were transfected with PROX1-siRNA (small interfering RNA) and their proliferation, cell cycle, apoptosis and motility were then analysed. The transcriptional signature of *PROX1* depletion was determined using RNA-Sequencing (RNA-Seq) and the expression of relevant genes was further validated using reverse transcriptase quantitative PCR (RT-qPCR), Western blot and immunocytochemistry. PROX1 depletion resulted in a decreased cell motility, with both migratory and invasive potential being significantly reduced. The cell morphology was also affected, while the other studied cancer-related cell characteristics were not significantly altered. RNA-seq analysis revealed significant changes in the expression of transcripts encoding genes involved in both motility and cytoskeleton organization. Our transcriptional analysis of *PROX1*-depleted follicular thyroid carcinoma cells followed by functional and phenotypical analyses provide, for the first time, evidence that PROX1 plays an important role in the metastasis of thyroid cancer cells by regulating genes involved in focal adhesion and cytoskeleton organization in tumour cells.

## 1. Introduction

Metastasis is a complex multistep biological process during which cancer cells leave the site of the primary tumour and migrate to distant anatomic sites where they proliferate and form secondary tumour foci. Metastatic spreading of cancer cells occurs via vascular and/or lymphatic systems. Even though the mechanisms and pathways of cancer development and spreading have been extensively studied, the mechanisms of one of the most critical steps in cancer progression associated with a worse prognosis—infiltration of adjacent tissues by malignant tumours and spreading to form distant metastasis—remains to be elucidated. 

One of the working models of cancer spreading highlights the crucial role of the lymphatic system, which is in accordance with its basic function in the defence of the organism against various diseases and in the development of inflammatory states [1,2]. Nevertheless, molecular pathways associated with lymphatic invasion remain unclear [3,4]. A search for lymphangiogenesis biomarkers of lymphatic endothelial cells (LEC) has revealed several markers including prospero homeobox 1 (PROX1), podoplanin, vascular endothelial growth factor receptor 3 (VEGFR-3) and lymphatic vessel endothelial hyaluronan receptor 1 (LYVE-1) [5,6,7,8,9]. Further studies confirmed that these markers play a role also in tumorigenesis [10,11,12,13,14,15]. PROX1 is of particular interest among these markers because it additionally plays an essential role in other biological processes, such as cell differentiation, proliferation, migration and apoptosis.

PROX1 belongs to an evolutionarily conserved class of atypical homeodomain proteins and presents extensive homology to the Pros proteins of other species (e.g., *Caenorhabditis elegans*, *Mus musculus*, *Gallus gallus* and *Danio rerio*) [16,17,18,19,20]. It is mainly localized in the nucleus, but it is also commonly found in the cytoplasm and its function depends on its intracellular localization [21,22]. Apart from its role in lymphangiogenesis, PROX1 contributes to the progression of the neuroectodermal embryonic and lens cells [5,21,22,23,24]. Moreover, it has been shown to be an early specific marker of the liver and pancreas organogenesis. The absence of functional PROX1 disrupts the morphology of epithelial pancreatic cells and inhibits their growth. In the liver, PROX1 is necessary for the migration of hepatocytes and its loss leads to the development of smaller organs with a reduced hepatocyte cell population [25,26,27]. Last but not least, PROX1 seems to be implicated in tumorigenesis.

Many studies have described a significant impact of PROX1 on the biology of different cancer types, including tumours of the central nervous system and the liver, oesophageal squamous cancer, colon cancer, haematological malignancies as well as breast, pancreatic and thyroid cancers [15,28,29,30,31,32,33,34,35]. Furthermore, the *PROX1* gene is frequently inactivated by genomic deletions and epigenetic silencing in carcinomas of the biliary system [16]. The effect of PROX1 on the tumour development is strongly associated with its cellular localization, the tissue type and cancer stage. Therefore, in some cases PROX1 was reported to function as an oncogene whereas in some others as a tumour suppressor [28,36,37,38]. Still, PROX1 alone is likely not able to trigger tumorigenesis. However, it is capable of promoting tumour progression by disrupting cell polarity and adhesion [32,39].

Migration of cells is a fundamental phenomenon in cancer biology, which includes the linkage extension, creation of the new focal adhesions and translocation of cells. Over these events the actin filaments polymerize and lead to cytoskeleton reorganization what coordinates the cellular motility and in result the progression of cancer. We had previously shown that PROX1 stimulates motility of follicular thyroid cancer cells and that its expression is correlated with the rates of both migration and invasion. The suppression of PROX1 in FTC-133 cells resulted in decreased migration and invasion of these cells, deregulation of cytoskeleton and changes in the expression of some genes involved in the regulation of cell adhesion [37]. In the current study, we asked whether similar behaviour and phenotypic changes following PROX1 depletion could also be observed in other FTC-derived cell lines, which would suggest that PROX1 regulation is important in follicular thyroid carcinogenesis. We chose the CGTH-W-1 cell line, derived from a sternal metastasis of follicular thyroid carcinoma, because it expresses the highest PROX1 levels among the three previously tested cell lines: FTC-133, ML-1 and CGTH-W-1 (PROX1 expression in CGHT cells is about 2-fold higher than in FTC-133 cells) [37]. In order to gain more insight into the exact role of PROX1 in the biology of thyroid cancer cells, we have knocked down *PROX1* expression in this cell line and studied the effect of this silencing on malignant characteristics of the cells, such as migration, invasion, proliferation and survival. We have also analysed the effects of PROX1 on the transcriptional profile of CGTH-W-1 cells using RNA-seq analysis.

## 2. Results

### 2.1. PROX1 Knock-Down CGTH-W-1 Cells and Its Effect on Cell Motility and Invasive Potential

**Figure 1:**
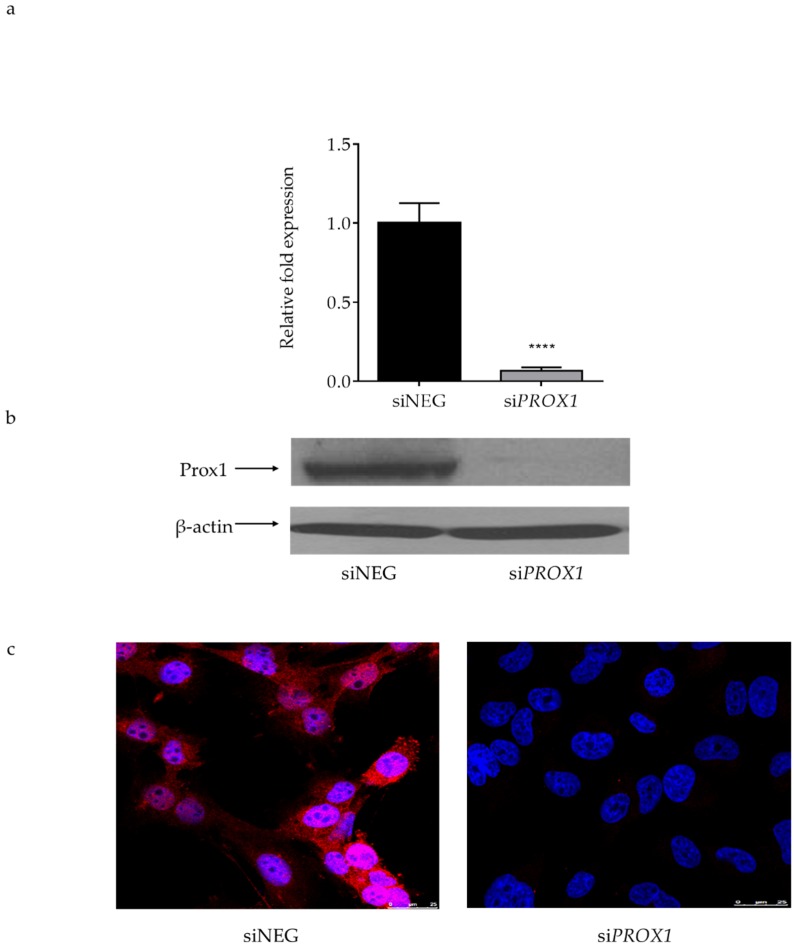
The efficiency of *PROX1* knockdown (48 h) with siRNA in CGTH-W-1 cells derived from follicular thyroid cancer (sternal metastasis). (**a**) Real time (RT)–qPCR analysis of *PROX1* knockdown efficiency in CGTH cells. The *ACTB* gene was used as a reference. Data represent means with standards deviations (SD) from five independent experiments; ****: *p* < 0.0001 when compared with siNEG-transfected cells. (**b**) Western blot analysis of *PROX1* silencing in CGTH-W-1 cells. β-actin was used as a loading control. (**c**) Immunofluorescent staining images corroborate the RT-PCR and Western Blot results. Fluorescent rhodamine staining (red) shows nuclear and cytoplasmic localization of the Prox1 protein expression, whereas DAPI (4′,6-diamidino-2-phenylindole, blue) stains the nuclei, (magnification: 400×, scale bar 25 um). Presented immunoblot and immunofluorescent staining images are representative of at least three independent experiments.

We examined the effect of PROX1 on the motility of the follicular thyroid carcinoma-derived CGTH cells by analysing their migratory and invasive potential, following the *PROX1* knock-down. The efficiency of the Prox1 depletion was confirmed by RT-qPCR, Western blot and immunocytochemistry analyses, showing an over 90% reduction in the PROX1 transcript and protein expression levels in CGTH cells transfected with siRNA-PROX1 but not in those transfected with the control, non-targeting siRNA (siNEG) (Figure 1).

**Figure 2:**
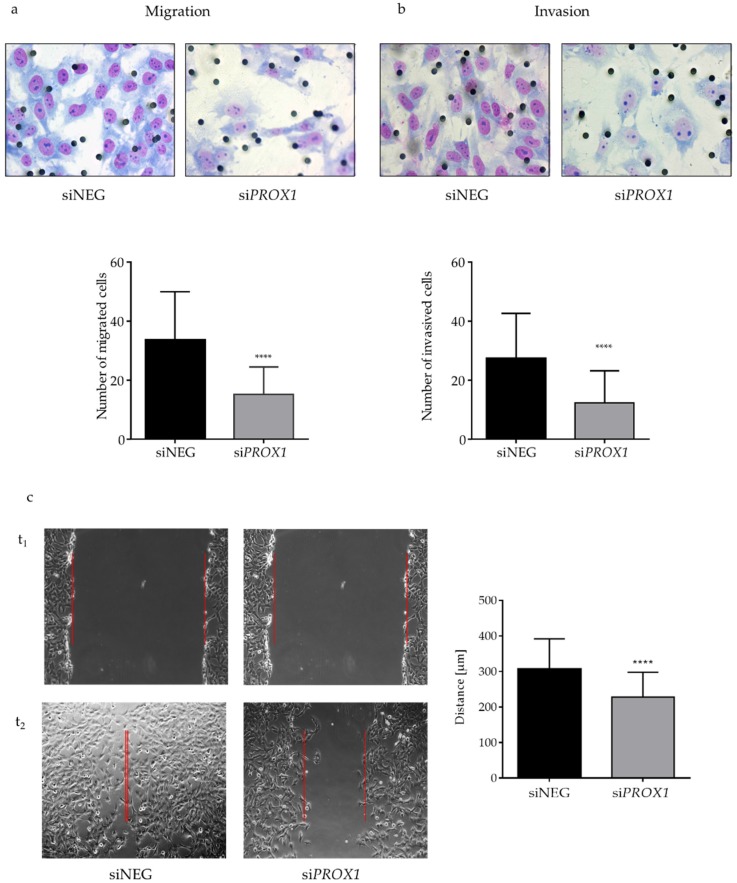
The effect of *PROX1* knock-down on the migration (**a**), invasion (**b**) and wound healing capacities (**c**) of CGTH-W-1 follicular thyroid cancer cells determined by Boyden chamber migration, Matrigel invasion and wound-healing assay, respectively. *PROX1* depletion significantly decreased the number of migrating and invading CGTH cells in the Boyden chamber assay. The *PROX1*–deficient cells significantly decreased wound width closure when compared with control CGTH cells transfected with siNEG (wound borders are marked with red color). To inhibit proliferation, cells were pre-treated with Actinomycin D for 30 min, washed with PBS, then wounded. Error bars represent means with standard deviations (SD). **** indicates *p* < 0.0001 when compared to the control. Each experiment was repeated at least three times and in triplicate. Images were captured at 0 (t1) and 24 h (t2) after the scratches were made under a light microscope (Olympus BX41, magnification ×400, scale bars 25 µm).

The strong reduction in PROX1 expression in the CGTH cells was associated with impaired migration and invasion capacities (Figure 2a,b). We observed a significant (*p*-value < 0.0001) over two-fold reduction in the number of migrating cells and an over two-fold decrease in the invasive capacity of the PROX1-depleted cells as compared to control siRNA-NEG transfected CGTH cells, as shown by the chamber migration assay (Figure 2a) and the Matrigel invasion assay (Figure 2b), respectively. A consistent and strong reduction in both migration and invasiveness of the *PROX1*-silenced cells was observed in each of the experimental replicates.

Next, in order to confirm the association between PROX1 silencing and the observed phenotypes, we evaluated the effect of *PROX1* suppression on motility of CGTH cells. To this end, classical wound healing assay was performed in the presence or absence of proliferation inhibitor actinomycin D. PROX1 suppression led to a statistically significant (*p*-value = 0.0001) inhibition of migration in the *PROX1*-silenced cells in comparison to the cells treated with control siRNA (Figure 2c), resulting in a 6.6-fold reduction in the number of migrating cells regardless of the presence of the proliferation inhibitor. Furthermore, we observed (immunofluorescence) that the loss of *PROX1* in CGTH cells induced phenotypical changes in the cytoskeleton, the cell shape and in the number and cellular distribution of the stress fibres and cellular protrusions.

### 2.2. Effect of PROX1 on Cell Viability, Cell Cycle and Apoptosis

It is well recognized that carcinogenesis occurs through acquisition and accumulation of multiple genetic changes that lead to uncontrolled proliferation of cells followed by developing invasive properties. Since PROX1 plays multiple roles in human cancer, displaying either oncogenic or suppressive activity depending on the cancer type, it may also exert variable effects on cancer cell proliferation, in particular given that migration and invasion capacities of cells also depend on their proliferative potential. Therefore, we determined whether PROX1 depletion influences the viability/proliferation, cell cycle and apoptosis in CGTH cells. To this end, we performed 3-(4,5-dimethylthiazol-2-yl)-2,5-diphenyltetrazolium bromide (MTT) viability assays as well cell cycle and apoptosis cytometric analyses. Despite significantly reduced migration and invasion capacities in PROX1-depleted CGTH cells, we did not detect a consistent effect of PROX1 suppression on cell viability (Supplementary Figure S1a). The CGTH cells did not respond to PROX1 knock-down with apoptosis induction (Supplementary Figure S1b) and the loss of PROX1 was not accompanied by the G_1_/S cell cycle arrest, either (Supplementary Figure S1c). 

### 2.3. PROX1 Expression Deficiency and Changes in Gene Expression Profiles

#### 2.3.1. RNA-seq Analysis 

**Figure 3:**
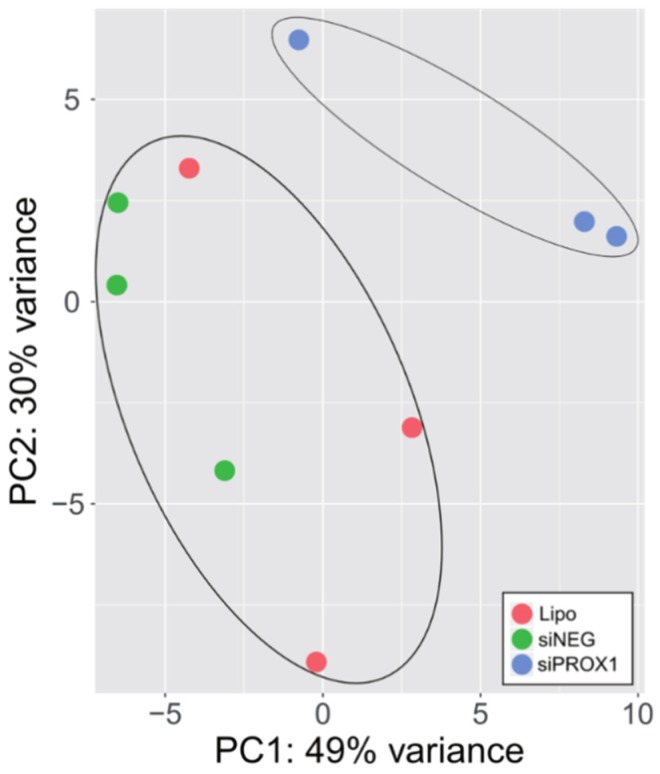
Principal component analysis (PCA) of RNA-Seq datasets for PROX1-depleted CGTH-W-1 cells. PCA was determined from abundance of all transcripts detected in RNA-seq survey. siNEG, siP*ROX1* and Lipo: cells transfected with non-targeting siRNA, with *PROX1* siRNA and with lipofectamine alone, respectively.

To elucidate transcriptome changes associated with *PROX1* depletion, we performed RNA-seq-based gene expression profiling of CGTH cells treated with siRNAs targeting *PROX1* (si*PROX1*), compared to those treated with non-targeting siRNA (siNEG) or Lipofectamine (Lipo) alone. Principal component analysis (PCA) discriminated studied samples by separating a cluster of si*PROX1* samples from Lipo and siNEG (Figure 3). In a comparison between Lipo and siNEG samples, we found a significantly altered expression for 209 genes (136 were upregulated and 73 downregulated; adjusted *p* value < 0.05) (Supplementary Table S3). These genes were regarded as a false positive off-target of a transfection procedure and were excluded from the siNEG *versus* si*PROX1* comparison set, which left a pool of 727 upregulated and 455 downregulated genes (adjusted *p* value < 0.05; Supplementary Table S4).

#### 2.3.2. Gene Ontology Analysis

**Figure 4:**
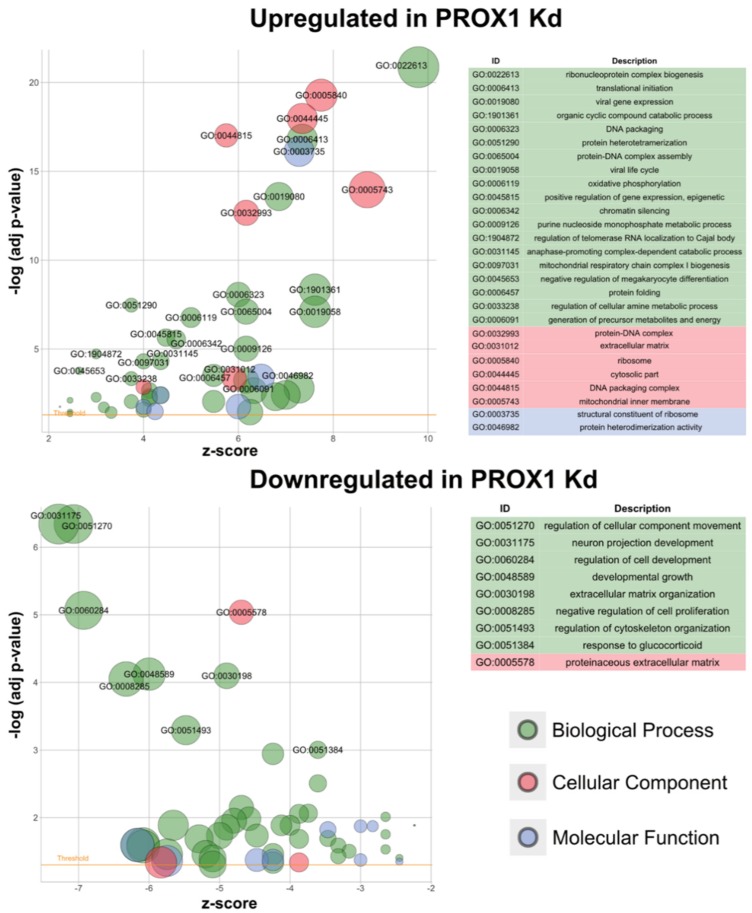
Bubble plots of significantly enriched Gene Ontology (GO) terms for the *PROX1*-depleted CGTH-W-1 cells. The cut-off for labelling was set at an adjusted *p*-value of 0.001. The z-score (horizontal axis) indicates whether the given term in more likely to be up- or down-regulated. The area of the displayed circles is proportional to the number of genes assigned to the term in the analysis. The threshold indicates the adjusted *p*-value of 0.05.

Further Gene Ontology (GO) enrichment analysis of the set of differentially expressed genes led to the identification of 71, 17, 15 and 55, 14, 5 of GO terms belonging to the biological processes, molecular function, cellular component ontology for up and downregulated genes, respectively, affected by *PROX1* depletion (adjusted *p* value < 0.01; Figure 4 and Supplementary Table S5). The top GO terms for biological processes ontology for the upregulated genes were related to ribonucleoprotein complex biogenesis, while the downregulated genes were associated with the regulation of cellular component movement, extracellular matrix organization and cytoskeleton organization. Therefore, the phenotypical changes in the morphology of cells upon *PROX1* depletion were also reflected in a significant enrichment of cytoskeleton-associated genes among the downregulated transcripts in RNA-seq dataset.

#### 2.3.3. Validation of RNA-seq Data

To confirm RNA-Seq-based gene expression data, we further analysed the CGTH control and *PROX1*-deficient cells with RT-qPCR (using the same RNA samples that were used for RNA-Seq), Western blot and immunocytochemistry techniques. Given the phenotypical changes observed in the cells upon *PROX1* depletion and the results of GO functional analyses of the RNA-Seq dataset, we focused on those differentially expressed genes which are known to play a role in the organization of the cytoskeleton as well as in migration and cell adhesion since these cellular features are well established hallmarks of carcinogenesis. To this end, we screened genes whose expression was found to be altered in RNA-Seq-based analysis (Supplementary Table S4).

First, we evaluated the expression levels of 58 genes which had been identified in RNA-Seq-based analysis, using a standard RT-qPCR technique (Table 1 and Table 2). For most genes, this analysis confirmed changes of the expression profiles which had been found using RNA-Seq. 

**Figure 5:**
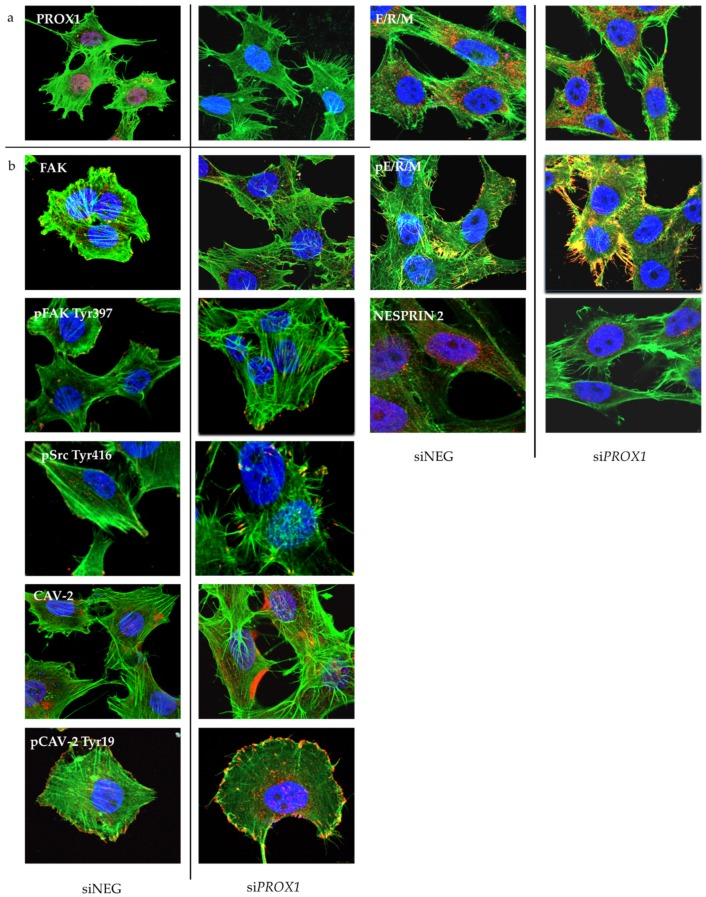
*PROX1* knock-down induced changes in the organization of the actin cytoskeleton, the cell shape, the filopodia extension and the cell-cell adhesion. Representative confocal microscopy images displaying expression levels and cellular localization of PROX1 (**a**) and the following immunofluorescently labelled signalling proteins involved in the cell migration, focal adhesion and cytoskeleton organization after *PROX1* silencing with PROX1-specific or negative siRNA (**b**) taken at a 1000× magnification are shown. siNEG: negative control (cells transfected with negative siRNA). CGTH cells were immunofluorescently labelled with DAPI (4′,6-diamidino-2-phenylindole; nuclear staining, blue), FITC-phalloidine (fluorescein isothiocyanate; F-actin, green) and with relevant DyLight^®^ 594 or with Rhodamine Red (red)-conjugated secondary antibodies.

**Figure 6:**
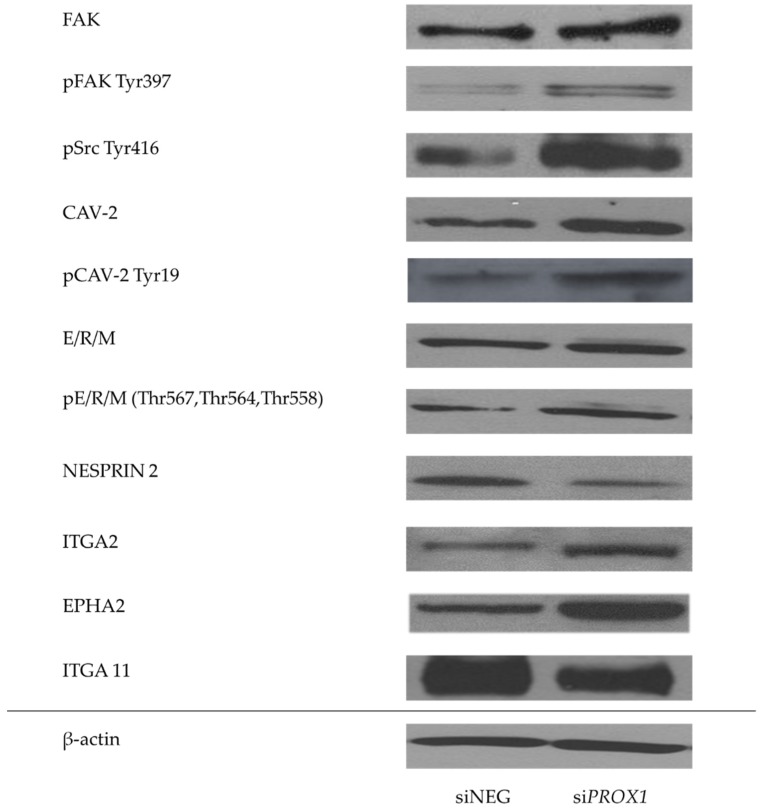
The evaluation of selected genes associated with organization of the cytoskeleton, migration and cell adhesion with significantly deregulated mRNA expression due to *PROX1* knock-down. The expression levels of proteins (FAK kinase protein, caveolin-2, ezrin, radixin and moesin, nesprin-2, integrin alpha 2, ephrin type-A receptor 2, integrin alpha 11) and phospho-proteins (phosphorylation of FAK at Tyr(tyrosine)-397, phosphorylation of proto-oncogene tyrosine-protein kinase Src at Tyr-416, phosphorylation of caveolin-2 at Tyr-19 and phosphorylation of ezrin at Thr(threonine)-567, radixin at Thr-564 and moesin at Thr-558) in CGTH cells transfected with si*PROX1* and siNEG RNA, as determined by Western blotting. The whole cell lysates were resolved using SDS-PAGE (sodium dodecyl sulphate-polyacrylamide gel electrophoresis), transferred on nitrocellulose membranes, then incubated with primary and relevant secondary antibodies and visualized with chemiluminescence detection substrate. β-actin was used as a loading control. Presented blots are representative of three independent experiments.

Afterwards, we performed a detailed immunocytochemistry (Figure 5) and Western blot (Figure 6) evaluation of selected genes at the protein level, in particular several genes associated with migration, focal adhesion, cytoskeleton structure regulation and cell morphology which were identified by both RNA-seq and RT-qPCR as having significantly deregulated transcript levels following PROX1 suppression. These were the following: the *PTK2* gene (encoding the focal adhesion kinese, FAK kinase protein), *CAV2* (caveolin-2), *SYNE2* (nesprin 2), *ITGA2* (integrin alpha 2), *ITGA11* (integrin alpha 11) and *EPHA2* (ephrin type-A receptor 2). We found that protein expression patterns obtained by Western blot and immunocytochemistry analyses directly corresponded to RNA-Seq and RT-qPCR data for all of the tested genes (Figure 5 and Figure 6). The protein levels of FAK kinase and caveolin-2 as well as of ezrin, radixin and moesin (ERM) proteins, all associated with focal adhesions and cytoskeleton regulation, paralleled increased mRNA expression levels, while the expression of *SYNE2* encoding the nesprin 2 protein that links the cytoskeleton and nucleoskeleton with the nuclear membrane was significantly downregulated in PROX1-deficient cells as compared to control cells (Figure 5). The expression of active, phosphorylated form of FAK, caveolin-2 proteins was also significantly increased (Figure 5). An immunofluorescent analysis of their cellular localization revealed that FAK, Cav-2 and ERMs in the PROX1-deficient CGTH cells localized mainly in the membranes at focal adhesions and in cellular protrusion. 

Then, we analysed the activation levels of FAK and CAV-2 measuring relative expression levels of their phosphorylated forms. We found that lack of PROX1 affected not only the basal expression levels of FAK and CAV-2 but also their activation status in CGTH cells. The expression level of major phosphorylation site Tyr (tyrosine) 397 of FAK was considerably higher when compared to cells transfected with siNEG. Since the FAK Tyr 397 autophosphorylation site is coupled with Src kinase activity, we evaluated the expression of activated proto-oncogene tyrosine-protein kinase Src-Tyr 416. We found that the levels of activated Src kinase phosphorylated at Tyr 416 were also much higher in PROX1-depleted cells than in controls. In the same line, the caveolin-2 phosphorylated at Tyr 19 expression was greater in PROX1-deprived cells. 

Finally, we verified the activation status of the other proteins which are crucial for cell adhesion and organization of cell cortex: ERM proteins. These are three related proteins which function as linkers between membranes and cytoskeleton, coupling transmembrane proteins to the actin cytoskeleton through a common FERM (four point one, ERM) domain. 

Although lack of *PROX1* did not affect basal expression levels of these proteins, we found that their phosphorylation was significantly higher in *PROX1*-deficient cells (Figure 5 and Figure 6). Loss of *PROX1* expression also significantly influenced the focal adhesions’ location and density. Immunofluorescent staining showed that phosphorylated forms of Src kinase, FAK, Cav-2 and ERM proteins abundantly localized at the numerous focal contacts localized along actin stress fibres at the numerous elongated focal adhesions and at the ends of cell protrusions (Figure 5).

Moreover, we observed a significant reduction in the expression of integrin (ITGA11 expression and a significant increase in the expression of ITGA2 after *PROX1* silencing in the CGTH cells (Figure 6). We have also found significantly enhanced protein levels of ephrin type A receptor 2 (*EPHA2*) in CGTH cells with silenced *PROX1*. 

Western blot analysis confirmed immunocytochemistry results, showing that PROX1 suppression leads to an increased expression of FAK, Caveolin 2, ERMs, ITGA2 and EPHA2 and a reduced expression of Nesprin2 and ITGA11 proteins, as well as to an increased expression of phosphorylated forms of FAK, Cav-2, SRC kinase and ERM proteins (Figure 6). Overall, these results indicate that loss of PROX1 in follicular thyroid cancer cells induces significant changes in the expression of genes involved in cellular focal adhesion, dynamic cytoskeleton transformation, determination of cell shape and migration.

### 2.4. PROX1 Expression in Human FTC Tissues 

**Figure 7:**
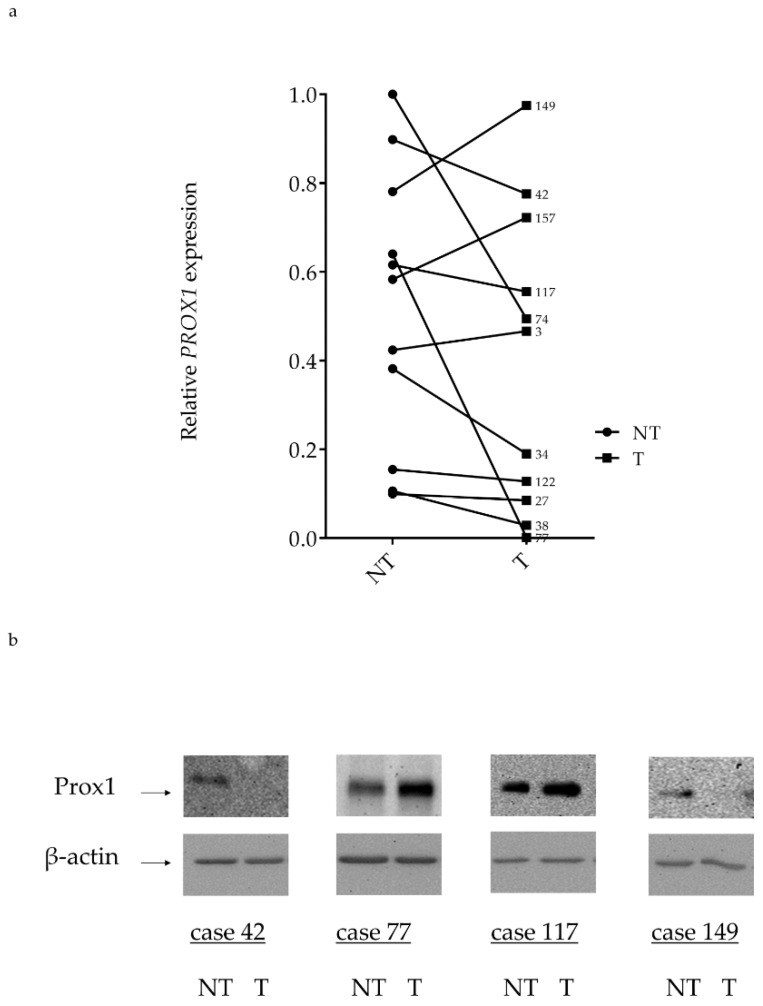
The expression of *PROX1* was examined in human follicular thyroid cancer and non-cancerous tissues by RT-qPCR and Western blot and shows variability without correlation with protein expression level. (**a**) Relative *PROX1* mRNA expression in 11 FTCs (T) and paired non-tumoral tissues (NT) analysed by RT-qPCR. PROX1 expression level is reported as Ct values normalized to corresponding Ct values of the housekeeping gene (ACTB). (**b**) PROX1 protein was evaluated in selected human FTCs cases and non-tumoral paired control tissues using Western blotting with anti-Prox1 antibody and β-actin as a loading control.

**Figure 8:**
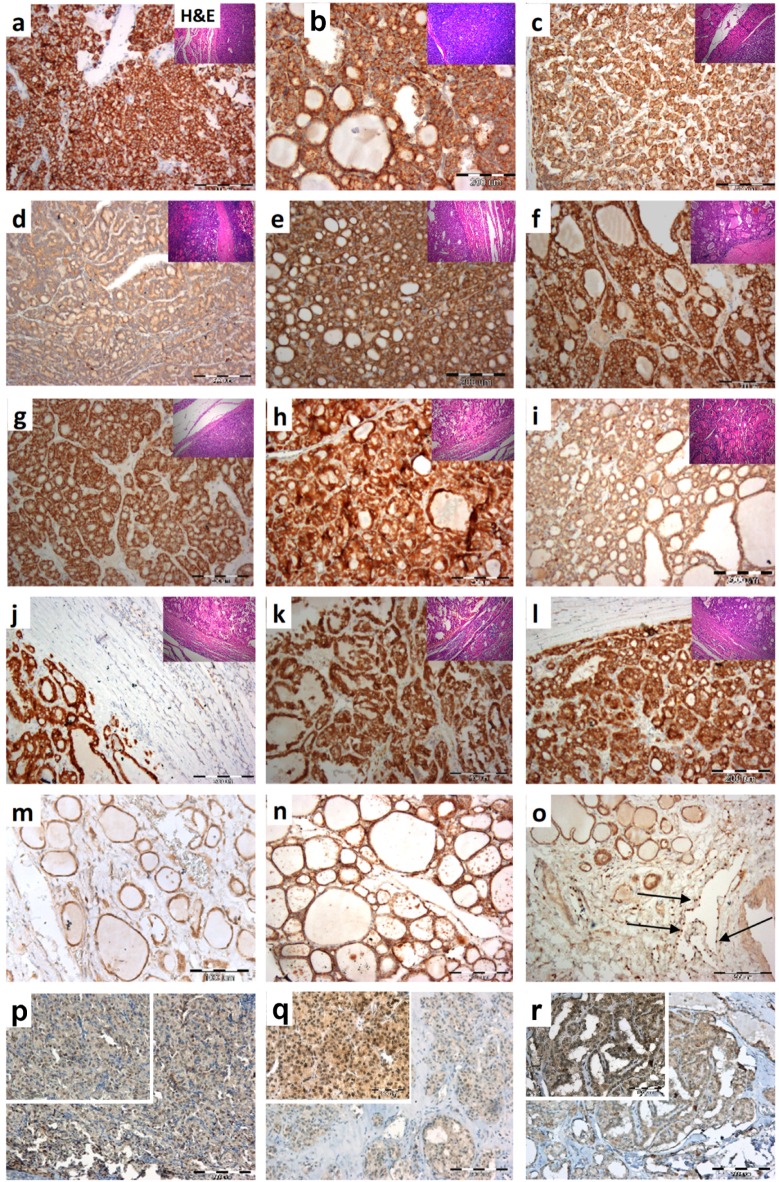
Analysis of PROX1 expression in human FTC tissue sections. Representative immunohistochemistry images of PROX1 expression (**a**–**l**) showing variable intensity of immunostaining. In the normal thyroid (**m**,**n**), the PROX1 labelling was mostly uniform in both follicles and epithelial cells. Strongly stained lymphoendothelial cells (LEC) (arrows) that showed intense immunoreactivity to PROX1 were used as an internal positive control (**o**). Metastases of FTC to the lungs (**p**–**r**). Tumour sections were stained with anti-PROX1 antibody as described in the Materials and Methods section. Original magnification: a–l, m, n × 200; p,q,r-insets × 400, o × 400; H&E ×100. Insets in the images–haematoxylin and eosin (H&E) staining of analysed FTC tissues. TNM of examined FTCs: a-pT2N0 minimally invasive; b-pT3N0 minimally invasive; c-pT2N0 minimally invasive; d-pT2N0 widely invasive; e-pT3N0 minimally invasive; f-pT3N0 minimally invasive; g-pT3N0 minimally invasive; h-pT3N0 minimally invasive; i-pT3N0, widely invasive; j-pT1a minimally invasive; k-pT3N0 minimally invasive; l-pT3No minimally invasive; average age at diagnosis (range) 57,1 (37–78).

Finally, PROX1 expression was evaluated in human follicular thyroid cancer using RT-qPCR and Western blotting in the case of frozen specimens and immunohistochemistry for archived tissues (Figure 7a,b and Figure 8). The expression of PROX1 transcript in FTC tissues revealed high variability, where among 11 samples some cases showed differently reduced PROX1 or very low mRNA level and the others revealed similar or even higher PROX1 expression than paired non-tumoral tissue (Figure 7a). Moreover, transcript levels did not correlate with protein expression (Figure 7b). 

Next, PROX1 expression and tissue distribution were evaluated by immunohistochemistry in archived paraffin-embedded samples from 20 FTC cases. Representative immunostaining is shown in Figure 8a–l. All analysed tissues were positive for PROX1 protein expression with variable staining intensity mainly showing uniform distribution in the cytoplasm. LEC cells used as internal positive controls showed intense PROX1 staining (Figure 8o). In the normal thyroid (Figure 8m,n), PROX1 protein was distributed in both follicles and thyrocytes. We also examined the expression of PROX1 protein in three cases of FTC metastases to the lungs and, as shown in Figure 8p–r, the strong immunostaining of PROX1 was detected in most of the nuclei with weak or moderate cytoplasmic reactivity (Figure 8p–r insites).

## 3. Discussion

The homeobox gene *PROX1* plays a key role in the embryonic development of different organs such as the lens, liver, pancreas, central nervous system and lymphatic system and may function as a suppressor gene or an oncogene, depending on the tissue and cancer type [22]. While PROX1 expression is associated with tumour development and metastasis, its exact function in the process of tumorigenesis remains unclear.

We reported previously levels of *PROX1* mRNA in follicular thyroid carcinoma-derived cells as compared to cells derived from papillary thyroid cancer that may relate to follicular thyroid cancer progression and enhanced invasion [37]. The PROX1 protein expression influenced in vitro migratory and invasive capacities and cytoskeleton deregulation but did not affect other classic hallmark characteristics of tumour cells, such as proliferation, apoptosis or cell cycle. However, it triggered the expression of several genes, including *PTK2/FAK*, *CAV1*, *CAV2* and others associated with the regulation of cytoskeleton and cell focal adhesion, causing observable changes in cell shape. The aim of this study was to determine the molecular signature of PROX1 in follicular thyroid cancer and to perform functional analyses of the CGTH cells following *PROX1* silencing. CGTH cells, which were derived from a sternal metastasis of follicular thyroid carcinoma, express the highest levels of *PROX1* among the three tested FTC-originating cell lines: FTC-133, CGTH-W1 and ML1. 

We found that PROX1 depletion in CGTH cells significantly suppressed their in vitro motility, migration and invasion properties, whereas cell viability, proliferation and apoptosis were not changed. In fact, the expression of *AKT* and *ERK1/2*, key genes of proliferation signalling pathways, was not affected by PROX1 depletion (data not shown), consistently with our previous findings for FTC-133 cells [37]. Thus, these two cell lines, derived from different patients and possibly differing in their molecular contexts but showing the same response to PROX1 depletion, supports the hypothesis that PROX1 plays a role in thyroid carcinogenesis, being involved in regulating invasion and metastasis but not in determining the cells’ fate. However, studies on the role of PROX1 in tumorigenesis of other cancer types, like glioblastoma [40] and gastric cancer [41], reported results contradictory to ours and the observed effect of PROX1 may be specific for thyroid cells.

RNA-seq profiling in PROX1-depleted CGTH cells revealed that nearly 1200 genes were affected by the *PROX1* knock-down. We further confirmed RNA-seq data by examining the expression of 58 selected genes by RT-qPCR and in 98% of cases the PCR data were consistent with the RNA-seq. Such a broad spectrum of deregulated genes may indicate that PROX1 plays an important role in the metabolism of follicular cancer cells. However, our analysis does not identify a *bona fide* PROX1 gene targets and it is likely that altered expression levels of many of these genes is due to an indirect effect of the PROX1 depletion. Such identification would require comparison of the expression data with PROX1 chromatin immunoprecipitation genome wide (ChIP-seq) distribution but such a dataset is not available for CGTH cells. 

Next we found that the expression of several kinases was significantly changed, including a decreased expression of the Rho associated coiled-coil containing protein kinase 1 (*ROCK1*), pseudopodium enriched atypical kinase 1 (*PEAK1*), phosphatidylinositol-4-phosphate 5-kinase type 1 gamma (*PIP5K1C*) and doublecortin like kinase 1 (*DCLK1*), all being involved in the cytoskeleton organization, angiogenesis and metastasis. 

The focal adhesion dynamics and the cytoskeletal rearrangement as well as signalling of cell survival and motility involve two key non-receptor tyrosine kinases: FAK and Src kinase. Located in complexes of focal adhesion, FAK and Src interact and activate one another. This FAK-Src signalling complex contributes to regulating focal adhesion dynamics and turnover, actin-cytoskeletal structures, cytoskeleton rearrangement and cell morphology whereas Src catalytic activity controls the turnover of focal adhesion structures during cell motility [42]. 

Similarly, the expression of active forms of ERM proteins significantly increased following the *PROX1* knock-down. ERM proteins are located at filopodia, microvilli and adhesion sites of cells and are involved in regulating such cellular processes as reorganization of actin cytoskeleton, membrane dynamics, cell adhesion and migration [43]. It seems that a high expression of phospho-ERM at cellular protrusions is the cellular response to the stress following *PROX1* silencing. These results were concordant between Western blot and immunofluorescence analyses. However, we did not analyse the expression levels of ezrin, radixin and moesin separately and, therefore, we do not know whether the expression of any of these proteins alone constitutes a molecular signature specific for follicular thyroid carcinoma and contributes to the observed cytoskeletal, morphological and adhesive changes, or whether this is a result of all the three ERM proteins acting together.

Another protein highly deregulated after *PROX1* silencing was caveolin-2 but not caveolin-1, one of the major structural components of detergent-resistant cholesterol-rich membrane invaginations (caveolae). The caveolin-2 function may be cell- and tissue-specific [44]. The phosphorylated caveolin-2, pCAV-Tyr19, unlike its unphosphorylated form, co-localized with activated FAK mainly near or at focal adhesions associated with the ends of stress fibres, confirming that phospho-CAV-2 may play a role during signal transduction [45]. Moreover, we found that the high expression of caveolin-2 associated with changes in the cytoskeleton organization and cell shape in PROX1-depleted cells implicating a role of phosphorylated CAV-2 in the regulation of both cytoskeleton rearrangement and cell shape, also during thyroid carcinogenesis [46]. Our immunocytochemistry results suggest that PROX1 depletion induces the formation of multiprotein complexes at the ends of protrusions as active forms of several of the analysed proteins.

Silencing *PROX1* has also altered the expression of other proteins controlling cell morphology and migration, such as nesprin 2, ITGA2 (integrin alpha 2), ITGA11 (integrin alpha 11) and EPHA2 (ephrin type-A receptor 2). In particular, we observed a significant reduction of nesprin 2 expression at nuclear membranes following the PROX1 depletion. Nesprins link the cytoskeleton and nucleoskeleton with the nuclear membrane and play an important role in nuclear positioning during various cellular processes, including cellular response to stress. These proteins affect cellular architecture playing a role in cellular signalling [47,48,49]. 

ITGA2 and ITGA11 are transmembrane receptors for extracellular matrix (ECM) fibrillar collagens and related proteins that mediate cell adhesion to the extracellular matrix, *PROX1* silencing in the CGTH cells significantly reduced expression ITGA11 and enhanced the expression of ITGA2. Although ITGA2 has been shown to be a target gene for certain microRNAs in aggressive papillary thyroid carcinoma [50], the impact and significance of its increased expression in follicular thyroid carcinoma cells upon *PROX1* silencing, like that of the ITGA11 downregulation, remains to be clarified.

EphA2 is a member of the largest family of receptor tyrosine kinases that are implicated in tumorigenesis, regulating transformation of normal cells, metastasis and angiogenesis [51]. Overexpression of EPHA2 was observed in cell lines derived from many aggressive human cancers and is frequently associated with enhanced angiogenesis and invasion [52]. The protein level of *EPHA2* was increased in CGTH cells after *PROX1* silencing. In fact, downstream target molecules of EPHA2, including FAK/Src kinases, are critical regulators of the cancer progression and a pronounced increase of active FAK/Src form expression associates with the *PROX1* knock-down. Therefore, we may hypothesize that EPHA2 signalling regulates cell adhesion and spreading in follicular thyroid cancer cells in a FAK/Src-dependent manner. 

Functional GO analyses allowed us to group genes based on their cellular function and to identify three major signalling pathways affected by PROX1 deficiency—a pathway controlling cytoskeleton organization, cell migration and cell adhesion. These findings are in accordance with phenotypical changes observed in functional cellular assays which showed changes in the cytoskeleton as well as altered migratory and adhesive properties in PROX1-deficient cells. Of note, to the best of our knowledge, the current study is the first study ever to report a genome-wide analysis of gene expression in PROX1-deficient cells. 

Finally, we observed that PROX1 is expressed in most of the examined FTC cases with high inter-individual variability, whereas all archived cancer tissues showed cytoplasmic and homogenously distributed PROX1 protein staining. Cellular localization of PROX1 however, is tumour tissue-dependent, as PROX1 can be localized in the cytoplasm, in the cell nucleus or both the cytoplasm and the nuclei [28,34,53,54]. Noteworthy, PROX1 in FTC tissues was cytoplasmic, whereas in the metastases of FTCs was detected mostly in the nuclei. Although cytoplasmic PROX1 may not exert its transcription factor activity, recent reports have shown that evaluation of cytoplasmic PROX1 in cancer patients may have a predictive value [34,55]. Nevertheless, the exact role of cytoplasmic PROX1 expression is still unknown. The hypothesis related to the presence of PROX1 in the cytoplasm of cancer cells hint at a possibility of enrichment and activation of PROX1 in the cytoplasm before being translocated to the nucleus to become functionally active [55]. Although decrease of PROX 1 expression in various thyroid cancers tissues was presented, there was a significant variability in the PROX1 expression in FTC cases [56,57,58]. Moreover, whereas in PTC (papillary thyroid cancer) derived cell lines the PROX1 transcript level was negative or negligible in all three FTC derived cell lines (FTC-133, CGTH and ML1 tested by us), the PROX1 mRNA was present at different level [37]. In addition, PROX1 inactivation seen in PTCs promotes the malignant behaviour of thyroid carcinoma and suggest that PROX1 reactivation could be considered as potential therapeutic factor [26]. The current study show that decrease of PROX1 expression suppress the malignant features of follicular thyroid cancer cells such as migration, motility and invasion. These results parallel the previous one, where FTC-133 cells were analysed [37], and suggest that in follicular thyroid carcinomas, PROX1 might contribute to their progression. 

In conclusion, we have shown that silencing *PROX1* expression strongly reduces motility and invasive potential of CGHT cells but does not affect their proliferation, apoptosis/necrosis and cell cycle. The PROX1 protein seems to contribute to follicular thyroid cancer metastases mainly by regulating cytoskeleton, focal adhesion and migration controlling pathways. Further studies should focus on the identification of cellular context-dependent signalling pathways regulated by PROX1 in follicular thyroid carcinoma. 

## 4. Materials and Methods 

### 4.1. Thyroid Cell Line 

The study was performed on CGTH-W-1, (hereafter referred to us as CGTH) a thyroid carcinoma cell line derived from follicular thyroid cancer (metastasis to sternum; obtained from the German Collection of Microorganisms and Cell Cultures, DSMZ, Braunschweig, Germany). The cells were put in culture from frozen stocks two passages before the experiments and maintained in culture for a maximum of ten passages. They were cultured in complete RPMI-1640 medium (ThermoFisher Scientific, Rockford, IL USA) supplemented with 10% foetal bovine serum (FBS, ThermoFisher Scientific) at 37 °C in a humidified 5% CO_2_ atmosphere. They were regularly tested for the presence of mycoplasma contamination using a PCR-based method as described elsewhere [59]. 

### 4.2. Silencing PROX1 by Small Interfering RNA

The PROX1 gene was silenced in the CGTH cells by transfection with siPROX1 targeting human PROX1 (MISSION esiRNA human Prox1, Sigma-Aldrich, Darmstadt, Germany) using the Ambion Silencer siRNA Transfection II Kit (ThermoFisher Scientific) according to the manufacturer’s protocol. Cells were trypsinized and suspended in complete growth medium (RPMI-1640 medium supplemented with 10% FBS) to a concentration of 1 × 10^5^ cell/mL. The cells were kept at 37 °C while the transfection complexes were prepared. Next, 100 µL of the transfection mix (30 nM siRNA in OptiMEM (ThermoFisher Scientific) plus 5 μL of Lipofectamine 2000 (Life Technologies, Carlsbad, CA, USA)) was added to 2 mL of 1 × 10^5^ CGTH cells (in complete RPMI-1640 medium supplemented with 10% FBS) and the cells were seeded in 6-well plates. Cells were incubated for 48 or 72 h at 37 °C in a humidified 5% CO_2_ atmosphere. Control cells were transfected with negative siRNA (siNEG; MISSION siRNA, SIC-001, Sigma-Aldrich). Cells transfected with the transfection mix without any siRNA (Lipofectamine only, Life Technologies) were used as a technical negative control. The experiments were conducted in triplicates and at least three times. Before further processing cells were silenced for 48h, unless otherwise indicated. Each time PROX1 knockdown was verified by qPCR.

### 4.3. RNA Extraction and cDNA Synthesis

Total RNA was extracted from cultured cells 48 h after the transfection using GeneMATRIX Universal RNA Purification Kit (EURx, Gdansk, Poland), followed by an on-column DNase A digestion (A&A Biotechnology, Gdynia, Poland) according to the manufacturer’s protocol. The concentration and purity of RNA samples were determined using the Synergy 2 Multi-Mode Reader (BioTek Instruments, Vinooski, VT, USA). cDNA was synthetized from 1 µg of total RNA using the High-Capacity cDNA Reverse Transcription Kit with RNase Inhibitor (ThermoFisher Scientific) according to the manufacturer’s protocol.

### 4.4. Real-Time Quantitative PCR (RT-qPCR)

The expression of fifty-eight genes involved in regulating basic cellular characteristics and processes (cytoskeleton organization, cell motility, invasive potential, adhesion) was quantified by RT-qPCR. Each reaction contained: 2 µL of 10-fold diluted cDNA, 10 µL of 2× DNA-specific SYBR Green dye (Maxima SYBR Green/Fluorescein qPCR Master Mix, Fermentas, Vilnius, Lithuania) and 10 nM of specific oligonucleotide primers (Supplementary Table S1) in a total volume of 20 μL. PCR reactions were performed in triplicates on the iQ5 Real-Time PCR Detection System (Bio-Rad, Hercules, CA, USA) using the following conditions: 30 s at 95 °C, followed by 40 amplification cycles (95 °C for 5 s, 58 °C for 15 s, 72 °C for 10 s). The Ct values were normalized to the corresponding Ct values of the *ACTB* reference gene. 

### 4.5. RNA-Seq Library Preparation and Sequencing

The integrity of the extracted RNA was verified on the 2100 Bioanalyzer (Agilent Technologies, St. Clara, CA, USA) using the Agilent RNA 6000 Nano Kit. Samples with RNA integrity numbers (RIN) above eight were used to construct a cDNA library. Ion AmpliSeq Transcriptome Human Gene Expression Panel (ThermoFisher Scientific) was used for library preparation according to the manufacturer’s protocol [60]. Briefly, RNA was reverse-transcribed, and the cDNA was subjected to multiplex PCR reactions to amplify fragments of the targeted transcripts. The amplicons were then partially digested at primer sequences followed by the adapters’ ligation to amplicons and purification on AMPure XP beads (Beckman Coulter Inc., Brea, CA, USA). The created library was quantified on 2100 Bioanalyzer and diluted to 80 pM prior to template preparation. Up to eight barcoded libraries were subjected to automated template preparation with Ion PI IC 200 Kit (ThermoFisher Scientific) on the Ion Chef Instrument (ThermoFisher Scientific). Barcoded RNA-seq libraries’ samples were sequenced on a PI chip in Ion Proton (Ion PI IC 200 Kit, ThermoFisher Scientific) according to the manufacturer’s instructions. Raw reads were processed by Torrent Suite analysis pipeline and mapped to human genome assembly hg19 AmpliSeq Transcriptome version by Torrent Mapping Alignment Program TMAP. Reads corresponding to each gene were counted with htseq-count v (Available online: https://htseq.readthedocs.io/en/release_0.11.1/) [61]. 

Normalization and differential expression were conducted by DESeq2 (Available online: https://doi.org/doi:10.18129/B9.bioc.DESeq2) using default parameters and options as described elsewhere [62]. Transcripts with adjusted *P*-value below 0.05 were considered to be statistically significant. Over-representation of Gene Ontology terms with hypergeometric test was determined by the R BioConductor package clusterProfiler (Available online: https://doi.org/doi:10.18129/B9.bioc.clusterProfiler) as described elsewhere [63]. GO visualization was conducted with the GOplot R package [64]. The expression data have been deposited as BAM files in the GEO database (Available online: https://www.ncbi.nlm.nih.gov/geo/) with the entry number GSE80135.

### 4.6. Western Blotting

Forty-eight hours after transfection, the cells were harvested, washed with phosphate-buffered saline (PBS) and lysed with RIPA lysis buffer (150 mM sodium chloride, 1% nonidet P40 (NP-40), 0.5% sodium deoxycholate, 0.1% SDS, 50 mM Tris; pH 8.0) supplemented with 1× protease and phosphatase inhibitor cocktails (cOmplete Protease Inhibitor Cocktail Tablets, Roche Diagnostics GmbH, Mannheim, Germany and Pierce Phosphatase Inhibitor Mini Tablets, ThermoFisher Scientific, respectively) and viscolase (according to the manufacturer’s protocol, A&A Biotechnology, Gdynia, Poland). The protein concentrations were determined using the Bradford assay (Sigma-Aldrich). Then, 30 μg of protein per well were separated by electrophoresis in 8–10% SDS–PAGE gels and transferred to nitrocellulose membranes (Bio-Rad). The blots were blocked with 5% non-fat milk or 5% bovine serum albumin (BSA) in PBS—0.05% Tween 20 (PBST), for one hour and probed with specific primary antibodies (Supplementary Table S2) for 12 h at 4 °C. Next, the membranes were washed with PBST buffer (4×) and incubated with appropriate HRP-conjugated secondary antibodies (Supplementary Table S2). The signals were visualized with a chemiluminescence detection substrate (SuperSignal West Dura Extended Duration Substrate, ThermoFisher Scientific). β-actin was used as a loading control.

### 4.7. Immunocytochemistry

Transfected cells were grown for 72 h on glass coverslips, washed with PBS and finally fixed with 4% paraformaldehyde at room temperature for 15 min. Next, the cells were permeabilized with 0.25% Triton X-100 in PBS (10 min) and then non-specific fluorescence was quenched using the Image-iT^®^ FX Signal Enhancer reagent (Life Technologies, Eugene, OR, USA) for 30 min and finally the non-specific sites were blocked with 3% BSA in PBS. Subsequently, the slides were incubated with a specific primary antibody (Supplementary Table S2) resuspended in 2% BSA in PBS (overnight at 4 °C). After several washes with PBST, the cells were incubated with a secondary fluorochrome-conjugated antibody (Supplementary Table S2), diluted in 3% BSA in PBS for one hour in the dark. 

For visualization of actin filaments, the samples were additionally stained for one hour with 2 µg/mL phalloidin-fluorescein isothiocyanate (FITC; Sigma-Aldrich, Merck KGaA, Burlington, MA, USA). Nuclei were stained with 4′6-diamidino-2-phenylindole (DAPI, Sigma-Aldrich) for two minutes. The signals were then analysed under a fluorescence microscope (AxioObserver D1, Thornwood, NY, USA) or a laser scanning confocal microscope (LSM800, AxioObserver Z.1) and processed with the AxioVison LE or ZEN-2.1 software, respectively (both microscopes and software from Carl Zeiss, Oberkochen, Germany). 

### 4.8. Cell Migration and Matrigel Invasion Assays

Cell migration and invasion assays were performed using Boyden insert chambers with 8 µm pore polycarbonate membranes, either uncoated (for migration assay) or coated with Matrigel (for invasion assay; both from Becton-Dickinson, Franklin Lakes, NJ, USA). Harvested cells were resuspended in a serum-free medium and counted with an EVA Automatic Cell Counter (Nano EnTek, Seoul, Korea). 1 × 10^5^ cells were seeded into each Boyden insert chamber, while medium enriched with 10% FBS was added to the external compartment of the 24-well plate. After 24 h of incubation at 37 °C in a humidified 5% CO_2_ atmosphere, cells which had migrated through the membrane were fixed and stained using the Diff-Quick Kit (Medion Grifols Diagnostics AG, Düdingen, Switzerland). Five fields from every membrane were photographed at a 400× magnification using an Olympus BX41 microscope (Olympus, Carlsbad, CA, USA) and the number of cells was counted using the ImageJ software (Available online: *www.rsbweb.nih.gov*). All experiments were repeated three times.

### 4.9. In Vitro Wound Healing Motility Assay

Cells were grown in 6-well plates to a confluent monolayer. Then, scratches of comparable dimensions were made on this monolayer of cells using a sterile 200 µL pipette tip. The cells were then washed twice with PBS to remove free-floating cells and cell debris and incubated in fresh medium for 24 h. To inhibit proliferation, cells were incubated with actinomycin D (1 µ/mL) for 30 min before the wound was made. Immediately after wounding (time 0 h) and at 6-h intervals during these 24 h, images of the cells were taken using a phase contrast microscope (100× magnification; AxioObserver D1) to determine the speed of wound closure. The width of the wounds was measured in five independent fields. The distance of cell migration was determined by measuring the width of the wound (ImageJ software) divided by two and by subtracting this value from the initial half-wound width. Distance was quantified as follows: with the 10× lens, 1 pixel = 1.026 µm.

### 4.10. Cell Viability Assay

Cell proliferation was evaluated by counting viable cells using the MTT (3-(4,5-dimethylthiazol-2-yl)-2,5-diphenyltetrazolium bromide) colorimetric assay (MTT Cell Growth Assay Kit, Merck Millipore, Burlington, MA, USA) 24, 48 and 72 h after transfection with siRNA. Cells were seeded in 96-well plates at a density of 2 × 10^4^ cells per well, with five replicates prepared for each time point on independent plates. Then, 10 µL of the MTT reagent were added to each well and cells were incubated for another 4 h. Subsequently, the assay was performed according to the manufacturer’s instruction. The optical density (OD) was measured at 570 nm using a Labsystems Multiscan RC micro-plate reader (Labsystems, Helsinki, Finland).

### 4.11. Apoptosis and Cell Cycle Analysis 

All samples were analysed with a flow cytometer (FACSCanto II cytometer with FACSDiva software, BD Biosciences, San Jose, CA, USA) to assess cell cycle and apoptosis. The experiments were performed in three replicates. Viability of the cells was estimated using the Annexin V-FITC Apoptosis Detection Kit (Abcam, Cambridge, UK) according to the recommended protocol. Briefly, harvested cells were washed with PBS, incubated with FITC-Annexin V and propidium iodide (PI) for 5 min at room temperature and analysed. Final apoptotic cell number was estimated as a total percentage of early apoptotic cells staining positive for Annexin V and negative for PI and late apoptotic cells positive for both Annexin V and PI.

For the cell cycle distribution patterns, 3 × 10^5^ CGTH cells harvested by trypsinization were washed with PBS and fixed in 70% cold ethanol at −20 °C overnight. Following fixation, cells were washed twice with PBS and stained with 5 µg/mL of propidium iodide in PBS containing 0.1% NP-40 and 10 µg/mL of DNase-free RNase. After 30 min at room temperature in the dark, the cell cycle distribution was analysed on a FACSCanto II cytometer with the FACSDiva software (BD Biosciences, San Jose, CA, USA). Relative percentages of cells in respective cell cycle stages were estimated using the ModFit LT 4.1.7 software (Verity Software House, USA).

### 4.12. Tissue samples and Immunohistochemistry

For immunohistochemical (IHC) assay tissue sections (4-μm) were dewaxed, deparaffinized, rehydrated and subjected to the heat antigen retrieval (water bath 95 °C in 10 mM Tris-EDTA buffer, pH 9.0 20 min). After endogenous peroxidase activity quenching (3% H_2_O_2_ 15 min), sections were incubated with anti-human PROX1 antibody (1:1000, AF2727, R&D Systems, Minneapolis, MN, USA) overnight at 4 °C in a humid chamber, followed by incubation with LSAB+ detection kit reagents (DAKO A/S, Glostrup, Denmark) for 2× 30 min at room temperature. Between steps, slides were intensively washed in Tris-buffered saline-1% Tween 20. Immunoreaction was visualized with DAB chromogene (DAKO) and nuclei were counterstained with haematoxylin. The sections were mounted on glass slides and examined under a light microscope (Olympus BX41, Olympus, Carlsbad, CA, USA). Two independent observers evaluated the immunohistochemical staining and scored it as negative or positive.

Fresh FTC samples (*n* = 11) and archived formalin-fixed, paraffin-embedded tissues (*n* = 20) from pathologically proved follicular thyroid carcinomas were obtained from patients treated at Clinic of Endocrinological and General Surgery, Chair of Endocrinology, Medical University of Lodz, Poland. Fresh tissue samples were immediately frozen in −80°C until further processing analysed. The local ethical committees approved (approval number: RNN/135/KE; approval date: 15 July 2014) the study and all patients gave their informed consent.

### 4.13. Statistical Analysis

All experiments were performed at least three times. Data are reported as means with standard deviation (SD). The data were analysed using the nonparametric Mann-Whitney U test (GraphPad, Prism 6.00 for Windows, GraphPad Software, San Diego, CA, USA) and by paired *t*-test where appropriate. *p* values below 0.05 were considered as indicative of a statistical significance.

## Figures and Tables

**Table 1:** List of differentially expressed transcripts in RNA-Seq survey encoding proteins involved in cytoskeleton organization validated in RT-qPCR assay. The significance of differences between control and PROX1 depleted cells was determined with *t*-test based on the results from three biological replicates.

Gene	Refseq ID	RNA-Seq		RT-qPCR		
		*p*-Value	Fold Change	*p*-Value	Fold Change	SD
*TUBB2A*	NM_001069	0.063	0.775	0.001	0.41	0.03
*CALD1*	NM_033138	<0.0001	0.558	<0.0001	0.54	0.08
*ADAM12*	NM_003474	0.001	0.518	0.001	0.57	0.11
*EPS8*	NM_004447	0.022	0.653	0.001	0.62	0.09
*SDC3*	NM_014654	0.012	0.694	0.016	0.63	0.12
*MYO6*	NM_004999	0.012	0.555	0.039	0.65	0.10
*MVP*	NM_005115	0.005	0.666	0.029	0.66	0.09
*PIP5K1C*	NM_012398	0.011	0.696	0.015	0.67	0.08
*MAP4*	NM_002375	0.003	0.650	0.049	0.68	0.19
*SEPT6*	NM_015129	0.053	0.744	0.006	0.68	0.13
*COL6A1*	NM_001848	0.002	0.651	0.000	0.68	0.13
*WASL*	NM_003941	0.010	0.714	0.015	0.74	0.09
*DYNC1H1*	NM_001376	0.001	0.633	0.002	0.76	0.21
*ADAMTS3*	NM_014243	0.011	0.503	0.033	0.77	0.13
*DAG1*	NM_001177639	0.000	0.631	0.005	0.79	0.16
*COL15A1*	NM_001855	0.000	0.274	0.041	0.79	0.15
*DOCK1*	NM_001380	0.017	0.680	0.001	0.79	0.09
*CDC42EP3*	NM_006449	0.048	0.761	0.049	0.81	0.18
*RAPH1*	NM_213589	0.002	0.544	0.002	0.81	0.15
*PFN2*	NM_053024	0.030	0.773	0.017	0.83	0.05
*BRWD3*	NM_153252	0.016	0.588	0.034	0.86	0.04
*FARP1*	NM_005766	0.041	0.726	0.009	0.86	0.08
*ALCAM*	NM_001627	0.001	1.511	0.000	1.24	0.11
*ILK*	NM_004517	0.004	1.515	0.044	1.24	0.17
*CD63*	NM_001780	0.007	1.369	0.040	1.25	0.16
*NPM1*	NM_002520	0.001	1.520	0.001	1.54	0.12
*NUP50*	NM_007172	0.004	1.420	0.017	1.55	0.20
*CLDN12*	NM_012129	0.038	1.589	0.005	1.65	0.26
*EIF6*	NM_181468	0.000	1.749	0.024	1.65	0.20
*FRMD5*	NM_032892	0.008	1.538	0.000	1.81	0.20

SD—standard deviation.

**Table 2:** List of differentially expressed transcripts in RNA-Seq survey encoding proteins involved in cell migration organization and adhesion validated in RT-qPCR assay. The significance of differences between control and *PROX1* depleted cells was determined with *t*-test based on the results from three biological replicates.

Gene	Refseq ID	RNA-Seq		RT-qPCR		
		*p*-Value	Fold Change	*p*-Value	Fold Change	SD
**MIGRATION**						
*CEMIP*	NM_018689	0.001	0.459	<0.0001	0.37	0.04
*SOX2*	NM_003106	0.000	0.412	0.001	0.45	0.04
*DCLK1*	NM_004734	0.010	0.504	0.020	0.52	0.11
*EPHA2*	NM_004431	0.001	1.696	0.001	0.66	0.09
*PTPN13*	NM_080685	0.008	0.660	0.077	0.66	0.09
*COL18A1*	NM_030582	0.013	0.614	0.001	0.68	0.13
*GATA3*	NM_001002295	0.001	0.555	0.001	0.70	0.07
*PLXNB2*	NM_012401	0.041	0.731	0.001	0.73	0.17
*PLXNA1*	NM_032242	0.022	0.712	0.006	0.73	0.09
*PTPN14*	NM_005401	0.003	0.667	<0.0001	0.74	0.16
*SOX9*	NM_000346	0.002	0.623	0.002	0.77	0.12
*NRP2*	NM_201266	0.046	0.752	0.002	0.81	0.12
*PIK3CA*	NM_006218	0.015	0.678	0.022	0.81	0.10
*ARHGAP18*	NM_033515	0.012	1.463	0.002	1.30	0.11
*HAS2*	NM_005328	0.000	2.097	<0.0001	2.13	0.13
**FOCAL ADHESION**						
*TRIOBP*	NM_001039141	0.002	0.623	<0.0001	0.40	0.10
*POSTN*	NM_006475	0.030	0.566	<0.0001	0.44	0.09
*ITGA11*	NM_001004439	0.000	0.456	<0.0001	0.49	0.10
*SVIL*	NM_021738	0.001	0.513	0.008	0.55	0.11
*SYNE2*	NM_182914	0.046	0.672	0.006	0.58	0.10
*PEAK1*	NM_024776	0.000	0.492	0.006	0.68	0.10
*ITGA6*	NM_001079818	0.021	0.723	0.030	0.73	0.07
*ROCK1*	NM_005406	0.001	0.585	0.002	0.80	0.23
*PTK2*	NM_005607	0.013	1.350	0.004	1.24	0.18
*DAPK3*	NM_001348	0.002	1.731	0.024	1.34	0.20
*MCAM*	NM_006500	0.003	1.517	0.023	1.44	0.19
*ITGA2*	NM_002203	0.003	1.403	<0.0001	1.85	0.25
*CAV2*	NM_001233	0.000	1.706	<0.0001	1.88	0.41

SD—standard deviation.

## Supplementary Materials

Supplementary materials can be found at https://www.mdpi.com/1422-0067/20/9/2212/s1.

## Abbreviations

ACTB	beta-actin gene
AKT	protein kinase B
BSA	bovine serum albumin
CAV	caveolin
ChIP-seq	chromatin immunoprecipitation genome wide
DAPI	4′,6-Diamidino-2-Phenylindole
DCLK1	doublecortin like kinase 1
DMEM	Dulbecco’s modified Eagle’s medium
EPHA2	ephrin type-A receptor 2
ERM	ezrin, radixin and moesin
FAK	focal adhesion kinase
FBS	foetal bovine serum
FITC	fluorescein isothiocyanate
FTC	follicular thyroid carcinoma
GO	gene ontology
H&E	haematoxylin and eosin
HRP	horseradish peroxidase
LEC	lymphatic endothelial cells
LYVE-	lymphatic vessel endothelial hyaluronan receptor 1
PBS	phosphate buffered saline
PCA	principal component analysis
PEAK	pseudopodium enriched atypical kinase 1
PIP5KIC	phosphatidylinositol-4-phosphate 5-kinase type 1 gamma
PROX1	Prospero homeobox protein 1
PTC	papillary thyroid carcinoma
qPCR	quantitative polymerase chain reaction
ROCK	the Rho associated coiled-coil containing protein kinase 1
RT-PCR	real-time polymerase chain reaction
SD	standard deviation
SRC	Proto-oncogene tyrosine-protein kinase
SYNE	nesprin
VEGFR	vascular endothelial growth factor receptor
