# Supplementary material for: Molecular Signature of Prospero Homeobox 1 (PROX1) in Follicular Thyroid Carcinoma Cells
Source: Int J Mol Sci. 2019 May 5;20(9):2212. doi: 10.3390/ijms20092212 (PMC6539481; doi:10.3390/ijms20092212)
Supplement: Supplementary file 1 [file ijms-20-02212-s001.pdf]

Supplementary Materials:

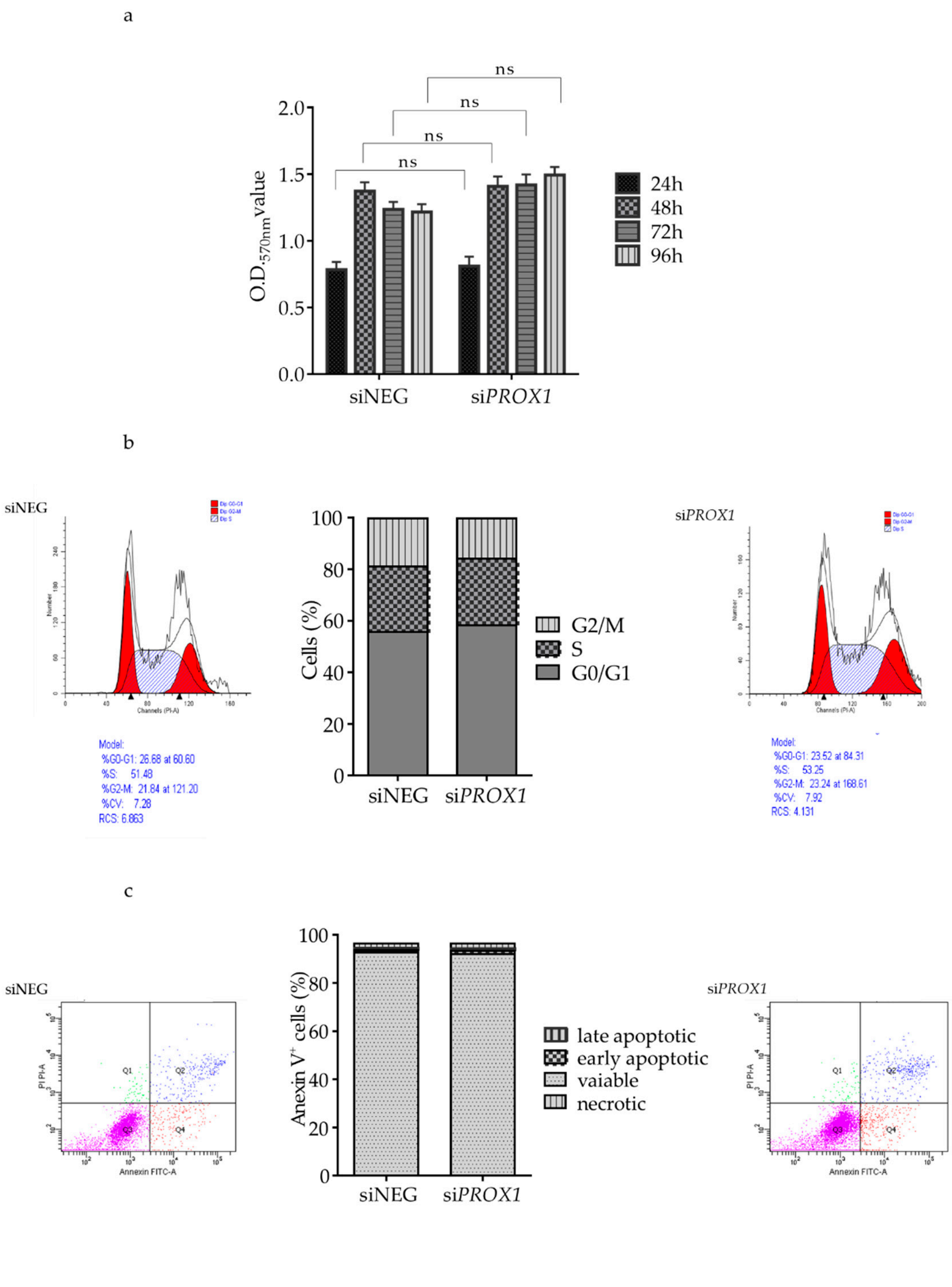

Figure S1. The effect of *PROX1* silencing on the cell cycle, the proliferation and the survival of CGTH-W-1 cells. (**a**) CGTH cell proliferation 24, 48, 72 and 96 h following transfection with *PROX1* siRNA measured by MTT assay. The proliferation of *PROX1*-depleted cells as measured by MTT assay did not differ when compared to the proliferation of control cells transfected with siNEG. Observed small differences were not statistically significant. (**b**) Effect of *PROX1* knock-down on cell cycle and (**c**) apoptosis in CGTH cells. The cell cycle distribution and apoptosis in CGTH cells with silenced *PROX1* and in control cells was measured by flow cytometry 48 h after transfection. The MTT assay was performed in five replicates for each time point. Error bars represent means with standard deviations (SD). A570: absorbance at 570 nm.

Table S1. The primers nucleotide sequences.

prospero homeobox 1 (*PROX1*)	5′–CCAGCTCCAATATGCTGAAGACCTA-3′<br>5′–CATCGTTGATGGCTTGACGTG–3′
beta actin (*ACTB*)	5′–FGCCGAGGACTTTGATTGC–3′<br>5′–CTGTGTGGACTTGGGAGAG–3′
caldesmon 1 (*CALD1*)	5′–GAGCGTCGCAGAGAACTTAGA–3′<br>5′–TCCTCTGGTAGGCGATTCTTT–3′
integrin subunit alpha 2 (*ITGA2*)	5′–GCAAACCTCTGCAAACCCAG–3′<br>5′–CGGTTCTCAGGAAAGCCACT–3′
periostin (*POSTN*)	5′–CTGCTTCAGGGAGACACACC–3′<br>5′–CACTGAGAACGACCTTCCCTT–3′
hyaluronan synthase 2 (*HAS2*)	5′–TGACAGGCATCTCACGAACC–3′<br>5′–CAGCCATTCTCGGAAGTAGG–3′
integrin alpha 11 (*ITGA11*)	5′–GTTGTGCAGTATGGCGAAGA–3′<br>5′–TTCTGGAAAGCCTCTGAGCG–3′
caveolin-2 (*CAV2*)	5′–ACGGAGAAGGCGGACGTA–3′<br>5′–AAAGGAGTGCGTAGTCACCG–3′
cell migration inducing protein, hyaluronan binding (*CEMIP*)	5′–CCAGGAATGTTGAATGTCT–3′<br>5′–ATTGGCTCTTGGTGAATG–3′
TRIO and F-actin binding protein (*TRIOBP*)	5′–CCAGCCAAGGTCTGATGATG–3′<br>5′–TGGCTGACCGTCTATGTGAG–3′
tyrosine phosphatase, non-receptor type 14 (*PTPN14*)	5′–AGCCGAATCCGTGAAGTTGT–3′<br>5′–GTGGCTTTTGGTTCGTCCAC–3′
FERM domain containing 5 (*FRMD5*)	5′–CAGCATTGAGGAGGAGAAGG–3′<br>5′–TCCACCTGGCTAGTTTTTGG–3′
collagen, type XVIII, alpha 1 (*COL18A1*)	5′–AAGGACGAGCTGCTGTTTCC–3′<br>5′–TTGCCGTCAAAGGAGAAGATG–3′
activated leukocyte cell adhesion molecule (*ALCAM*)	5′–TGGACAATTACTGGCAGTGG–3′

	5′–TATCTCGTCTGCCTCATCGTG–3′
dedicator of cytokinesis 1 (*DOCK1*)	5′–TGTGTACCACAAGAGCTGGTT-3′<br>5′–TGTCCACCAAAATTTTCAGGACTTT-3′
SRY (sex determining region Y)-box 2 (*SOX2*)	5′–AACCAGCGCATGGACAGTTA–3′<br>5′–GACTTGACCACCGAACCCAT–3′
EPH receptor A2 (*EPHA2*)	5′–CCATTAAGGACTCGGGGCAG–3′<br>5′–TTGCCATACGGGTGTGTGAG–3′
nucleophosmin (*NPM1*)	5′–AGGAGTGGGGTTGAAAAGCG–3′<br>5′–AGAACGCTGCTCCAGAGAAC–3′
plexin B2 (*PLXNB2*)	5′–GCAGAAACTGAGCTGGACCAC–3′<br>5′–TTCTCGCTGCGGAAGAAGTC–3′
tubulin beta 2A (*TUBB2A*)	5′–GGACGAGATGGAGTTCACCG–3′<br>5′–ACCATGCTTGAGGACAACAGA–3′
epidermal growth factor receptor pathway substrate 8 (*EPS8*)	5′–GCCAACTTCTAATCGCCATA–3′<br>5′–TCACTGTTGTTCCTTGCTAC–3′
GATA binding protein 3 (*GATA3*)	5′–GCCGTTGAGGGTTTCAGAGA–3′<br>5′–TCCGAGCACAACCACCTTAG–3′
sapiens Ras association (RalGDS/AF-6) and pleckstrin homology domains 1 (*RAPH1*)	5′–TCTTTGAGTATGGATGAGGCTG–3′<br>5′–GTGATGCTGGAATGGGAGG–3′
Rho-associated, coiled-coil containing protein kinase 1 (*ROCK1*)	5′–AGGAAGGCGGACATATTAGTCCCT–3′<br>5′–AGACGATAGTTGGGTCCCGGC–3′
dynein, cytoplasmic 1, heavy chain 1 (*DYNC1H1*)	5′–GCCACCGTCAGTTTTGACAC–3′<br>5′–AAATTGCCTCCACCAAACGC–3′
SRY-box 9 (*SOX9*)	5′–AGGAAGTCGGTGAAGAACGG–3′<br>5′–AAGTCGATAGGGGGCTGTCT–3′
neuropilin 2 (*NRP2*)	5′–TCGGCTTTTGCAGGTGAGAA–3′<br>5′–TTTCTTTGTCGGTCGAGGGG–3′
protein tyrosine kinase 2 (*PTK2*)	5′–GGTGCAATGGAGCGAGTATT–3′

	5′–GCCAGTGAACCTCCTCTGA–3′
ADAM metallopeptidase with thrombospondin type 1 motif 3 (*ADAMTS3*)	5′–CGACTCGTGATGGTTCTCCT–3′<br>5′–TGCTCAGGGTTGGAAGACAC–3′
dystroglycan 1 (*DAG1*)	5′–GCAGGGACTGGGAGAACCA–3′<br>5′–ACAGCCTCGTGAAGGTCTGAA–3′
claudin 12 (*CLND12*)	5′–TCAAAGCATGAAGAAAACGAGGC-3′<br>5′–GCTACTGAGGCGATTCCACA–3′
spectrin repeat containing, nuclear envelope 2 (*SYNE2*)	5′–GTGGTCTCTGTCAACGTGAGC–3′<br>5′–GAGCGACTGTCGTAAGCCC-3′
pseudopodium-enriched atypical kinase 1 (*PEAK1*)	5′–TGCTGCACCTACCCAACCCC–3′<br>5′–GGGAGTCTGTAGTGGCAAAAGCCA–3′
plexin A1 (*PLXNA1*)	5′–CACCATGATGCTCACCCCA–3′<br>5′–ATACACCTCGCCTGTCTGCT–3′
septin 6 (*SEPT6*)	5′–AATGGAACCATGAACGCCCA-3′<br>5′–GCTTCACAAAGTCGCAGTGG-3′
supervillin (*SVIL*)	5′–TGGTGTTTGATTTTGGTAGTGAA–3′<br>5′–TAAGCGGATTGCATTCTCCA–3′
FERM RhoGEF (*ARHGEF*) and pleckstrin domain protein 1 (chondrocyte-derived) (*FARP1*)	5′–GACTTCAGGGACTGCCGAG–3′<br>5′–GGACGTCGTTGAACAGGAAG-3′
Wiskott-Aldrich syndrome-like (*WASL*)	5′–CCCTCTTCACTTTCCTCGGC–3′<br>5′–CCCACAATAGTTTCCCATCC-3′
phosphatidylinositol-4-phosphate 5-kinase type 1 gamma (*PIP5K1C*)	5′–CACACAGTCGTCTGGACAGG–3′<br>5′–AAAGTAGATGTCGGTGGCGG–3′
syndecan 3 (*SDC3*)	5′–AGCGTCACATACCAGAAGCC–3′<br>5′–GGCAGACCTTGGGAGAGAG–3′
nucleoporin 50 (*NUP50*)	5′–CGGAAGGAGGTGCTACTGC–3′<br>5′–ACTCGAATCTACCCCCAAAAC–3′
profilin 2 (*PFN2*)	5′–GACTGCACAATGGACATCCG–3′

	5′–TTTTGCCATTGAGTATGCCTTC–3′
doublecortin like kinase 1 (*DCLK1*)	5′–AAGTCCATCATGTCCTTCGGC–3′<br>5′–CTGGGGAGATGGCATACACA–3′
phosphatidylinositol-4,5-bisphosphate 3-kinase catalytic subunit alpha (*PIK3CA*)	5′–GGGACCCGATGCGGTTAG–3′<br>5′–AAGTGGATGCCCCACAGTTC–3′
melanoma cell adhesion molecule (*MCAM*)	5′–CCCTCACACCAGACTCCAAC–3′<br>5′–TGATCTCCTGCTTCCCTGAG –3′
death associated protein kinase 3 (*DAPK3*)	5′–AATCTGAGGAGCTGGGTTGC–3′<br>5′–TGAACTTGGCTGCGTACTCC–3′
eukaryotic translation initiation factor 6 (*EIF6*)	5′–ACTGTGAGATCGGCTGCTTT–3′<br>5′–TCCTGGTCGGTGGTATTGTTG–3′
collagen type VI alpha 1 chain (*COL6A1*)	5′–ATTGCCAAGGACTTCGTCGT–3′<br>5′–TCCACTGCAGGCTCTTGATG–3′
major vault protein (*MVP*)	5′–CAGGATGTGTATGTGCTGTCGG–3′<br>5′–GCTGGAGGCTCTTAGCTGTGTC–3′
integrin alpha 6 (*ITGA6*)	5′–ATGCACGCGGATCGAGTTT–3′<br>5′–TTCCTGCTTCGTATTAACATGCT–3′
ADAM metallopeptidase domain 12 (*ADAM12*)	5′–AAATCCCATGACAATGCGCAG–3′<br>5′–CATGACAATTCCCCCAGACTGG–3′
bromodomain and WD repeat domain containing 3 (*BRWD3*)	5′–CTACCCAGATCGAAGCCGAG–3′<br>5′–GGAATGTGTGCATTTGCTGC–3′
myosin VI (*MYO6*)	5′–TGGGTTTGGACCAAGATGAT–3′<br>5′–CATCACGAGCATTGTTTGCT–3′
CD63 molecule (*CD63*)	5′–GTTACCGCGTCACATGAGGG–3′<br>5′–GTTACCGCGTCACATGAGGG-3′
collagen, type XV, alpha 1 (*COL15A1*)	5′–CCTGCTCTGCATTTGGCTGCTCTG–3′<br>5′–AACAGTCCTGCAGCTCTGGCCTG–3′
integrin linked kinase (*ILK*)	5′–ATCACTCCACAGTCCTCAGGC–3′

	5′–CGGCACTGAGTGAAAATGTCG–3′
CDC42 effector protein (Rho GTPase binding) 3 (*CDC42EP3*)	5′–TTTGACCCCATCTCATTTCG–3′<br>5′–GGTCTTGGCTGGCATTTTGG–3′
microtubule-associated protein 4 (*MAP4*)	5′–ACGTCTCCTACCTCCTCACG–3′<br>5′–TGCCTCTAGTGTGGCAATGA–3′
tyrosine phosphatase, non-receptor type 13 (APO-1/CD95 (Fas)-associated phosphatase) (*PTPN13*)	5′–TTGGAATGACACTGTATTGGGG–3′<br>5′–CCAAGCAGTATGCTGTTGAGAT–3′
Rho GTPase activating protein 18 (*ARHGAP18*)	5′–ACTAACAGCCTACCACCCCA–3′<br>5′–ACTGGCCATATCTGCGACTC–3′

Table S2. Antibodies used for protein detection in PROX-1 deficient CGTH-W-1 cells in Western blot (WB) and immunofluorescent (IF) analyses.

**Used antibodies**	**WB dilution**	**IF dilution**
**Primary antibodies**		
Human Prox1 Antibody (Polyclonal Goat IgG) (cat no AF2727, R&D Systems, USA)	1:2000	1:500
Human Caveolin-2 Antibody (cat no ab75865, Abcam, USA)	1:500	1:500
Human Caveolin-2 (Tyr19) Antibody (cat no, ab3417Abcam, USA)	1:500	1:500
Human FAK Antibody (cat no #3285, Cell Signaling, USA)	1:1000	1:500
Phospho FAK (Tyr397) Antibody (cat no #3283b, Cell Signaling, USA)	1:1000	1:500
Phospho-Src Family (Tyr416) Antibody (cat no #2101 Abcam, USA)	1:1000	n/a
Anti-Ezrin / Radixin / Moesin antibody (cat no ab118572, Abcam, USA)	1:1000	1:500
Anti-Ezrin (phospho Thr567)/ Radixin (phospho Thr564)/ Moesin (phospho Thr558) antibody (cat no ab76247, Abcam, USA)	1:1000	1:500
Nesprin 2 (cat no PA5-62155, ThermoFisher Scientific, USA)	1:1000	1:500
Integrin alpha 2/CD49b Antibody (2B6) (cat no H00003673-M01, Novus Biologicals, USA)	1:1000	n/a

Human EphA2 Antibody (cat no AF3035, R&D Systems, USA)	1:2000	n/a
Integrin alpha 11 Antibody (cat no AF4235, Novus Biologicals, USA)	1:1000	n/a
Anti-β-actin (cat no A2228 Sigma-Aldrich, USA)	1:5000	n/a
**Secondary antibodies**		
HRP Rabbit Anti-Goat IgG (cat no 305-035-046, JacksonImmunoResearch, USA)	1:20000	n/a
HRP Goat Anti-Rabbit Immunoglobulins (cat no P0448, DAKO, Denmark)	1:5000	n/a
Rabbit F(ab')2 polyclonal Secondary Antibody to Goat IgG-H&L DyLight® 594 (cat no ab96885, Abcam, United Kingdom)	n/a	1:500
Goat anti-Rabbit IgG (H+L) Secondary Antibody, Rhodamine Red (cat no 111-025-144, Jackson ImmunoResearch Laboratories, USA)	n/a	1:100

n/a: analysis not performed.

Table S3. The list of genes with number of reads in a given biological replicate (I, II, III) for cells treated with lipofectamine with control siRNA (siNEG) and lipofectamine alone (Lipo). The fold change (FC) and adj. P. values (p.adj) are provided.

**Gene**	**refSeq**	**Lipo I**	**Lipo II**	**Lipo III**	**siNEG I**	**siNEG II**	**siNEG III**	**log2FoldChange**	**p.adj**	**FC**
PLAT	NM_000930	52	50	88	284	263	291	-1.066822979	4.64E-06	0.48
GABARAPL1	NM_031412	100	93	83	383	316	438	-1.021792085	1.34E-06	0.49
NUPR1	NM_001042483	340	281	377	1208	1160	1447	-0.989195929	6.66E-08	0.50
NKD2	NM_033120	15	18	25	69	83	125	-0.923903394	0.00345	0.53
LPAR1	NM_001401	128	183	208	577	591	716	-0.920146757	3.31E-06	0.53
SEPT3	NM_145733	115	116	98	415	352	445	-0.905518146	1.68E-05	0.53
NEK11	NM_024800	19	20	15	71	77	103	-0.901080038	0.00465	0.54
AKAP12	NM_005100	48	55	62	196	202	227	-0.90102719	0.00021	0.54
COL15A1	NM_001855	41	55	53	198	166	205	-0.896496725	0.00035	0.54
ARHGDIB	NM_001175	79	76	82	274	255	326	-0.867928859	0.00013	0.55
LGALS3BP	NM_005567	100	125	149	444	323	499	-0.802534722	0.00041	0.57
SNCA	NM_000345	21	20	19	81	74	88	-0.800368935	0.01522	0.57
SLC43A2	NM_152346	23	16	34	112	78	95	-0.764425239	0.02322	0.59
SVIL	NM_021738	38	50	37	164	124	152	-0.763260478	0.01022	0.59
TUBB2B	NM_178012	159	182	175	501	530	602	-0.754837792	0.00018	0.59
ANO1	NM_018043	130	84	102	397	297	372	-0.748731647	0.00513	0.60
RALBP1	NM_006788	93	66	54	325	282	173	-0.747429956	0.02179	0.60
KIAA1211	NM_020722	71	46	65	203	183	233	-0.741523585	0.00922	0.60
STAT2	NM_005419	44	43	46	154	153	143	-0.734565468	0.0123	0.60
SLC16A14	NM_152527	29	21	18	85	73	100	-0.725621586	0.03684	0.60

POU3F4	NM_000307	30	15	18	96	58	98	-0.717383051	0.0489	0.61
NREP	NM_00114247 6	92	132	117	323	360	381	-0.716553226	0.00279	0.61
NPHP3	NM_153240	18	25	18	76	59	95	-0.711471163	0.0462	0.61
CLEC2B	NM_005127	22	26	29	93	78	101	-0.707206834	0.03623	0.61
ZNF362	NM_152493	123	109	131	400	423	321	-0.704944667	0.00863	0.61
STK32B	NM_018401	36	67	48	136	174	188	-0.69781426	0.02331	0.62
ASNS	NM_001673	332	193	234	828	730	863	-0.686600008	0.01195	0.62
SORT1	NM_002959	62	70	61	171	217	223	-0.684436521	0.01581	0.62
RNF144A	NM_014746	53	53	38	188	147	141	-0.683267919	0.03214	0.62
SPON2	NM_012445	64	66	80	212	206	233	-0.680478974	0.01111	0.62
C1QTNF6	NM_031910	37	60	48	154	122	193	-0.674123552	0.02843	0.63
SLC36A1	NM_078483	140	70	93	441	339	247	-0.672511057	0.0489	0.63
MXD4	NM_006454	53	54	67	206	152	189	-0.670966936	0.02052	0.63
ZEB2	NM_014795	66	73	70	245	218	187	-0.669651874	0.01985	0.63
CADM1	NM_014333	241	379	433	1105	848	1154	-0.660432033	0.00487	0.63
CRISPLD1	NM_031461	53	64	84	208	178	231	-0.658411235	0.01926	0.63
DNASE2	NM_001375	140	162	210	494	484	525	-0.657765228	0.00423	0.63
FAM129A	NM_052966	150	164	187	446	451	572	-0.652533885	0.00332	0.64
DYNC2H1	NM_00108046 3	53	73	53	169	162	223	-0.647298727	0.02812	0.64
TNFRSF21	NM_014452	176	239	265	669	554	742	-0.641639622	0.00345	0.64
ACP6	NM_016361	126	111	127	324	344	406	-0.638175786	0.0106	0.64
PEAK1	NM_024776	114	116	87	308	303	335	-0.636112139	0.01664	0.64
SPRED2	NM_181784	178	195	189	586	500	534	-0.63582467	0.00454	0.64
CLIP3	NM_015526	110	120	106	365	294	330	-0.635611855	0.012	0.64
TIPARP	NM_00118471 8	140	181	175	532	524	387	-0.627106864	0.02082	0.65
IL13RA1	NM_001560	80	81	75	243	211	245	-0.626165986	0.02102	0.65
TUBA1A	NM_006009	2721	2898	3917	8375	8006	10082	-0.606568814	0.00203	0.66
EMP1	NM_001423	2930	2995	3802	9099	8541	9138	-0.605105324	0.0011	0.66
TNPO2	NM_00113619 6	561	504	646	1651	1493	1592	-0.595544086	0.00465	0.66
LDB1	NM_00111340 7	153	175	235	579	494	504	-0.594497906	0.01803	0.66
TPRG1L	NM_182752	163	164	173	439	439	505	-0.580204894	0.012	0.67
ATP2A2	NM_170665	969	702	630	2482	2057	2067	-0.579971735	0.04033	0.67
SNIP1	NM_024700	115	152	171	418	337	457	-0.573558556	0.02179	0.67
ZC3H7A	NM_014153	126	134	111	333	319	376	-0.563059664	0.03097	0.68
ADAM19	NM_033274	1784	1300	1782	5027	4108	4261	-0.56274257	0.02331	0.68
PPP1R15A	NM_014330	161	168	275	503	504	657	-0.555416743	0.04623	0.68
HMOX1	NM_002133	130	124	185	404	373	423	-0.553921086	0.03725	0.68
USP10	NM_005153	202	226	229	516	534	709	-0.538471518	0.02136	0.69
SLC7A11	NM_014331	393	398	396	1091	964	1072	-0.533071771	0.012	0.69
ITPRIPL2	NM_00103484 1	173	220	209	479	511	606	-0.532870193	0.02331	0.69
SSH1	NM_018984	284	438	308	775	852	1129	-0.526420087	0.04824	0.69
TM9SF2	NM_004800	351	317	358	932	824	945	-0.524894517	0.01908	0.70
CABIN1	NM_012295	161	167	162	398	362	561	-0.524056877	0.04917	0.70

HN1	NM_001002032	626	646	844	1948	1665	1859	-0.514815951	0.01848	0.70
TRIB2	NM_021643	602	817	986	2140	1862	2208	-0.514649284	0.02277	0.70
ALDH1L2	NM_001034173	191	218	194	509	440	637	-0.508642072	0.04123	0.70
CASP4	NM_001225	381	439	371	991	899	1203	-0.506073362	0.02231	0.70
CCND1	NM_053056	4420	7771	7765	16341	15019	20145	-0.503145861	0.04824	0.71
TCAF1	NM_014719	218	233	216	536	537	657	-0.500756504	0.03637	0.71
FAM134A	NM_024293	201	202	219	538	464	597	-0.489566782	0.04377	0.71
CALM2	NM_001743	1353	1562	1776	3913	3374	4162	-0.448482011	0.02331	0.73
C11orf84	NM_138471	548	592	680	1450	1252	1754	-0.438357954	0.04958	0.74
PSAT1	NM_058179	1133	1189	1153	2968	2443	3015	-0.429791634	0.04472	0.74
HSPA5	NM_005347	2908	3734	3341	4336	3703	4702	0.4104203	0.0489	1.33
CYCS	NM_018947	1672	2209	1892	2249	2258	2623	0.447451367	0.03328	1.36
MBOAT7	NM_024298	772	891	1128	1106	1066	1234	0.452167691	0.0489	1.37
PSMB2	NM_002794	3940	5518	5562	5833	5345	7261	0.453502808	0.03515	1.37
ATP5L	NM_006476	3014	3658	3531	4077	3821	4729	0.454490803	0.01502	1.37
RBX1	NM_014248	1200	1335	1485	1612	1594	1712	0.458966205	0.02216	1.37
ARL4C	NM_005737	458	519	496	522	591	658	0.47483912	0.04033	1.39
HIST1H2AJ	NM_021066	1420	2169	1818	1947	1923	2617	0.475481249	0.04458	1.39
DNER	NM_139072	392	514	496	573	498	613	0.476009276	0.03386	1.39
SNRPA1	NM_003090	923	1159	1256	1313	1246	1429	0.486879645	0.01581	1.40
TMBIM6	NM_001098576	1745	1367	1824	2133	1669	2077	0.487738201	0.04929	1.40
INSIG1	NM_005542	976	1298	1145	1185	1389	1465	0.492884012	0.02537	1.41
GNG5	NM_005274	880	1127	1226	1219	1089	1538	0.494379271	0.02082	1.41
DSN1	NM_024918	356	321	455	452	396	476	0.498561382	0.04946	1.41
ATP6AP2	NM_005765	207	244	259	270	264	295	0.498671749	0.04562	1.41
SEC62	NM_003262	726	1142	1195	1199	1051	1317	0.499327596	0.03725	1.41
MRPL32	NM_031903	717	835	880	1033	828	1024	0.499351722	0.01522	1.41
RFC4	NM_002916	306	364	362	386	392	426	0.508168526	0.02368	1.42
TLN2	NM_015059	289	295	422	374	346	441	0.51000441	0.04859	1.42
PCIF1	NM_022104	234	298	327	320	262	411	0.512532491	0.04859	1.43
AP3D1	NM_003938	480	623	582	661	563	743	0.517956357	0.01458	1.43
DUSP12	NM_007240	222	368	328	339	299	406	0.519987831	0.04928	1.43
PPP6R1	NM_014931	628	918	872	948	816	1029	0.523087116	0.01529	1.44
TYMS	NM_001071	1699	2047	2257	2254	2196	2546	0.524836792	0.0047	1.44
DUSP7	NM_001947	604	1075	903	835	948	1120	0.529756695	0.04115	1.44
POMK	NM_032237	245	259	222	276	256	296	0.53044301	0.03637	1.44
TMEM55A	NM_018710	159	213	198	213	196	235	0.531314181	0.04033	1.45
DHX34	NM_014681	221	272	319	341	268	306	0.531799877	0.03725	1.45
DLC1	NM_182643	354	370	359	412	397	435	0.532035324	0.01748	1.45
WSB2	NM_018639	600	958	941	916	848	1073	0.533530455	0.02082	1.45
ANKRD13A	NM_033121	418	459	446	519	473	529	0.53493096	0.012	1.45
TMX1	NM_030755	626	815	761	827	851	826	0.538116865	0.01198	1.45
CCSAP	NM_145257	180	230	272	268	247	241	0.538545359	0.04472	1.45
MYL12B	NM_033546	1281	1788	1578	1613	1819	1842	0.541678845	0.01075	1.46

MSANTD3	NM_00119880 5	385	386	429	463	428	476	0.543839018	0.01222	1.46
DCTD	NM_00101273 2	511	808	761	737	736	868	0.543921435	0.01694	1.46
LGR4	NM_018490	164	213	159	204	167	225	0.545832533	0.0489	1.46
DUSP1	NM_004417	397	568	494	564	485	592	0.554137571	0.01195	1.47
RBM8A	NM_005105	1867	1623	1640	1771	1922	2124	0.556055474	0.01195	1.47
SAPCD2	NM_178448	115	178	151	155	148	178	0.561385337	0.04754	1.48
GALNT1	NM_020474	186	254	249	236	224	302	0.562617356	0.02216	1.48
TTL	NM_153712	234	316	275	284	308	313	0.569035904	0.01773	1.48
MRPL51	NM_016497	573	619	546	621	668	637	0.571007903	0.01075	1.49
DONSON	NM_017613	111	137	108	119	115	147	0.574384579	0.04958	1.49
NKD1	NM_033119	517	875	836	780	620	1026	0.575009236	0.02331	1.49
TWF1	NM_00124239 7	261	500	321	377	340	438	0.576655711	0.04033	1.49
SEMA3C	NM_006379	186	165	177	199	162	212	0.581777505	0.02683	1.50
CDK2AP2	NM_005851	2312	2707	2934	3433	2261	3084	0.584171958	0.0059	1.50
DNM1	NM_004408	133	154	199	185	163	166	0.584518663	0.03511	1.50
DNAJB6	NM_058246	1498	1493	1697	2157	1313	1623	0.586350956	0.01581	1.50
TFPI2	NM_006528	271	511	342	361	352	480	0.587153696	0.03357	1.50
PCGF3	NM_006315	158	219	229	185	225	234	0.588380739	0.02368	1.50
BAG4	NM_004874	262	581	397	375	402	508	0.592694031	0.04472	1.51
CCDC50	NM_178335	300	454	325	388	349	424	0.594424779	0.01433	1.51
TSPAN13	NM_014399	246	341	291	294	268	388	0.596271348	0.012	1.51
FSTL3	NM_005860	143	115	190	144	156	164	0.597507111	0.04472	1.51
NAA30	NM_00101171 3	182	256	172	214	206	223	0.598874659	0.02527	1.51
GREM1	NM_013372	465	1047	794	753	748	880	0.602190219	0.03038	1.52
BNC1	NM_001717	129	175	157	145	165	171	0.607949562	0.02182	1.52
NXPE3	NM_145037	139	294	204	187	184	274	0.612911764	0.0447	1.53
CD68	NM_001251	791	979	844	1026	796	1007	0.615873619	0.00194	1.53
HIST1H3J	NM_003535	101	109	164	124	107	149	0.617170601	0.03725	1.53
MAPKAPK2	NM_032960	282	319	279	302	279	360	0.620473654	0.0052	1.54
CPOX	NM_000097	90	95	79	83	73	110	0.621418323	0.04822	1.54
NUP50	NM_007172	759	1259	943	958	901	1274	0.621540282	0.00741	1.54
AREL1	NM_00103947 9	423	485	618	530	546	529	0.622791718	0.00498	1.54
FLNC	NM_001458	2402	3143	3685	3246	2893	3762	0.623212811	0.0011	1.54
LOXL1	NM_005576	153	132	175	193	119	156	0.623677512	0.0326	1.54
EIF4EBP2	NM_004096	269	519	355	395	369	392	0.635385931	0.01604	1.55
INPP5A	NM_005539	91	182	143	107	146	149	0.639493528	0.04025	1.56
PLOD2	NM_182943	604	1017	572	640	675	911	0.64303359	0.01604	1.56
HAS2	NM_005328	666	888	762	726	769	948	0.645140892	0.00105	1.56
SIRPA	NM_080792	105	163	109	134	106	133	0.645655298	0.02537	1.56
HDAC4	NM_006037	145	176	176	182	145	180	0.65012615	0.00903	1.57
AGPAT9	NM_032717	91	171	149	128	132	141	0.650371156	0.02331	1.57
TENM2	NM_00112267 9	466	836	575	539	556	809	0.650909561	0.01146	1.57
RASA4	NM_006989	530	539	719	590	571	707	0.651473937	0.00149	1.57

VASN	NM_138440	160	217	269	226	205	217	0.652644162	0.0106	1.57
SSX2IP	NM_00116629 3	150	178	125	131	134	189	0.652723324	0.01958	1.57
IMPAD1	NM_017813	520	1193	826	816	798	861	0.662947581	0.01462	1.58
ABCB10	NM_012089	62	124	94	84	87	90	0.665497724	0.0355	1.59
WWC1	NM_00116166 1	215	279	337	269	259	312	0.667794255	0.00345	1.59
PALLD	NM_00116610 8	71	102	106	85	76	106	0.673309022	0.02147	1.59
AKIRIN1	NM_024595	315	600	443	409	352	577	0.677205342	0.01021	1.60
BACE1	NM_012104	323	543	612	429	462	561	0.681458429	0.00528	1.60
MTSS1L	NM_138383	296	470	422	385	383	418	0.684297401	0.00193	1.61
DCUN1D5	NM_032299	457	629	433	507	511	484	0.689987729	0.00306	1.61
PDGFA	NM_002607	1707	3950	2853	2510	2635	2994	0.691013583	0.0088	1.61
PCDHGC5	NM_018929	103	139	159	124	108	155	0.691220898	0.01021	1.61
WDR62	NM_00108396 1	191	182	213	203	158	220	0.694886767	0.00345	1.62
ADAMTS14	NM_139155	67	68	56	67	55	45	0.697560707	0.03725	1.62
TMEM64	NM_00100849 5	96	164	96	101	102	126	0.701193319	0.01803	1.63
TNFRSF19	NM_018647	215	345	241	202	250	318	0.701728881	0.00729	1.63
FBN1	NM_000138	1839	2689	2103	2352	2111	2139	0.703160267	0.00035	1.63
SETD7	NM_030648	227	358	251	259	255	295	0.710619456	0.00302	1.64
PDHX	NM_003477	201	171	186	173	156	210	0.722304201	0.0031	1.65
PLAGL1	NM_00108095 4	70	122	96	67	91	97	0.726856732	0.01581	1.66
ZC3HAV1L	NM_080660	62	124	64	60	74	77	0.727565965	0.02542	1.66
VPS33A	NM_022916	99	89	91	71	87	96	0.728302176	0.012	1.66
LFNG	NM_00104016 7	93	101	84	110	53	81	0.730269349	0.01958	1.66
COL8A1	NM_020351	369	365	511	405	391	404	0.731356882	0.00058	1.66
SUV39H1	NM_003173	112	138	154	127	114	137	0.736143382	0.00332	1.67
RAB3B	NM_002867	123	239	155	154	123	192	0.737955576	0.00903	1.67
CACNA2D3	NM_018398	98	99	109	98	90	91	0.739730974	0.00672	1.67
NBN	NM_002485	264	438	344	249	326	399	0.74289834	0.00279	1.67
TWIST1	NM_000474	898	1216	1080	1043	900	1189	0.747560647	1.47E-05	1.68
LCLAT1	NM_00100225 7	204	371	235	238	190	305	0.764284717	0.00345	1.70
EIF5A2	NM_020390	87	151	127	88	118	106	0.778548471	0.00549	1.72
LAYN	NM_178834	106	141	138	101	121	119	0.78136486	0.00228	1.72
CA13	NM_198584	33	60	58	34	36	45	0.792519686	0.01587	1.73
INIP	NM_021218	206	216	207	201	179	196	0.792786456	0.00024	1.73
IGIP	NM_00100718 9	41	50	44	29	29	45	0.795114107	0.01604	1.74
RLTPR	NM_00101383 8	70	84	72	80	51	51	0.799627889	0.00937	1.74
IRS1	NM_005544	308	460	392	366	303	394	0.803345441	6.00E-05	1.75
CACNA2D1	NM_000722	144	185	164	148	125	168	0.807545325	0.00038	1.75
KLF16	NM_031918	67	64	80	62	56	53	0.809029848	0.0065	1.75
ALCAM	NM_001627	1013	1302	1050	979	887	1222	0.831786863	2.17E-06	1.78
ELL2	NM_012081	178	239	240	167	168	241	0.837600357	0.00014	1.79

SDC4	NM_002999	281	502	390	293	321	384	0.860609114	8.84E-05	1.82
FGF2	NM_002006	115	131	144	125	92	94	0.893108889	0.00029	1.86
GREM2	NM_022469	60	91	102	59	62	68	0.90667285	0.00094	1.87
GNPNAT1	NM_198066	769	1398	1000	785	789	1037	0.913639509	8.59E-06	1.88
PMAIP1	NM_021127	115	182	166	105	119	139	0.927163397	7.58E-05	1.90
ARSJ	NM_024590	103	94	84	82	64	62	0.928542513	0.00058	1.90
ENPP1	NM_006208	172	358	200	176	159	214	0.940787974	0.00019	1.92
NIPA1	NM_144599	146	172	183	118	123	153	0.95941304	6.55E-06	1.94
EFHD2	NM_024329	1688	1937	2594	1917	1343	1724	0.970830704	2.19E-07	1.96
GADD45A	NM_001924	149	258	288	186	158	157	1.00213665	1.47E-05	2.00
UBE2V2	NM_003350	1426	2111	1458	1247	1238	1418	1.009614125	4.72E-08	2.01
TMTC3	NM_181783	215	208	207	169	136	170	1.02660053	3.81E-07	2.04
CAV2	NM_001233	800	1400	992	746	747	920	1.027547381	1.50E-07	2.04
SLC9A6	NM_001042537	159	213	242	126	130	179	1.075579012	2.76E-07	2.11
MMP1	NM_002421	1231	1928	1938	939	903	1723	1.09564922	2.19E-07	2.14
TAF9B	NM_015975	403	523	388	292	280	370	1.112067054	4.00E-09	2.16
TULP3	NM_001160408	514	683	569	411	420	440	1.121466442	1.06E-10	2.18
FAM46A	NM_017633	131	243	149	89	104	113	1.210305447	2.19E-07	2.31

Table S4. The list of genes with number of reads in a given biological replicate (I, II, III) for cells treated with lipofectamine with control siRNA (siNEG) and lipofectamine with anti-Prox siRNA (siProx). The fold change (FC) and adj. P. values (p.adj) are provided

Gene	refSeq	siNEG I	siNEG II	siNEG III	siProx_sig I	siProx_sig II	siProx_sig III	log2FoldChange	p.adj	FC
PROX1	NM_002763	62	58	103	9	3	3	-1.94842698	3.30E-09	0.26
BCHE	NM_000055	56	27	41	7	1	5	-1.356975	0.00023	0.39
RASSF2	NM_170774	127	100	126	37	5	27	-1.309931997	1.66E-05	0.40
SOX2	NM_003106	53	58	48	10	7	7	-1.279746525	0.00028	0.41
MXRA5	NM_015419	59	63	81	17	6	13	-1.262999511	0.00015	0.42
FTH1	NM_002032	13694	12408	16689	4735	2522	3501	-1.201940321	2.09E-19	0.43
PNMA2	NM_007257	78	95	91	27	12	18	-1.155317074	0.00022	0.45
FBXO32	NM_058229	80	74	59	30	6	7	-1.152255526	0.00099	0.45
AHRR	NM_001242412	344	330	377	102	66	91	-1.14874647	3.38E-08	0.45
LZTS1	NM_021020	155	106	142	43	22	28	-1.146113924	3.62E-05	0.45
ITGA11	NM_001004439	547	517	535	229	84	112	-1.133746031	1.20E-08	0.46
GPR27	NM_018971	61	44	39	12	7	6	-1.128923066	0.00202	0.46
CEMIP	NM_018689	65	70	94	26	11	12	-1.122742787	0.00057	0.46
PEG10	NM_001172438	2026	1959	2502	889	389	491	-1.112049717	6.16E-13	0.46
SAMD9	NM_001193307	113	111	121	44	17	25	-1.070602811	0.00019	0.48
TESK1	NM_006285	94	47	63	20	10	13	-1.065095072	0.0022	0.48
MBD6	NM_052897	119	100	113	55	14	12	-1.064743602	0.001	0.48
FAM167A	NM_053279	243	271	247	79	49	70	-1.061523987	6.00E-06	0.48
ZNF213	NM_004220	41	53	72	20	4	11	-1.048351057	0.00371	0.48
GRIN2A	NM_001134407	59	49	61	17	9	10	-1.047367797	0.00272	0.48
EBF1	NM_024007	68	67	55	20	10	12	-1.045952801	0.00235	0.48
IGFBP7	NM_001553	75	96	57	33	6	14	-1.04446737	0.00289	0.48
CHST15	NM_015892	56	55	49	21	8	4	-1.042551342	0.0045	0.49

FXYD6	NM_001164836	735	837	985	363	168	194	-1.032197849	1.78E-08	0.49
C1R	NM_001733	108	100	137	61	9	20	-1.016790685	0.0017	0.49
PHACTR2	NM_001100164	213	250	322	93	38	84	-1.011755299	2.79E-05	0.50
ICA1	NM_022307	71	67	69	23	13	13	-1.002394661	0.0027	0.50
FBXW7	NM_033632	152	138	245	63	26	53	-1.000609293	0.00023	0.50
PLSCR4	NM_020353	132	111	157	46	26	33	-0.998122633	0.00028	0.50
ADAMTS3	NM_014243	31	42	33	13	3	4	-0.99073792	0.01073	0.50
DCLK1	NM_004734	49	43	37	11	2	13	-0.988289339	0.00961	0.50
PRSS23	NM_007173	504	454	537	206	117	100	-0.986817172	2.87E-06	0.50
ZNF709	NM_152601	55	54	51	28	4	2	-0.986270618	0.01057	0.50
NR3C2	NM_000901	42	37	57	15	5	9	-0.985328139	0.00804	0.51
KLF9	NM_001206	149	157	183	55	31	47	-0.982315438	0.00014	0.51
TP53I11	NM_006034	65	61	64	29	9	8	-0.976174924	0.00576	0.51
AFF3	NM_001025108	76	62	59	30	9	9	-0.975726766	0.00576	0.51
ADAM12	NM_003474	125	101	112	47	20	26	-0.9503423	0.00114	0.52
AHR	NM_001621	259	253	386	113	53	93	-0.950044281	3.04E-05	0.52
SERTAD2	NM_014755	143	199	215	89	29	44	-0.943255221	0.00047	0.52
ZFP36	NM_003407	173	194	188	55	40	59	-0.934992749	0.00038	0.52
VGLL3	NM_016206	192	171	149	104	17	30	-0.932341349	0.00366	0.52
DOCK11	NM_144658	367	233	347	169	52	64	-0.931164215	0.00032	0.52
ZNF627	NM_145295	151	169	95	78	18	22	-0.924882503	0.00606	0.53
SLC1A4	NM_003038	656	604	750	293	125	209	-0.91529234	1.35E-07	0.53
CDYL2	NM_152342	61	64	83	32	4	19	-0.915137485	0.00988	0.53
SLC38A1	NM_001077484	291	257	301	129	48	84	-0.912771813	3.31E-05	0.53
BOC	NM_033254	36	35	45	16	4	6	-0.911294588	0.01867	0.53
TIMP3	NM_000362	14279	12017	17623	5796	3270	4409	-0.907127547	1.91E-09	0.53
SNED1	NM_001080437	159	145	204	74	35	41	-0.899867175	0.00049	0.54
SDC2	NM_002998	215	235	239	74	59	65	-0.898029189	0.00032	0.54
REPIN1	NM_001099695	80	54	53	27	11	10	-0.897277998	0.01284	0.54
ZBED4	NM_014838	149	130	199	78	26	39	-0.895063382	0.00101	0.54
EGR1	NM_001964	83	80	72	31	18	14	-0.893824626	0.00752	0.54
RSBN1	NM_018364	183	148	179	84	21	52	-0.884013249	0.00117	0.54
PCK2	NM_004563	218	225	257	88	59	62	-0.879072801	0.00023	0.54
CHST1	NM_003654	160	121	179	69	34	33	-0.878876404	0.00137	0.54
RAPH1	NM_213589	139	153	183	71	17	56	-0.878475015	0.00213	0.54
CELSR3	NM_001407	194	152	184	82	36	44	-0.873539768	0.00067	0.55
SPTBN2	NM_006946	141	128	158	65	31	32	-0.870981401	0.00146	0.55
PIK3C2A	NM_002645	111	94	146	58	23	22	-0.86745071	0.00448	0.55
TFG	NM_001195478	282	266	368	177	61	47	-0.865904245	0.00197	0.55
ZNF71	NM_021216	45	36	20	10	5	6	-0.86589755	0.0318	0.55
LCA5	NM_001122769	149	128	188	61	32	46	-0.86507229	0.00107	0.55
FAM155A	NM_001080396	197	144	184	87	31	45	-0.864303014	0.00105	0.55
TCTN2	NM_024809	66	64	77	27	16	13	-0.863273734	0.01154	0.55
BCL6	NM_001706	106	49	113	47	8	20	-0.863039932	0.01591	0.55
PLK2	NM_006622	98	101	128	49	23	24	-0.86284093	0.00392	0.55
MESDC1	NM_022566	28	32	62	8	6	12	-0.858588954	0.03053	0.55

PCDH18	NM_019035	318	322	340	198	53	70	-0.856515744	0.00091	0.55
SCD5	NM_001037582	122	123	162	64	20	41	-0.855342394	0.00238	0.55
H6PD	NM_004285	110	124	149	56	23	37	-0.854479675	0.00246	0.55
ICK	NM_014920	170	211	234	82	39	69	-0.853234499	0.00066	0.55
MYO6	NM_004999	64	79	105	41	11	20	-0.849309417	0.01159	0.56
ZBED3	NM_032367	30	29	41	12	6	4	-0.8492693	0.03425	0.56
GATA3	NM_001002295	165	152	186	65	35	54	-0.848855542	0.00088	0.56
EFHD1	NM_025202	37	29	30	9	5	7	-0.847286217	0.03486	0.56
ARRDC3	NM_020801	104	115	132	58	13	37	-0.846208618	0.00566	0.56
NPTX1	NM_002522	88	84	98	32	28	12	-0.843621614	0.01379	0.56
CALD1	NM_033138	1080	914	1101	455	192	373	-0.842073076	1.73E-06	0.56
APOL6	NM_030641	38	25	33	8	4	9	-0.834241303	0.039	0.56
DPP8	NM_130434	187	206	188	108	42	34	-0.83236461	0.00367	0.56
RCOR2	NM_173587	137	148	154	66	34	36	-0.831796736	0.00229	0.56
ULK1	NM_003565	232	205	264	114	52	59	-0.829366913	0.00045	0.56
SKI	NM_003036	896	920	877	502	209	178	-0.82870438	0.0004	0.56
KLHL24	NM_017644	60	64	62	19	8	24	-0.825507501	0.02198	0.56
PBXIP1	NM_020524	131	112	137	64	20	36	-0.824950487	0.00411	0.56
LHFP	NM_005780	191	168	224	102	25	63	-0.824593509	0.00204	0.56
CREBRF	NM_153607	234	221	288	113	53	74	-0.822057071	0.00028	0.57
POSTN	NM_006475	50	41	53	13	8	16	-0.820651548	0.02994	0.57
ZBTB10	NM_001105539	75	45	99	26	15	19	-0.820596197	0.01944	0.57
PIK3R2	NM_005027	554	698	612	372	108	160	-0.818330389	0.00055	0.57
GSTM2	NM_001142368	201	137	200	88	31	54	-0.816359015	0.00206	0.57
NTF3	NM_001102654	100	118	126	52	23	32	-0.812453442	0.00576	0.57
TFAP2C	NM_003222	690	807	989	410	200	217	-0.805896418	4.38E-05	0.57
STOX2	NM_020225	37	30	31	7	7	8	-0.804165051	0.04721	0.57
TXNIP	NM_006472	593	406	478	201	121	156	-0.803027687	0.00022	0.57
TMEM131	NM_015348	221	188	194	140	24	39	-0.800324365	0.01248	0.57
KLF7	NM_003709	218	223	212	140	31	51	-0.798519684	0.00568	0.57
RAB3D	NM_004283	248	253	236	107	55	80	-0.796140875	0.00068	0.58
PTEN	NM_000314	33	43	42	16	4	11	-0.7934996	0.04507	0.58
AKAP13	NM_006738	109	69	88	42	10	30	-0.793047632	0.01883	0.58
OLFM2	NM_058164	140	120	134	47	39	33	-0.791359751	0.00742	0.58
SLC7A8	NM_012244	87	65	98	41	19	15	-0.790168998	0.01908	0.58
FAM160B1	NM_020940	93	108	159	47	22	42	-0.790159854	0.00937	0.58
TRIQK	NM_001171799	143	127	137	63	25	44	-0.788649173	0.00457	0.58
ST6GAL2	NM_032528	260	196	281	88	64	81	-0.78435973	0.00147	0.58
ODF2L	NM_001007022	96	113	112	47	17	38	-0.783965445	0.01053	0.58
SLC1A1	NM_004170	83	101	97	38	23	25	-0.7826926	0.01354	0.58
TCF20	NM_005650	97	71	84	37	19	21	-0.779789565	0.01742	0.58
FOSL1	NM_005438	6092	4516	6229	2639	1383	1717	-0.778572314	1.01E-06	0.58
LMO4	NM_006769	171	202	109	113	16	32	-0.778445245	0.03072	0.58
KIAA1549	NM_001164665	86	88	114	52	16	24	-0.777641665	0.01482	0.58
DDX52	NM_007010	461	341	435	275	69	90	-0.774483034	0.00432	0.58
ROCK1	NM_005406	616	629	882	394	105	248	-0.774345099	0.00066	0.58

CELSR1	NM_014246	192	153	220	77	41	66	-0.772646685	0.00235	0.59
PRDM8	NM_001099403	52	66	63	26	7	21	-0.770732687	0.03538	0.59
PTGER2	NM_000956	79	99	79	31	23	23	-0.770148052	0.02129	0.59
RPS6KA2	NM_001006932	201	218	238	102	40	80	-0.768800642	0.0016	0.59
IRS2	NM_003749	212	281	298	129	61	76	-0.768252156	0.00142	0.59
VLDLR	NM_003383	130	107	139	51	19	51	-0.767208203	0.01021	0.59
BRWD3	NM_153252	118	101	107	63	14	31	-0.76705928	0.01591	0.59
ZMAT3	NM_022470	163	129	196	59	31	66	-0.76490315	0.00653	0.59
SHROOM3	NM_020859	77	73	90	32	21	20	-0.761048554	0.02145	0.59
PYGO1	NM_015617	199	153	181	79	31	68	-0.760837457	0.00434	0.59
PKN3	NM_013355	79	60	52	37	7	15	-0.75929963	0.04322	0.59
PLXND1	NM_015103	91	96	125	45	20	35	-0.757674525	0.01296	0.59
RASSF3	NM_178169	68	77	85	41	16	15	-0.757636463	0.02783	0.59
MYEF2	NM_016132	48	46	63	22	8	16	-0.755544698	0.04276	0.59
BTBD3	NM_014962	290	268	343	123	88	84	-0.755346346	0.00125	0.59
DCHS1	NM_003737	418	373	554	216	107	138	-0.753643571	0.00021	0.59
LTBP1	NM_206943	104	99	129	52	19	38	-0.751008338	0.01215	0.59
UHRF1BP1	NM_017754	60	84	80	46	8	18	-0.748964727	0.04051	0.60
KCNF1	NM_002236	67	54	70	18	16	21	-0.74880524	0.03793	0.60
PIM1	NM_002648	354	372	405	120	117	121	-0.744376241	0.00333	0.60
RAB30	NM_014488	81	97	93	45	14	30	-0.740489316	0.02295	0.60
RAB6B	NM_016577	133	131	184	55	42	46	-0.740212028	0.00903	0.60
DAAM2	NM_001201427	98	73	100	31	22	31	-0.738861421	0.02296	0.60
SOGA1	NM_080627	377	342	419	197	90	113	-0.738111986	0.00032	0.60
ZNRF3	NM_001206998	206	130	164	95	39	34	-0.737960144	0.01422	0.60
MN1	NM_002430	162	195	143	70	32	66	-0.734650662	0.01112	0.60
COL6A3	NM_004369	186	183	238	94	45	69	-0.731844845	0.00272	0.60
RECQL	NM_032941	387	291	385	244	64	75	-0.731155163	0.00903	0.60
SIX1	NM_005982	204	180	215	101	38	70	-0.730812942	0.00315	0.60
PAG1	NM_018440	171	170	241	95	46	58	-0.726361862	0.00434	0.60
C14orf93	NM_001130708	72	78	76	37	17	19	-0.721351482	0.03453	0.61
RCAN3	NM_013441	389	365	451	199	101	126	-0.720084689	0.00032	0.61
PPP1R13L	NM_006663	72	59	82	25	18	23	-0.711406346	0.04115	0.61
CLIC4	NM_013943	5948	5813	6713	3569	1354	1986	-0.711203772	7.72E-06	0.61
ATRX	NM_000489	781	766	963	481	158	294	-0.707689189	0.00026	0.61
PLEC	NM_201380	2512	2379	2616	1364	656	715	-0.707497666	4.78E-05	0.61
CHAC1	NM_024111	343	275	325	159	94	72	-0.706379641	0.00539	0.61
CYTL1	NM_018659	98	143	149	55	33	42	-0.704683266	0.0191	0.61
COL18A1	NM_030582	121	123	146	60	32	41	-0.704050007	0.01272	0.61
DAB2IP	NM_032552	499	516	644	303	124	180	-0.703041041	0.00027	0.61
SLC48A1	NM_017842	103	104	119	66	18	30	-0.702345542	0.02561	0.61
LTN1	NM_015565	337	311	377	175	66	131	-0.701947612	0.00117	0.61
KIAA1522	NM_020888	174	148	188	87	43	48	-0.700460545	0.00779	0.62
IFNGR2	NM_005534	181	192	244	138	34	53	-0.699924224	0.01492	0.62
MAST4	NM_001164664	88	73	102	37	16	34	-0.698475425	0.03373	0.62
SLC23A2	NM_203327	262	247	274	166	38	87	-0.696578514	0.00889	0.62

ME1	NM_002395	439	441	573	235	99	193	-0.695107409	0.00067	0.62
NUAK1	NM_014840	345	359	427	224	85	107	-0.694360385	0.00182	0.62
HGSNAT	NM_152419	162	194	234	83	38	84	-0.694148697	0.01047	0.62
GNG2	NM_053064	664	566	674	282	148	255	-0.69245161	0.00033	0.62
CD109	NM_133493	1100	1111	1256	654	182	473	-0.690702995	0.00187	0.62
TTBK2	NM_173500	91	87	114	58	13	31	-0.690370422	0.03671	0.62
RERE	NM_001042681	151	142	134	78	33	40	-0.690266115	0.01583	0.62
MSC	NM_005098	210	219	297	149	45	73	-0.685040078	0.0081	0.62
SETBP1	NM_015559	110	110	127	52	29	38	-0.684652872	0.02007	0.62
PROSER1	NM_025138	246	207	305	151	46	82	-0.683715121	0.00651	0.62
DPF3	NM_012074	127	129	123	74	27	34	-0.683363849	0.02261	0.62
TRIOBP	NM_001039141	388	421	525	262	89	148	-0.682085259	0.0016	0.62
SOX9	NM_000346	384	324	466	236	77	125	-0.681880537	0.00245	0.62
TMEM65	NM_194291	320	295	345	188	73	95	-0.679451994	0.00246	0.62
HAGH	NM_005326	81	70	88	31	22	25	-0.677990066	0.04439	0.63
PPAP2B	NM_003713	523	501	621	320	106	196	-0.677096914	0.00087	0.63
COL4A1	NM_001845	291	296	289	197	47	90	-0.674339349	0.01195	0.63
TTC28	NM_001145418	231	213	294	127	53	89	-0.673021959	0.00425	0.63
PREX1	NM_020820	148	184	185	111	26	55	-0.672758875	0.02282	0.63
SLC44A2	NM_020428	191	182	255	114	36	80	-0.671448962	0.01009	0.63
WIPI1	NM_017983	108	124	122	50	26	47	-0.669004512	0.02691	0.63
NACC2	NM_144653	114	104	129	35	39	37	-0.668951942	0.04056	0.63
NIN	NM_020921	242	218	344	134	55	103	-0.667661001	0.00605	0.63
THAP2	NM_031435	124	100	117	62	31	26	-0.665006032	0.03453	0.63
DAG1	NM_001177639	582	544	722	320	141	230	-0.664212613	0.00032	0.63
CCDC88C	NM_001080414	164	130	150	69	47	37	-0.663795879	0.02443	0.63
FBXL20	NM_032875	77	80	121	49	20	27	-0.662562467	0.04477	0.63
REST	NM_001193508	874	901	1260	541	214	389	-0.661811347	0.00047	0.63
RB1CC1	NM_014781	468	437	591	236	96	221	-0.661157277	0.00279	0.63
LRP1	NM_002332	429	320	401	235	82	119	-0.66069144	0.00402	0.63
ZFHX3	NM_006885	613	416	495	269	148	136	-0.660252016	0.00576	0.63
DYNC1H1	NM_001376	710	532	772	377	165	214	-0.658862635	0.00077	0.63
MANEAL	NM_001113482	101	86	127	66	21	25	-0.658641146	0.04462	0.63
GRB10	NM_005311	2688	2246	2655	1746	455	806	-0.658511287	0.00327	0.63
GAB1	NM_207123	96	98	124	42	26	41	-0.657051445	0.03549	0.63
C2orf69	NM_153689	147	143	151	90	25	49	-0.656439181	0.02341	0.63
GOLGB1	NM_004487	310	332	333	210	62	103	-0.656127257	0.00742	0.63
SPG20	NM_015087	246	213	181	149	29	68	-0.651953959	0.03666	0.64
MAML2	NM_032427	258	291	339	151	73	105	-0.651377404	0.00367	0.64
KLF2	NM_016270	133	121	159	52	48	38	-0.651239261	0.03511	0.64
POU2F1	NM_002697	173	147	222	114	29	60	-0.650939869	0.02276	0.64
PIEZO1	NM_001142864	577	545	722	341	165	185	-0.650918967	0.00093	0.64
ELF1	NM_172373	150	121	140	77	37	34	-0.648232049	0.02911	0.64
SYNE3	NM_152592	239	228	288	167	51	70	-0.647752211	0.01305	0.64
MPHOSPH8	NM_017520	162	157	145	112	26	38	-0.647188039	0.04338	0.64
CHSY1	NM_014918	364	405	281	269	54	91	-0.646135684	0.04089	0.64

SLC25A43	NM_145305	220	215	286	116	58	91	-0.644940645	0.00659	0.64
ETV1	NM_001163147	789	598	846	432	187	231	-0.64415407	0.00105	0.64
EIF4E3	NM_001134651	98	99	130	52	32	30	-0.642977686	0.04003	0.64
F3	NM_001993	280	307	387	153	70	139	-0.639053285	0.00624	0.64
PTP4A1	NM_003463	936	1007	945	697	174	291	-0.637582167	0.01046	0.64
RBBP6	NM_006910	105	128	146	68	24	47	-0.637533568	0.03419	0.64
CDKN1B	NM_004064	207	174	176	111	45	53	-0.636489581	0.0197	0.64
CAMTA2	NM_001171167	153	121	176	79	41	43	-0.635431516	0.02453	0.64
PRRC2C	NM_015172	1792	1721	2249	1166	375	735	-0.634397279	0.00088	0.64
CLIP1	NM_002956	231	182	238	115	43	87	-0.633447558	0.01209	0.64
CRAMP1L	NM_020825	146	106	124	72	26	41	-0.632130074	0.03738	0.65
WDR35	NM_001006657	139	136	210	85	29	65	-0.631728775	0.02839	0.65
ADCY9	NM_001116	124	115	137	68	25	46	-0.631546856	0.03128	0.65
RIN2	NM_001242581	132	86	124	63	22	40	-0.629762151	0.04515	0.65
FBLN1	NM_006486	318	253	269	139	71	105	-0.628592472	0.00772	0.65
ARHGAP5	NM_001030055	487	495	758	223	133	272	-0.627049442	0.01195	0.65
BLID	NM_001001786	117	117	158	87	22	39	-0.626658893	0.04531	0.65
UTRN	NM_007124	298	224	335	191	61	79	-0.626013191	0.01729	0.65
MED12	NM_005120	813	706	804	445	166	301	-0.625821386	0.0009	0.65
SMAD1	NM_005900	140	169	173	87	39	54	-0.624617903	0.02289	0.65
MAP4K4	NM_145686	588	536	776	334	187	191	-0.622043978	0.00281	0.65
AHNAK	NM_001620	849	760	746	504	197	226	-0.621568535	0.00458	0.65
HIPK3	NM_005734	221	264	354	170	53	101	-0.619758568	0.01656	0.65
COL6A1	NM_001848	7009	7044	7781	4620	1970	1980	-0.619512009	0.00205	0.65
ZNF827	NM_178835	206	287	435	175	75	96	-0.619112381	0.02445	0.65
CLDND1	NM_001040182	843	833	745	594	147	246	-0.618929133	0.01628	0.65
KLF4	NM_004235	135	124	113	62	30	45	-0.618533751	0.04013	0.65
RSF1	NM_016578	302	309	365	154	82	132	-0.618307659	0.00509	0.65
KIRREL	NM_018240	803	834	957	490	187	345	-0.618021486	0.00082	0.65
KMT2C	NM_170606	115	129	173	84	36	34	-0.617871267	0.04425	0.65
ABCC5	NM_005688	156	166	207	114	23	68	-0.616372176	0.04276	0.65
TMEM87B	NM_032824	164	188	150	105	36	52	-0.615750708	0.03556	0.65
SZT2	NM_015284	224	204	198	153	45	46	-0.615113866	0.04569	0.65
EPS8	NM_004447	167	145	209	84	50	57	-0.615096476	0.02201	0.65
CENPE	NM_001813	261	288	280	134	88	85	-0.613962345	0.01353	0.65
CCNG2	NM_004354	127	147	189	90	25	61	-0.61392389	0.03968	0.65
JMJD1C	NM_032776	292	288	303	173	58	115	-0.613651422	0.00921	0.65
FZD2	NM_001466	259	223	274	134	65	89	-0.613103129	0.00811	0.65
ELMSAN1	NM_001043318	209	175	189	91	50	73	-0.612512965	0.01843	0.65
SEPN1	NM_206926	726	638	934	430	224	220	-0.61230469	0.00367	0.65
DUSP4	NM_001394	189	173	241	108	46	75	-0.610655636	0.01577	0.65
UBN1	NM_001079514	202	252	239	157	47	69	-0.609857094	0.02783	0.66
NR3C1	NM_001018076	440	483	505	297	100	172	-0.609777537	0.00483	0.66
TAOK1	NM_020791	1177	1206	1932	757	335	570	-0.608718648	0.00246	0.66
FAM127B	NM_001078172	300	263	265	136	83	92	-0.608171523	0.01195	0.66
PPP1CC	NM_002710	3397	2682	2708	1996	662	912	-0.607380134	0.00533	0.66

IRF2BP2	NM_001077397	3133	3267	3532	1923	902	1096	-0.606593482	0.00023	0.66
LRFN4	NM_024036	276	207	314	159	48	104	-0.605791146	0.01838	0.66
FN1	NM_212482	4220	3589	5296	2185	1201	1642	-0.604570403	0.00022	0.66
TMEM132B	NM_052907	154	135	161	60	43	60	-0.604368773	0.03556	0.66
CREB3L2	NM_194071	1020	742	1064	475	281	317	-0.60170887	0.00231	0.66
TGIF2	NM_001199514	143	132	168	91	29	51	-0.600600729	0.03628	0.66
IGF2R	NM_000876	725	688	856	380	214	280	-0.600580088	0.00086	0.66
SEC11A	NM_014300	598	515	566	384	115	183	-0.599873655	0.00919	0.66
C9orf91	NM_153045	194	218	248	125	45	87	-0.599393345	0.01829	0.66
GBP5	NM_001134486	210	189	254	111	72	58	-0.59893121	0.027	0.66
PTPN13	NM_080685	300	317	365	199	76	113	-0.598383699	0.0077	0.66
ZNF217	NM_006526	197	179	235	130	42	68	-0.596314322	0.02352	0.66
PCDHB2	NM_018936	122	121	163	65	28	59	-0.594677464	0.04477	0.66
MAP1LC3A	NM_032514	187	146	147	73	53	49	-0.594402126	0.04584	0.66
SLC7A5	NM_003486	1925	1821	1991	898	615	656	-0.593206903	0.00205	0.66
TENM4	NM_001098816	165	122	170	86	27	63	-0.592341089	0.04281	0.66
SH3BGRL	NM_003022	606	616	784	355	172	260	-0.591290816	0.00135	0.66
PPDPF	NM_024299	3494	3504	4172	1876	1159	1253	-0.590042006	0.00082	0.66
ULBP1	NM_025218	153	153	193	70	54	58	-0.589723612	0.03759	0.66
GORASP2	NM_001201428	423	405	435	302	72	146	-0.589134658	0.02284	0.66
TMED5	NM_016040	477	544	493	315	142	143	-0.587383406	0.01269	0.67
MVP	NM_005115	777	516	751	371	192	231	-0.586913759	0.00522	0.67
HIAT1	NM_033055	162	154	154	102	29	55	-0.586784366	0.04474	0.67
ARPIN	NM_182616	165	173	184	98	42	63	-0.584962622	0.02717	0.67
PTPN14	NM_005401	1646	1358	1780	1041	335	591	-0.584558133	0.00271	0.67
KHSRP	NM_003685	1956	2132	2573	1410	477	847	-0.583840609	0.00197	0.67
KLHL11	NM_018143	214	176	289	131	53	80	-0.58239103	0.024	0.67
SNAP23	NM_003825	489	498	631	263	121	249	-0.582047648	0.00714	0.67
PTGS2	NM_000963	221	230	377	132	71	110	-0.581075744	0.02511	0.67
KLHL42	NM_020782	170	151	208	92	46	65	-0.579767083	0.02788	0.67
SFRP1	NM_003012	2101	1686	2592	1104	617	760	-0.579023078	0.00117	0.67
SYNE2	NM_182914	169	165	217	127	40	53	-0.572516713	0.04601	0.67
ECM1	NM_004425	896	701	896	414	224	343	-0.572485771	0.00231	0.67
ZNF638	NM_001014972	594	558	698	382	132	243	-0.571313826	0.0046	0.67
COL27A1	NM_032888	288	292	351	163	77	128	-0.569618147	0.01055	0.67
RNF167	NM_015528	776	570	773	449	169	246	-0.569550032	0.00576	0.67
PLXNA3	NM_017514	290	248	309	122	92	105	-0.569081308	0.02198	0.67
SLC35E2B	NM_001110781	270	243	305	165	89	61	-0.567058483	0.04489	0.67
RAB6A	NM_002869	2447	1907	2782	1322	561	1005	-0.565844816	0.00127	0.68
MARK4	NM_001199867	414	379	415	245	84	165	-0.565621652	0.01073	0.68
SMYD2	NM_020197	235	238	275	180	43	86	-0.564395427	0.04306	0.68
TNRC6A	NM_014494	307	327	605	261	80	154	-0.564002802	0.04231	0.68
TOX2	NM_001098797	379	387	506	251	111	149	-0.563291412	0.00768	0.68
MTHFD2	NM_006636	4715	4661	5621	2764	1368	1938	-0.562519461	0.00011	0.68
SGCB	NM_000232	2080	1918	2370	1376	513	758	-0.562137808	0.00125	0.68
CPSF1	NM_013291	653	419	712	358	131	232	-0.562020816	0.01422	0.68

CNTRL	NM_007018	174	154	214	100	38	76	-0.561615832	0.03799	0.68
MAN1A1	NM_005907	540	465	591	337	104	219	-0.560182729	0.01138	0.68
PIK3CA	NM_006218	344	383	414	247	83	140	-0.560009798	0.0146	0.68
PARP3	NM_001003931	174	176	194	108	44	65	-0.559851487	0.03415	0.68
SGPP1	NM_030791	317	297	403	152	84	159	-0.559137607	0.02017	0.68
ZNF625	NM_145233	253	291	349	167	90	94	-0.559134666	0.02127	0.68
RALGDS	NM_006266	194	149	177	85	53	62	-0.556023177	0.04432	0.68
SGMS1	NM_147156	227	208	281	117	46	119	-0.555923885	0.04115	0.68
DOCK1	NM_001380	385	300	426	232	77	146	-0.555765726	0.01683	0.68
ANP32B	NM_006401	2487	2212	2579	1346	480	1172	-0.555232193	0.0085	0.68
MEGF9	NM_001080497	149	146	164	89	38	55	-0.554639026	0.04558	0.68
CAT	NM_001752	225	227	212	135	58	75	-0.552202067	0.03284	0.68
PMP22	NM_153322	583	635	625	343	157	252	-0.551769148	0.00503	0.68
CHD6	NM_032221	181	190	212	109	48	76	-0.550925198	0.03241	0.68
SARS	NM_006513	1581	1485	1574	1070	306	604	-0.550705722	0.01073	0.68
FBXL7	NM_012304	638	602	669	346	210	198	-0.550685954	0.01063	0.68
PXDN	NM_012293	2837	2010	2946	1762	689	768	-0.550489288	0.01112	0.68
PRKACA	NM_002730	869	616	1134	495	232	321	-0.54806181	0.01063	0.68
ZCCHC14	NM_015144	482	426	546	270	136	181	-0.547692391	0.00547	0.68
THBS1	NM_003246	26436	24762	29712	15425	8736	8130	-0.546891483	0.00504	0.68
OBSL1	NM_015311	182	173	224	113	53	65	-0.545225201	0.03698	0.69
CNOT4	NM_001190850	811	818	799	584	211	224	-0.545118451	0.02445	0.69
MAP4	NM_002375	1514	1302	1648	929	389	538	-0.54465533	0.0014	0.69
PALM2	NM_001037293	571	588	720	351	118	305	-0.543302805	0.02103	0.69
LIN54	NM_194282	224	176	198	111	59	68	-0.538929913	0.04274	0.69
OLFML2B	NM_015441	314	450	426	278	99	119	-0.537778758	0.04401	0.69
EAF1	NM_033083	370	337	463	220	106	149	-0.536847834	0.01105	0.69
MDK	NM_001012334	2607	2439	3072	1256	799	1166	-0.536753827	0.00316	0.69
GPATCH2	NM_018040	242	192	218	133	46	89	-0.536189998	0.04228	0.69
FADS1	NM_013402	1840	1579	1561	1215	349	593	-0.535432742	0.02	0.69
SESN2	NM_031459	339	327	332	193	80	139	-0.533947397	0.01815	0.69
MYADM	NM_001020820	1056	837	1298	729	280	321	-0.533727733	0.01683	0.69
MNT	NM_020310	238	193	227	120	60	85	-0.533305603	0.03376	0.69
CD9	NM_001769	395	337	430	198	101	171	-0.532385982	0.01411	0.69
MOB1B	NM_173468	482	447	618	279	111	245	-0.530577093	0.01832	0.69
SNX12	NM_013346	650	475	472	385	136	152	-0.530246262	0.04833	0.69
NFIX	NM_002501	830	784	1135	541	243	351	-0.529886454	0.00458	0.69
ASAP2	NM_003887	859	735	839	359	278	309	-0.529446421	0.01783	0.69
RIN3	NM_024832	344	291	404	166	110	134	-0.528040035	0.02295	0.69
CXCL12	NM_001178134	3274	2867	3847	1958	902	1304	-0.527656127	0.00055	0.69
BOD1L1	NM_148894	450	426	446	256	117	175	-0.527438149	0.01045	0.69
LTBP2	NM_000428	354	354	408	226	104	131	-0.526811957	0.0148	0.69
SDC3	NM_014654	2134	1968	2507	1057	773	751	-0.526418992	0.01219	0.69
ZNF800	NM_176814	339	303	446	228	81	144	-0.526063974	0.02338	0.69
CAMSAP1	NM_015447	529	451	625	304	172	171	-0.526036714	0.0161	0.69
CTNNBIP1	NM_001012329	776	714	871	378	224	351	-0.524127594	0.00765	0.70

NEFM	NM_005382	256	260	314	127	85	116	-0.524008183	0.03351	0.70
GPX8	NM_001008397	1208	1159	1318	696	311	536	-0.522913065	0.00235	0.70
PIP5K1C	NM_012398	406	391	435	225	116	165	-0.522772531	0.01138	0.70
SH3PXD2B	NM_001017995	2085	1587	2652	1216	642	708	-0.522227358	0.00981	0.70
ANKRD52	NM_173595	313	252	393	180	83	128	-0.521325674	0.02524	0.70
PHKB	NM_000293	265	226	266	136	61	113	-0.521032015	0.03376	0.70
SMS	NM_004595	989	1048	938	703	231	355	-0.520594358	0.01938	0.70
SMURF2	NM_022739	818	782	1048	564	209	352	-0.517552046	0.00716	0.70
RAB14	NM_016322	985	953	1157	586	244	472	-0.516092902	0.00599	0.70
CRTC3	NM_022769	302	281	328	174	85	117	-0.516065652	0.02097	0.70
LRP5	NM_002335	296	272	360	138	111	111	-0.515612465	0.0463	0.70
RSRC2	NM_023012	717	672	791	444	212	257	-0.513923747	0.00654	0.70
CMIP	NM_198390	777	846	1006	589	236	296	-0.509975303	0.01257	0.70
PTBP3	NM_001163790	250	273	345	169	82	107	-0.509546465	0.03208	0.70
ZC3HAV1	NM_020119	573	485	670	261	197	223	-0.509195075	0.02633	0.70
CRMP1	NM_001014809	594	560	593	352	151	239	-0.506137801	0.00984	0.70
MTA1	NM_004689	628	585	710	408	193	208	-0.504222578	0.01521	0.71
EHD3	NM_014600	325	268	383	218	81	115	-0.502099722	0.03663	0.71
JAK1	NM_002227	794	886	1146	719	200	324	-0.501151342	0.04241	0.71
MLLT11	NM_006818	1142	1111	1272	652	322	512	-0.501043051	0.00344	0.71
EIF4G3	NM_001198802	1153	966	1206	623	308	469	-0.500392505	0.00383	0.71
KHDRBS1	NM_006559	2348	2494	3641	1677	686	1236	-0.500001969	0.01046	0.71
H1F0	NM_005318	1369	1374	1227	887	345	494	-0.499950819	0.01392	0.71
EPHX1	NM_001136018	630	569	635	328	187	247	-0.498525786	0.0111	0.71
ANKH	NM_054027	386	391	449	220	124	164	-0.498026614	0.01883	0.71
PCYOX1	NM_016297	216	207	256	135	55	95	-0.497555353	0.0466	0.71
MEX3C	NM_016626	456	491	557	319	143	179	-0.493000225	0.01881	0.71
SRCAP	NM_006662	836	796	1135	623	236	338	-0.489935064	0.01595	0.71
LMNA	NM_170707	8816	8256	9254	5178	2257	3945	-0.489774542	0.00202	0.71
PLXNA1	NM_032242	500	561	643	382	144	213	-0.489269463	0.02172	0.71
FYCO1	NM_024513	597	592	623	316	197	239	-0.486868399	0.01854	0.71
WASL	NM_003941	678	679	796	458	197	274	-0.486335636	0.01006	0.71
SASH1	NM_015278	469	402	631	282	125	227	-0.482930864	0.03053	0.72
LIX1L	NM_153713	2541	2071	2749	1667	718	802	-0.482315591	0.01474	0.72
ZNF598	NM_178167	872	695	847	551	193	318	-0.481928944	0.01965	0.72
ADNP	NM_181442	303	320	439	183	102	155	-0.481666139	0.04166	0.72
CHP1	NM_007236	440	539	752	332	169	223	-0.481600015	0.03779	0.72
PRDM4	NM_012406	325	358	427	227	111	133	-0.480030486	0.03494	0.72
ANKRD11	NM_013275	959	964	1459	638	331	452	-0.479979377	0.01591	0.72
TAB2	NM_015093	831	711	1009	386	209	458	-0.479070192	0.04979	0.72
ZNF532	NM_018181	1207	1148	1496	778	396	468	-0.478391177	0.00811	0.72
KIAA1143	NM_020696	379	320	457	256	105	137	-0.476157654	0.03831	0.72
DDAH1	NM_012137	346	284	340	197	91	127	-0.475282468	0.03738	0.72
ATL3	NM_015459	3340	3160	5045	2237	865	1873	-0.473123566	0.03082	0.72
SURF4	NM_033161	1257	1222	1485	944	260	579	-0.472949743	0.04042	0.72
MFAP4	NM_001198695	1423	1387	1813	952	355	719	-0.472504399	0.01564	0.72

SKAP2	NM_003930	314	281	324	202	73	126	-0.472017338	0.04669	0.72
WIPI2	NM_016003	5251	4833	5370	2754	1722	2012	-0.47095122	0.00836	0.72
NIPBL	NM_133433	1076	924	1303	751	252	461	-0.470704636	0.02158	0.72
PDE12	NM_177966	359	347	496	204	118	179	-0.470009979	0.04228	0.72
ITGA6	NM_001079818	1311	1162	1422	940	310	498	-0.46860925	0.02125	0.72
NOL4L	NM_080616	483	450	507	311	143	171	-0.467166534	0.02818	0.72
SPEN	NM_015001	657	472	705	364	154	274	-0.466745209	0.03038	0.72
WBP2	NM_012478	456	553	488	306	150	191	-0.46641142	0.04042	0.72
GLUL	NM_001033056	716	745	786	457	204	321	-0.465556413	0.01354	0.72
ARID1A	NM_006015	1539	1360	1594	1033	352	631	-0.464841576	0.01668	0.72
FARP1	NM_005766	368	306	450	235	105	145	-0.461819767	0.04115	0.73
PRRC2A	NM_004638	2536	2702	3011	1939	805	916	-0.460975289	0.02158	0.73
PPP6R3	NM_001164160	2717	2426	3106	1521	691	1370	-0.460421957	0.01477	0.73
CHMP1B	NM_020412	678	528	783	365	153	339	-0.459579229	0.04845	0.73
EXOSC10	NM_001001998	1085	1059	1039	684	282	449	-0.458118616	0.0159	0.73
UBQLN2	NM_013444	2099	2004	2241	1243	668	836	-0.457695337	0.00628	0.73
SBF1	NM_002972	602	577	708	447	180	208	-0.456734843	0.04013	0.73
POFUT1	NM_015352	459	423	516	325	125	170	-0.455765018	0.03857	0.73
GPD2	NM_000408	531	504	665	367	161	217	-0.455137009	0.02483	0.73
PLXNB2	NM_012401	463	363	525	249	142	184	-0.451947536	0.04058	0.73
UBR7	NM_175748	1442	1360	1286	924	355	564	-0.450795235	0.02345	0.73
SH3BP4	NM_014521	1207	1255	1609	743	398	614	-0.450596774	0.01472	0.73
JAM3	NM_032801	591	483	703	394	140	257	-0.448957996	0.04042	0.73
MAPK1	NM_002745	1032	1091	1298	750	317	461	-0.44384902	0.01482	0.74
SRRM2	NM_016333	7707	5819	7377	4465	2010	2831	-0.443274593	0.00887	0.74
PCNXL2	NM_014801	505	514	578	364	164	179	-0.442851859	0.04838	0.74
PTPN23	NM_015466	460	445	586	316	152	185	-0.442657704	0.03775	0.74
SLTM	NM_024755	881	938	1090	639	250	418	-0.442398691	0.02007	0.74
STK24	NM_001032296	771	826	903	527	270	301	-0.440523684	0.02949	0.74
ARL6IP5	NM_006407	872	782	1012	577	251	362	-0.437500348	0.01692	0.74
RBM25	NM_021239	1669	1492	1962	1108	441	757	-0.437408486	0.01315	0.74
XPO1	NM_003400	1587	1486	2021	1278	408	654	-0.437117255	0.04166	0.74
YWHAQ	NM_006826	6988	5348	6437	3893	1530	3041	-0.435656668	0.02599	0.74
LOXL2	NM_002318	1579	1407	1981	1051	499	651	-0.434109215	0.0144	0.74
CYR61	NM_001554	948	871	806	499	252	411	-0.430738177	0.04217	0.74
HMGA1	NM_145901	14955	15317	15991	10860	4913	5061	-0.430481408	0.03735	0.74
WIPF1	NM_003387	671	660	781	489	214	245	-0.430202001	0.04274	0.74
CBFB	NM_022845	1035	712	1014	553	288	369	-0.429903437	0.03831	0.74
PHGDH	NM_006623	3087	2981	3956	1827	1166	1322	-0.423387341	0.02781	0.75
ARFGEF2	NM_006420	845	757	849	571	213	341	-0.422223047	0.03549	0.75
ACSL4	NM_022977	708	705	724	421	191	348	-0.420648544	0.04176	0.75
SEC22B	NM_004892	793	650	923	545	223	304	-0.420038636	0.04068	0.75
HIVEP3	NM_001127714	1051	1006	1023	631	342	400	-0.41627452	0.03511	0.75
NRP2	NM_201266	2829	2399	2814	1758	921	914	-0.412018462	0.04601	0.75
G3BP2	NM_203505	2749	2256	2970	1550	833	1168	-0.411802804	0.01497	0.75
MAP1B	NM_005909	3890	3621	3895	2515	1217	1447	-0.411272039	0.0203	0.75

ATN1	NM_001007026	1483	1478	1762	978	481	673	-0.41092578	0.0144	0.75
CDCA7L	NM_018719	837	708	829	532	248	298	-0.408604231	0.04365	0.75
NUMA1	NM_006185	4668	3918	5821	3059	1275	2250	-0.404503417	0.02639	0.76
LARP1	NM_015315	7912	6567	10125	5391	2245	3639	-0.402250869	0.02561	0.76
RALGAPB	NM_020336	769	674	843	430	225	370	-0.400706047	0.04477	0.76
PDZD8	NM_173791	642	671	782	424	193	337	-0.398916729	0.0485	0.76
MGEA5	NM_012215	1152	1175	1388	784	387	522	-0.396779242	0.02476	0.76
ETV5	NM_004454	1182	1203	1390	739	401	563	-0.395724177	0.02788	0.76
BNIP3L	NM_004331	1512	1614	1971	1117	524	695	-0.394964556	0.02811	0.76
CDC42EP3	NM_006449	2654	2722	3228	1465	956	1362	-0.39442127	0.04766	0.76
NAV1	NM_020443	2138	2187	2413	1580	707	850	-0.386984114	0.04042	0.76
DLG5	NM_004747	1574	1528	1942	1099	503	733	-0.384098425	0.02453	0.77
NLN	NM_020726	1041	1053	1173	738	291	506	-0.383287065	0.04519	0.77
CTNND1	NM_001085458	1117	1057	1415	774	385	485	-0.383028966	0.04077	0.77
ABL1	NM_005157	1035	918	1171	639	328	466	-0.378329808	0.03759	0.77
PFN2	NM_053024	2550	2551	3219	1705	826	1324	-0.372013377	0.03045	0.77
TIMP2	NM_003255	1654	1663	1866	1139	491	813	-0.370484201	0.03567	0.77
CTNNA1	NM_001903	5816	5234	7218	4145	1978	2540	-0.343778342	0.04802	0.79
RPS6	NM_001010	13602	12416	13972	14428	6469	10206	0.322938165	0.04615	1.25
SLC25A5	NM_001152	10122	10704	12050	11771	5467	8519	0.339522557	0.03738	1.27
RPS2	NM_002952	22474	17852	22275	21310	10457	17183	0.347530816	0.0469	1.27
EEF1B2	NM_001037663	3682	3111	3519	3586	1768	2742	0.349271986	0.04314	1.27
CTTN	NM_001184740	1878	1986	2501	2269	1148	1582	0.357898553	0.04164	1.28
ERGIC3	NM_198398	950	916	1109	1013	536	791	0.364090294	0.04295	1.29
SEMA7A	NM_003612	2002	1908	2319	2332	1050	1598	0.364457769	0.02481	1.29
AIFM1	NM_004208	1133	1101	1169	1289	602	838	0.364557696	0.04068	1.29
H2AFZ	NM_002106	8703	8558	10364	9614	5180	6855	0.365662515	0.02261	1.29
GET4	NM_015949	2318	1973	2264	2267	1275	1604	0.367017192	0.04164	1.29
RPS15	NM_001018	11288	9587	11103	11533	6070	7692	0.369440203	0.02719	1.29
NSUN2	NM_017755	821	820	998	903	454	741	0.371449612	0.04801	1.29
DHCR24	NM_014762	1675	1633	1882	1809	966	1327	0.371588837	0.02719	1.29
RPL18A	NM_000980	11017	10106	11238	10515	6109	8725	0.371852268	0.03271	1.29
YIF1A	NM_020470	856	892	974	953	483	738	0.374150379	0.04157	1.30
PSMD2	NM_002808	2758	2410	2884	3241	1215	2193	0.377023181	0.04057	1.30
HIST1H3I	NM_003533	7794	6757	9195	8796	3729	6702	0.37730191	0.03112	1.30
CD81	NM_004356	886	790	928	1011	436	676	0.380103001	0.03486	1.30
MGST3	NM_004528	3994	4073	4239	4429	2418	2964	0.380911878	0.02934	1.30
RPF1	NM_025065	858	729	870	861	431	680	0.381141125	0.04068	1.30
ID1	NM_002165	2963	2588	3657	3687	1615	2176	0.381728089	0.03015	1.30
IARS2	NM_018060	803	775	791	914	399	635	0.387056439	0.04069	1.31
CFL1	NM_005507	17927	16402	17780	16876	10930	13125	0.38847322	0.04075	1.31
POLD2	NM_006230	2586	2308	2686	2866	1399	1868	0.389191172	0.01595	1.31
BSG	NM_001728	3697	3211	3701	3297	2214	2803	0.391106092	0.04729	1.31
PSMB4	NM_002796	2307	2132	2485	2297	1377	1823	0.394869612	0.0253	1.31
C19orf53	NM_014047	1519	1341	1654	1390	845	1349	0.39537197	0.0483	1.32
UFD1L	NM_005659	646	614	702	764	353	495	0.397704911	0.0297	1.32

OSTC	NM_021227	873	873	1044	979	545	726	0.403575218	0.02484	1.32
KARS	NM_001130089	995	984	1072	1121	581	785	0.404259583	0.02027	1.32
MAGOH	NM_002370	710	815	811	783	449	662	0.404354556	0.04723	1.32
RBM17	NM_032905	844	845	976	1012	531	630	0.404810003	0.02911	1.32
HJURP	NM_018410	2046	1893	2476	2054	1244	1817	0.406273422	0.02861	1.33
RPS4X	NM_001007	29975	27513	31022	29198	15424	27257	0.406393257	0.02604	1.33
ERF	NM_006494	897	803	961	1049	476	678	0.408591498	0.01854	1.33
RNF115	NM_014455	1130	907	1029	1074	603	814	0.409290006	0.03233	1.33
EIF3B	NM_001037283	1871	1416	1903	1829	1057	1296	0.40987859	0.02945	1.33
C7orf50	NM_001134396	3163	3125	3601	3102	1965	2831	0.410188681	0.02936	1.33
APLP2	NM_001642	652	598	716	776	347	510	0.410422488	0.02295	1.33
PLAUR	NM_002659	2093	1929	2408	1970	1355	1769	0.411756659	0.03968	1.33
FMNL3	NM_175736	389	349	413	424	196	336	0.412284474	0.04654	1.33
MYBBP1A	NM_001105538	1340	1285	1519	1601	758	1060	0.412413672	0.01121	1.33
PSMB7	NM_002799	1408	1359	1749	1415	999	1159	0.413001566	0.04769	1.33
NCAPD2	NM_014865	4487	3650	4740	4406	2646	3292	0.414686542	0.01949	1.33
BRK1	NM_018462	1298	1098	1323	1238	811	931	0.415339031	0.03799	1.33
TBL3	NM_006453	545	432	547	557	314	375	0.415967687	0.04276	1.33
ATP13A3	NM_024524	868	932	1122	1004	507	891	0.416140071	0.03422	1.33
HDGF	NM_004494	6379	5665	7179	7124	3887	4617	0.416302832	0.01248	1.33
NOP14	NM_003703	721	597	612	691	398	495	0.416736632	0.04723	1.33
CRCP	NM_001142414	494	378	512	494	282	355	0.418965454	0.04492	1.34
MRPL37	NM_016491	2379	1997	2575	2406	1393	1825	0.419077588	0.01508	1.34
MRPL20	NM_017971	1448	1364	1565	1581	734	1322	0.419171504	0.01942	1.34
ABRACL	NM_021243	360	308	370	378	205	273	0.41939484	0.0452	1.34
DAD1	NM_001344	2952	2612	3210	3253	1555	2437	0.419700218	0.00727	1.34
SYPL1	NM_006754	531	537	711	611	338	504	0.42030165	0.03549	1.34
SHCBP1	NM_024745	550	509	468	634	266	405	0.42098154	0.04993	1.34
FAM96B	NM_016062	532	468	565	553	279	463	0.421770575	0.03237	1.34
GNG12	NM_018841	3641	3144	4800	4137	2310	2945	0.421978578	0.02024	1.34
HSPD1	NM_002156	5694	5436	6633	5237	3728	5172	0.422160937	0.04011	1.34
MCM6	NM_005915	1742	1515	1959	1613	1167	1367	0.422729888	0.04312	1.34
EEF1A1	NM_001402	48806	46126	53371	55375	23922	45128	0.423086466	0.01379	1.34
LAMB1	NM_002291	1073	1060	1217	1198	716	817	0.424722034	0.02398	1.34
EXOSC2	NM_014285	464	483	633	552	323	417	0.426365636	0.03799	1.34
GADD45GIP1	NM_052850	5760	5470	6484	6085	3402	4976	0.427013582	0.00828	1.34
SSR1	NM_003144	1132	1178	1304	1295	624	1094	0.427048667	0.01949	1.34
NPLOC4	NM_017921	768	752	893	884	508	588	0.427439659	0.02376	1.34
FUT8	NM_178156	4251	4055	4726	4133	2878	3431	0.427893978	0.02874	1.35
ABHD2	NM_152924	1934	1646	2250	2237	1191	1372	0.427989106	0.01742	1.35
MFN2	NM_001127660	1338	1148	1556	1438	780	1095	0.428477504	0.01272	1.35
POLR2F	NM_021974	356	337	406	412	179	339	0.429814034	0.0449	1.35
EIF3D	NM_003753	2123	2074	2167	2360	1225	1702	0.431175704	0.00887	1.35
FAM129B	NM_022833	2725	2444	2976	2825	1847	1924	0.431941819	0.02874	1.35
EIF2S2	NM_003908	571	453	621	576	321	459	0.432392033	0.02904	1.35
SUMO3	NM_006936	1045	994	1127	1289	529	879	0.432679564	0.01295	1.35

NUDC	NM_006600	1441	1311	1459	1518	752	1245	0.433124648	0.01285	1.35
PTK2	NM_005607	1079	994	1162	1235	549	944	0.433428812	0.01256	1.35
DEPDC1	NM_001114120	584	588	572	568	368	497	0.433535232	0.04453	1.35
SOD2	NM_001024465	787	668	834	799	469	611	0.433660507	0.02047	1.35
VKORC1	NM_024006	726	821	858	840	501	641	0.433941459	0.02708	1.35
PRPF6	NM_012469	455	365	460	474	230	371	0.434835745	0.03167	1.35
EIF4A1	NM_001204510	12713	11503	14065	14092	6440	11599	0.435241644	0.00865	1.35
DNAJB1	NM_006145	746	612	823	792	502	493	0.436719458	0.04519	1.35
R3HDM1	NM_015361	702	552	934	767	464	558	0.437234488	0.04494	1.35
ZNF330	NM_014487	394	321	429	387	238	317	0.437611609	0.04228	1.35
USP14	NM_005151	668	565	754	778	308	615	0.438017519	0.03214	1.35
GINS2	NM_016095	357	335	477	433	211	337	0.438432405	0.036	1.36
MRPL9	NM_031420	762	737	788	762	502	610	0.439396304	0.03082	1.36
TCEB2	NM_207013	2073	1671	2068	2167	1105	1578	0.440353339	0.00779	1.36
UBE2K	NM_005339	697	723	842	713	473	663	0.440593461	0.03453	1.36
DDX23	NM_004818	502	479	551	613	296	387	0.44085253	0.01889	1.36
CENPN	NM_001100624	984	1133	1164	1238	769	719	0.442291062	0.04817	1.36
BDKRB1	NM_000710	465	385	434	556	256	292	0.442395638	0.04288	1.36
ZWINT	NM_032997	1220	1053	1460	1291	698	1103	0.442516565	0.01483	1.36
ASPSCR1	NM_024083	280	262	351	336	172	244	0.443377204	0.03759	1.36
COPS8	NM_006710	2041	1786	2293	2309	1232	1547	0.443422895	0.00707	1.36
KCNQ5	NM_001160133	272	267	344	359	165	229	0.443440111	0.03808	1.36
PPAN	NM_020230	532	525	703	556	374	509	0.44502434	0.04044	1.36
RPL32	NM_001007074	5288	5575	5529	6041	3721	3828	0.445118242	0.0253	1.36
RNASEH2A	NM_006397	3085	2872	2706	3794	1588	2158	0.446471817	0.01817	1.36
UBE2N	NM_003348	471	415	499	479	235	460	0.446736425	0.04101	1.36
CPSF4	NM_006693	321	255	362	376	156	275	0.446793642	0.04206	1.36
MCM7	NM_005916	1413	1318	1248	1438	673	1298	0.447152655	0.03163	1.36
EXOC7	NM_001145299	401	397	407	460	233	332	0.449354423	0.02441	1.37
COPS6	NM_006833	3015	2893	2856	3602	1619	2313	0.450314744	0.0077	1.37
PAK1IP1	NM_017906	465	395	525	530	251	395	0.450448824	0.01883	1.37
CKB	NM_001823	293	297	346	342	200	245	0.452224459	0.03567	1.37
ILF2	NM_004515	1541	1430	1801	1816	828	1413	0.4527704	0.00657	1.37
ATP5C1	NM_001001973	875	945	1081	1088	582	770	0.453135493	0.01025	1.37
C11orf73	NM_016401	381	345	528	466	256	330	0.453136023	0.03345	1.37
CD63	NM_001780	1352	1186	1303	1564	719	1008	0.453474593	0.00743	1.37
HIST1H2AL	NM_003511	2167	2018	2135	2702	1162	1606	0.454006437	0.00744	1.37
USP39	NM_006590	593	727	771	913	381	530	0.454190623	0.02437	1.37
POLR2E	NM_002695	1068	806	846	1059	546	719	0.455526569	0.02468	1.37
MRPS16	NM_016065	847	887	932	951	531	761	0.455978912	0.01216	1.37
TCP1	NM_030752	2558	2118	2713	2897	1228	2229	0.456047232	0.00831	1.37
CIRH1A	NM_032830	1045	945	1149	1308	547	862	0.459613502	0.00648	1.38
CDC20	NM_001255	1630	1596	1270	1567	999	1237	0.460156113	0.04115	1.38
RPL37	NM_000997	828	964	886	1132	550	641	0.460542778	0.02384	1.38
SFT2D1	NM_145169	541	493	614	620	364	402	0.460553647	0.02098	1.38
SNX17	NM_014748	799	579	693	824	420	531	0.462180545	0.02127	1.38

TSR1	NM_018128	769	686	727	812	471	563	0.463388373	0.01628	1.38
SUPT4H1	NM_003168	556	564	617	564	389	493	0.464727826	0.02839	1.38
TMA7	NM_015933	591	496	540	793	257	427	0.46474927	0.03779	1.38
NT5DC2	NM_001134231	1731	1485	1971	1557	1245	1417	0.464883323	0.03738	1.38
CCDC34	NM_030771	229	215	283	245	144	228	0.465209693	0.04814	1.38
DDX50	NM_024045	306	324	401	343	219	304	0.465346024	0.03738	1.38
TSTA3	NM_003313	372	344	426	368	252	333	0.466128819	0.03567	1.38
NOC2L	NM_015658	1625	1124	1577	1842	860	1040	0.466590948	0.01782	1.38
BIRC5	NM_001012271	1934	1859	1936	1872	1440	1408	0.466714408	0.03831	1.38
FABP5	NM_001444	1816	1787	1923	2042	1346	1245	0.468713857	0.02861	1.38
ABCE1	NM_002940	2415	2217	2586	2855	1424	1890	0.468992529	0.00235	1.38
LIN7C	NM_018362	648	576	787	646	415	618	0.469085818	0.02395	1.38
ANGPT1	NM_001146	221	206	260	293	107	212	0.470422184	0.04629	1.39
SRSF7	NM_001031684	1661	1556	2143	1699	1158	1575	0.470565574	0.01883	1.39
FXYD5	NM_014164	2897	2555	3642	3235	2030	2302	0.470619024	0.01216	1.39
UBE2J1	NM_016021	267	306	372	363	191	260	0.472908539	0.02809	1.39
EARS2	NM_001083614	272	268	330	351	164	242	0.473228193	0.02221	1.39
SLC5A6	NM_021095	1294	1196	1425	1445	910	937	0.473364992	0.01571	1.39
MRPL23	NM_021134	678	660	714	665	417	653	0.473936898	0.02198	1.39
MRFAP1	NM_033296	3007	2904	3273	3488	2046	2239	0.474285711	0.00799	1.39
WDHD1	NM_007086	283	258	320	351	159	242	0.474514993	0.02161	1.39
COPE	NM_007263	787	634	729	871	406	596	0.477119419	0.00871	1.39
FTSJ2	NM_013393	499	470	554	580	253	501	0.477563833	0.01908	1.39
MDFIC	NM_001166345	230	230	300	288	145	226	0.477773096	0.03006	1.39
CCDC124	NM_001136203	1203	969	1257	1303	741	871	0.477874836	0.00855	1.39
PREB	NM_013388	465	379	473	509	267	359	0.478561586	0.01305	1.39
SRPRB	NM_021203	364	445	435	500	222	377	0.479551536	0.02295	1.39
TMEM147	NM_032635	797	686	778	902	457	597	0.479626579	0.00686	1.39
RNF14	NM_183400	311	276	369	326	174	325	0.479929179	0.03681	1.39
RING1	NM_002931	422	339	373	393	251	324	0.480429928	0.03013	1.40
HIST1H3C	NM_003531	10059	8643	9492	11451	5320	7775	0.48048821	0.00194	1.40
HIGD2A	NM_138820	1954	1575	1887	1903	1211	1452	0.481219973	0.01051	1.40
UQCRQ	NM_014402	1165	1050	1050	1391	702	766	0.481708735	0.01552	1.40
DNAJA3	NM_005147	407	326	455	423	244	348	0.481724411	0.01931	1.40
SFXN1	NM_022754	2201	2200	2177	2523	948	2464	0.482476545	0.04111	1.40
LMLN	NM_001136049	159	146	201	206	105	133	0.482925041	0.0485	1.40
ARMC8	NM_015396	413	349	427	362	245	404	0.484218342	0.04159	1.40
RCE1	NM_005133	414	379	466	480	240	373	0.485000476	0.01073	1.40
C3orf14	NM_020685	249	264	310	314	163	240	0.485604173	0.02242	1.40
BCS1L	NM_001079866	354	348	397	419	217	319	0.485938283	0.01296	1.40
EEF1D	NM_001130053	5841	5403	5201	6953	2867	4855	0.486395951	0.00598	1.40
PAFAH1B3	NM_001145940	257	232	241	329	141	186	0.487153541	0.03276	1.40
EDF1	NM_003792	1300	1075	1207	1196	854	966	0.487959374	0.01997	1.40
NCLN	NM_020170	628	574	675	651	432	508	0.488019021	0.01497	1.40
EIF1AX	NM_001412	805	763	970	1113	423	731	0.488248999	0.00725	1.40
ITGA2	NM_002203	828	742	884	1017	463	673	0.48886016	0.00347	1.40

RPL29	NM_000992	7532	7631	9495	9110	4772	7303	0.490010856	0.00175	1.40
SOD1	NM_000454	3264	2773	3126	3153	1970	2664	0.49016268	0.00657	1.40
MRPS6	NM_032476	1033	1092	1168	1109	728	959	0.490165916	0.01146	1.40
TXN	NM_003329	1921	1599	2235	1967	1275	1614	0.492806311	0.00835	1.41
TMEM258	NM_014206	558	425	532	607	309	410	0.494232425	0.01047	1.41
PDLIM7	NM_005451	985	891	1180	1171	638	824	0.494589313	0.00367	1.41
RPS11	NM_001015	18229	16287	15618	18740	10968	13621	0.494907071	0.00685	1.41
RHOC	NM_001042679	2373	2407	3020	2839	1608	2240	0.496365693	0.00262	1.41
HIST1H2AH	NM_080596	3229	3409	4207	3683	2568	2885	0.496742502	0.01175	1.41
NUP54	NM_017426	301	382	438	393	240	338	0.496766906	0.02783	1.41
PHPT1	NM_014172	839	579	756	775	366	786	0.497020674	0.03256	1.41
RPLP1	NM_001003	7079	5397	5615	7574	2870	6031	0.497123522	0.0186	1.41
UBA52	NM_003333	2900	2415	3155	3226	1752	2319	0.497213505	0.00177	1.41
YTHDF2	NM_001173128	400	366	455	395	279	366	0.497765503	0.02367	1.41
XRCC6	NM_001469	7180	6126	6879	7467	4060	5937	0.497884166	0.00201	1.41
SRP72	NM_006947	4293	4067	3905	4389	2080	4442	0.498206995	0.01921	1.41
LAS1L	NM_031206	1780	1357	1540	2008	981	1152	0.498410366	0.00902	1.41
COPS3	NM_003653	675	612	836	777	450	600	0.499680663	0.00657	1.41
CLIC1	NM_001288	9498	8862	10479	10958	5569	8536	0.500002289	0.00066	1.41
LAMTOR1	NM_017907	1576	1287	1558	1633	827	1404	0.50052982	0.00466	1.41
NUDCD3	NM_015332	1183	1130	1243	1217	915	883	0.502409985	0.02227	1.42
OAZ1	NM_004152	3392	3129	3492	3661	2216	2728	0.502569422	0.00302	1.42
WBSCR22	NM_017528	843	801	837	893	482	798	0.503754648	0.00751	1.42
ARRB2	NM_004313	429	352	432	399	291	347	0.503871248	0.0253	1.42
ATXN7L3	NM_020218	329	309	375	341	242	286	0.503960517	0.02441	1.42
BANF1	NM_001143985	462	392	563	551	253	450	0.504207156	0.01117	1.42
COX6C	NM_004374	780	632	739	1059	355	583	0.505134036	0.0146	1.42
RNH1	NM_203384	916	729	788	1053	425	728	0.505553204	0.00875	1.42
SRA1	NM_001035235	668	562	588	826	321	513	0.505571557	0.01045	1.42
CCNB1	NM_031966	635	592	710	739	395	559	0.506189751	0.00369	1.42
INHBA	NM_002192	507	435	487	613	248	432	0.506659136	0.00917	1.42
HIST1H2BH	NM_003524	2374	1897	2015	3024	1130	1640	0.506842744	0.00939	1.42
C1QBP	NM_001212	2014	1749	2454	2131	1351	1830	0.508020739	0.00562	1.42
STRAP	NM_007178	276	284	350	354	189	260	0.509113364	0.01295	1.42
GALNT14	NM_024572	194	174	218	240	98	195	0.509385611	0.03284	1.42
RPS18	NM_022551	2661	2414	2189	2820	1250	2512	0.510337544	0.01411	1.42
C6orf48	NM_001040438	295	298	334	394	153	299	0.510839387	0.01742	1.42
RPS23	NM_001025	6840	5691	7661	7408	4423	5550	0.511321421	0.00218	1.43
C12orf43	NM_022895	177	158	209	230	93	173	0.511340088	0.03267	1.43
CENPO	NM_024322	360	316	368	427	171	352	0.512169268	0.01776	1.43
AIMP2	NM_006303	1560	1334	2036	1846	1141	1258	0.512724467	0.00865	1.43
CMC2	NM_020188	618	585	712	768	362	572	0.513260223	0.00287	1.43
TTF2	NM_003594	357	281	431	363	273	281	0.514591016	0.03376	1.43
EMC3	NM_018447	327	293	348	419	169	293	0.514623904	0.01132	1.43
DCXR	NM_016286	287	284	322	371	154	287	0.515589509	0.01448	1.43
PURB	NM_033224	194	234	328	289	172	207	0.516042551	0.03556	1.43

VEGFC	NM_005429	531	526	635	587	365	520	0.516483457	0.0083	1.43
UBIAD1	NM_013319	360	322	377	411	208	325	0.517636792	0.00826	1.43
WDR70	NM_018034	169	185	161	200	109	158	0.517985367	0.03836	1.43
CECR5	NM_033070	429	333	428	448	238	369	0.518128821	0.00987	1.43
CDK4	NM_000075	594	505	593	635	334	530	0.518554335	0.00544	1.43
TACO1	NM_016360	269	219	247	233	161	253	0.519227373	0.03886	1.43
MPI	NM_002435	180	140	169	173	119	141	0.520122482	0.04408	1.43
TSEN34	NM_001077446	290	175	276	317	160	193	0.520444585	0.03376	1.43
WDR12	NM_018256	384	386	422	363	331	325	0.521558887	0.04278	1.44
MRPS28	NM_014018	351	266	366	398	199	283	0.521894264	0.01045	1.44
EIF3K	NM_013234	2303	2172	2543	2722	1485	1962	0.522424842	0.00071	1.44
HIST1H2AG	NM_021064	5051	4296	5967	6543	3074	4036	0.522567918	0.00083	1.44
EBNA1BP2	NM_001159936	3440	3235	3651	4322	1982	2959	0.523619484	0.00035	1.44
CACYBP	NM_014412	634	542	444	768	299	460	0.524167927	0.02318	1.44
TGFBI	NM_000358	14805	14618	18872	18153	10467	13484	0.524762648	0.001	1.44
U2AF1	NM_006758	1091	1143	1027	1372	554	1089	0.525564355	0.00897	1.44
FADD	NM_003824	919	747	983	1194	563	637	0.525711681	0.00553	1.44
ORC1	NM_001190818	166	129	176	186	96	145	0.526415051	0.03158	1.44
RPL27A	NM_000990	6433	5759	7609	7729	4556	5018	0.526458368	0.00235	1.44
MRPS18B	NM_014046	1038	947	1210	1271	660	902	0.527534803	0.00104	1.44
ACAD9	NM_014049	224	239	306	286	165	234	0.528004695	0.01671	1.44
RNF26	NM_032015	1134	894	868	1169	617	826	0.5286025	0.00805	1.44
SWI5	NM_001040011	186	168	181	177	136	160	0.529278509	0.04217	1.44
AP2M1	NM_001025205	6538	6148	6594	7500	3856	5822	0.529873898	0.00049	1.44
SAP18	NM_005870	1041	937	1132	1194	676	875	0.529986861	0.00154	1.44
NEU1	NM_000434	165	150	207	191	138	133	0.530229828	0.04274	1.44
SNRPE	NM_003094	1637	1680	2105	2056	839	2090	0.530814365	0.01803	1.44
NDUFA3	NM_004542	415	319	382	498	192	346	0.531078675	0.01121	1.45
LSM2	NM_021177	772	796	824	858	507	755	0.531629965	0.00457	1.45
RPS6KA4	NM_003942	529	459	551	596	380	383	0.531660425	0.01099	1.45
COL7A1	NM_000094	189	159	200	208	140	141	0.532232822	0.03364	1.45
TMEM205	NM_001145416	548	431	440	545	297	438	0.532883392	0.01057	1.45
BABAM1	NM_001033549	698	601	671	721	429	596	0.533233188	0.00456	1.45
FOXRED1	NM_017547	199	159	214	235	108	181	0.533291728	0.02034	1.45
SCAMP3	NM_005698	368	322	545	464	294	329	0.533333809	0.01955	1.45
ACLY	NM_001096	2101	1968	2419	2579	1310	1892	0.533808802	0.00035	1.45
RNF149	NM_173647	160	147	157	187	95	144	0.534556805	0.02701	1.45
AATF	NM_012138	1469	1460	1712	1881	931	1347	0.534647384	0.00046	1.45
SFXN4	NM_213649	145	113	122	167	75	114	0.534772826	0.04252	1.45
RPL26L1	NM_016093	1563	1454	1803	1921	985	1391	0.534883593	0.00045	1.45
CSE1L	NM_001316	1740	1373	1755	2009	873	1550	0.535809239	0.00183	1.45
HIST1H2AE	NM_021052	1227	997	1146	1478	660	940	0.536003834	0.0016	1.45
MEMO1	NM_015955	579	523	589	612	344	555	0.53636443	0.00539	1.45
POLRMT	NM_005035	204	255	243	266	177	188	0.536365476	0.0291	1.45
EXOSC5	NM_020158	257	208	273	336	134	216	0.537084048	0.01296	1.45
MRPS23	NM_016070	307	226	300	328	166	262	0.537354758	0.01244	1.45

EXOSC7	NM_015004	248	222	247	324	137	205	0.537992293	0.01199	1.45
POLA2	NM_002689	307	274	346	367	200	267	0.538525856	0.00689	1.45
NDUFB8	NM_005004	1361	1240	1540	1583	812	1310	0.538879636	0.00094	1.45
DDX39A	NM_005804	886	678	953	883	653	653	0.538950903	0.0146	1.45
SLC35B1	NM_005827	354	373	417	473	206	373	0.539406366	0.00663	1.45
ABCD3	NM_002858	221	220	316	265	185	220	0.54108573	0.02345	1.46
MIIP	NM_021933	272	285	306	322	226	221	0.541623286	0.02119	1.46
UAP1	NM_003115	591	619	685	792	383	548	0.542366867	0.00161	1.46
NDUFAF6	NM_152416	150	156	171	184	87	172	0.5425298	0.03181	1.46
DGCR6L	NM_033257	382	344	374	497	211	313	0.54256851	0.00545	1.46
ANKRD13C	NM_030816	362	329	457	469	216	362	0.543058826	0.00551	1.46
TRAP1	NM_016292	1099	951	1201	1206	743	923	0.543101966	0.00217	1.46
UHMK1	NM_175866	665	573	654	752	407	544	0.543123779	0.00218	1.46
NAA20	NM_016100	622	469	615	735	323	509	0.543737687	0.00326	1.46
HDAC3	NM_003883	419	359	349	466	238	331	0.545299886	0.00813	1.46
OSGEP	NM_017807	199	174	191	184	146	173	0.545419814	0.03562	1.46
CDKN3	NM_005192	1782	1800	1937	1920	964	2167	0.546201139	0.01285	1.46
COX4I1	NM_001861	1677	1859	2196	2018	1333	1695	0.546409099	0.00367	1.46
TMEM237	NM_001044385	196	195	280	285	103	247	0.546555347	0.03174	1.46
POLR2H	NM_006232	312	318	347	328	192	367	0.546943083	0.01982	1.46
HMBS	NM_000190	244	201	194	286	120	198	0.547033504	0.0203	1.46
CDC45	NM_003504	525	454	605	620	346	455	0.547169794	0.00267	1.46
NSFL1C	NM_016143	828	787	887	907	593	716	0.547265944	0.00367	1.46
DDX55	NM_020936	618	633	672	638	515	531	0.547475751	0.01649	1.46
RPL7A	NM_000972	4019	3701	4040	4412	2017	4402	0.547735492	0.00649	1.46
CHPT1	NM_020244	152	127	248	209	131	138	0.548294858	0.04654	1.46
RFT1	NM_052859	804	846	786	1055	572	604	0.548640394	0.00653	1.46
ARHGAP18	NM_033515	442	352	410	468	302	311	0.548884693	0.01195	1.46
HAUS7	NM_017518	264	243	258	334	153	224	0.549555174	0.00831	1.46
SMARCE1	NM_003079	1201	667	1313	1106	789	907	0.549759466	0.02582	1.46
CISD2	NM_001008388	406	428	431	479	307	352	0.551169567	0.00799	1.47
ASB1	NM_001040445	580	523	583	604	356	558	0.551398583	0.00467	1.47
GMPR2	NM_001002002	236	243	292	311	155	242	0.551562779	0.00805	1.47
PPP1CA	NM_002708	4672	3683	4813	5076	2924	3743	0.551702356	0.00078	1.47
C12orf57	NM_138425	1191	898	1265	1181	767	1022	0.553057959	0.0043	1.47
ORMDL2	NM_014182	116	86	139	149	64	110	0.553156142	0.04231	1.47
GFER	NM_005262	200	205	191	216	142	186	0.553682786	0.02229	1.47
UBXN2A	NM_181713	107	134	142	155	104	95	0.553805397	0.04943	1.47
NOLC1	NM_004741	1561	1477	1692	1903	968	1420	0.554635688	0.00029	1.47
TRIAP1	NM_016399	285	300	362	389	188	295	0.555175706	0.00522	1.47
C8orf76	NM_032847	263	228	279	324	166	219	0.556094365	0.00683	1.47
PET100	NM_001171155	125	146	144	193	70	139	0.556470323	0.0337	1.47
PSMB6	NM_002798	1857	1478	1728	1629	1274	1545	0.556647808	0.00978	1.47
MRPS5	NM_031902	280	225	286	318	147	270	0.557469582	0.0101	1.47
AKAP8	NM_005858	683	702	739	819	523	568	0.557665283	0.00451	1.47
BAG6	NM_001098534	740	642	721	754	556	558	0.558203299	0.01009	1.47

SNRPD2	NM_004597	1531	1401	1579	1740	925	1426	0.558216487	0.00056	1.47
SDHD	NM_003002	640	686	731	844	449	587	0.560031583	0.00136	1.47
MTMR14	NM_001077525	159	121	162	178	76	166	0.56041823	0.03373	1.47
THOC6	NM_024339	438	392	439	534	329	297	0.561639209	0.01202	1.48
TXNDC17	NM_032731	271	190	241	296	138	224	0.562242192	0.01248	1.48
COA1	NM_018224	743	684	826	868	557	601	0.565024131	0.00317	1.48
RANGAP1	NM_002883	718	561	671	637	631	445	0.565058667	0.04389	1.48
AMDHD2	NM_001145815	94	85	108	128	68	77	0.565162105	0.04217	1.48
HIST2H2BE	NM_003528	5979	4803	5804	6689	3324	5176	0.565167848	0.00028	1.48
RPL23A	NM_000984	356	328	259	438	209	255	0.565269436	0.01713	1.48
TSN	NM_004622	512	405	512	561	337	400	0.565975824	0.00383	1.48
SLC35A5	NM_017945	165	179	186	247	72	209	0.566228681	0.04179	1.48
TAGLN2	NM_003564	5732	5250	6773	6766	3760	5490	0.566834125	0.00023	1.48
OIP5	NM_007280	214	191	231	277	103	231	0.567736717	0.01688	1.48
ARL16	NM_001040025	124	137	138	188	72	128	0.568613167	0.02561	1.48
MED10	NM_032286	211	230	327	258	182	255	0.568641033	0.02064	1.48
POLDIP2	NM_015584	700	691	870	721	603	670	0.56923616	0.01202	1.48
EMP3	NM_001425	2841	2495	3074	2689	2205	2498	0.569421338	0.00764	1.48
UBE2D3	NM_181891	3008	3065	3402	3263	2482	2651	0.569469707	0.00521	1.48
POLD4	NM_021173	434	336	479	417	293	413	0.570337508	0.01023	1.48
NRAS	NM_002524	1101	1129	1540	1362	844	1184	0.570409585	0.00229	1.48
DKC1	NM_001363	857	752	991	997	574	786	0.570931262	0.00087	1.49
RPL27	NM_000988	13162	12429	15143	16840	7972	12716	0.571486601	5.62E-05	1.49
LRRC14	NM_014665	239	200	227	215	207	175	0.571623554	0.04228	1.49
HSPA8	NM_006597	10810	10776	12559	12215	7306	11317	0.571948625	0.00072	1.49
BYSL	NM_004053	255	212	314	369	181	186	0.57219353	0.01298	1.49
ATPIF1	NM_016311	234	241	203	361	108	215	0.574007933	0.02445	1.49
MMS19	NM_022362	343	260	350	384	228	264	0.574126965	0.00652	1.49
IMPDH2	NM_000884	1712	1455	2041	1883	1370	1381	0.574595346	0.00539	1.49
EFR3A	NM_015137	345	342	452	343	280	398	0.574943659	0.01883	1.49
ADAMTS1	NM_006988	143	132	211	189	94	175	0.575145864	0.02476	1.49
ATP5G2	NM_005176	2241	1600	2056	2199	1448	1679	0.57677837	0.00321	1.49
TUBA3C	NM_006001	1332	1163	1332	1613	757	1196	0.577193288	0.00027	1.49
DNPEP	NM_012100	140	137	158	195	91	130	0.578919977	0.01459	1.49
CCT5	NM_012073	4716	4195	5560	5611	3607	3820	0.578978987	0.00142	1.49
CCT2	NM_006431	749	693	705	978	370	723	0.579349684	0.0027	1.49
WBSCR16	NM_030798	233	180	225	279	119	212	0.57938616	0.00951	1.49
SMAD5	NM_001001420	273	242	266	390	132	245	0.580199124	0.00897	1.50
ARFIP1	NM_001025595	101	85	93	108	64	95	0.580600677	0.04013	1.50
GTF2A2	NM_004492	411	402	539	606	244	437	0.582146699	0.00239	1.50
PHB	NM_002634	989	888	970	1118	732	749	0.582326199	0.0032	1.50
SCP2	NM_002979	384	416	466	414	299	440	0.582409739	0.00963	1.50
PPP4C	NM_002720	1039	939	1113	1194	677	965	0.583185905	0.00043	1.50
CEP97	NM_024548	138	123	196	186	102	142	0.583257743	0.01849	1.50
PLIN3	NM_005817	116	116	126	167	93	85	0.583276395	0.03259	1.50
PDXK	NM_003681	382	351	355	448	260	307	0.58462833	0.00402	1.50

DRG1	NM_004147	846	765	893	967	594	733	0.587330784	0.0009	1.50
HSPBP1	NM_001130106	684	492	651	745	441	503	0.58762412	0.00298	1.50
DLG4	NM_001128827	127	125	131	140	119	98	0.587798717	0.04274	1.50
LDLR	NM_000527	1063	1046	1170	1306	754	960	0.588040488	0.00041	1.50
HNRNPA1	NM_031157	924	1096	922	1038	718	960	0.589450459	0.00648	1.50
ERCC2	NM_000400	257	200	271	305	187	188	0.589484968	0.01018	1.50
APOOL	NM_198450	135	108	171	150	126	107	0.591665856	0.04068	1.51
H2AFV	NM_012412	5131	3997	5323	5582	3457	4156	0.593005583	0.00059	1.51
C6orf1	NM_001008704	242	263	259	294	204	214	0.594068814	0.01053	1.51
JMJD6	NM_001081461	135	141	195	176	107	161	0.594631386	0.01695	1.51
PDXP	NM_020315	318	257	380	322	271	273	0.595454165	0.01591	1.51
HIST1H3A	NM_003529	2578	1953	2206	3081	1161	2337	0.595965429	0.0021	1.51
NCAPH	NM_015341	272	287	345	376	227	243	0.596200283	0.00508	1.51
ATAD3A	NM_001170535	574	463	399	474	344	529	0.597013534	0.01782	1.51
TIA1	NM_022173	302	271	399	297	217	385	0.597470724	0.01893	1.51
DPH1	NM_001383	209	260	235	275	182	207	0.597654519	0.01186	1.51
ILK	NM_004517	407	443	487	482	324	430	0.598956099	0.00355	1.51
NRG1	NM_013956	207	186	155	284	107	162	0.598985556	0.0175	1.51
RAB32	NM_006834	162	177	183	187	133	170	0.599015689	0.01508	1.51
BCAR3	NM_003567	314	278	380	397	208	310	0.599043558	0.00207	1.51
RPL7	NM_000971	10683	10982	13068	12536	8402	10697	0.599394674	0.00066	1.52
FAM207A	NM_058190	639	584	780	866	406	622	0.599982502	0.00033	1.52
FKBP4	NM_002014	2383	2304	2609	2976	1732	2056	0.600627852	0.00023	1.52
SLC39A3	NM_144564	253	206	254	317	152	215	0.600918617	0.00364	1.52
MCAM	NM_006500	640	601	585	950	385	468	0.601642749	0.0031	1.52
SLC25A32	NM_030780	997	964	927	1096	824	744	0.601821407	0.00775	1.52
ATG4B	NM_013325	426	340	428	433	300	370	0.602336022	0.0043	1.52
EIF3L	NM_016091	2926	2395	2616	3094	2107	2119	0.602432078	0.00266	1.52
NPM1	NM_002520	19436	19075	21165	21319	14068	19352	0.603631518	0.00053	1.52
COG4	NM_015386	603	600	751	812	399	637	0.604758545	0.0004	1.52
RPLP0	NM_001002	14216	11861	13185	16483	7790	13075	0.60538772	0.00014	1.52
ANAPC15	NM_014042	460	490	394	700	295	348	0.606327557	0.00712	1.52
RPL11	NM_000975	36792	37344	39995	42126	32388	29679	0.606688192	0.00422	1.52
SAFB2	NM_014649	166	163	174	253	72	193	0.607172979	0.02261	1.52
LY6K	NM_017527	369	250	269	366	224	259	0.608998488	0.00995	1.53
RHBDD3	NM_012265	148	113	122	147	113	106	0.609130817	0.02994	1.53
MRPL27	NM_016504	129	110	139	185	88	100	0.609307285	0.01591	1.53
RPL39L	NM_052969	181	100	134	179	64	185	0.612227189	0.04627	1.53
NUPL1	NM_014089	388	424	530	567	326	375	0.61241904	0.00178	1.53
NAA38	NM_032356	687	759	823	974	554	619	0.612696556	0.00087	1.53
STX4	NM_004604	445	426	460	510	388	341	0.612969177	0.00832	1.53
OGFOD3	NM_024648	191	188	321	254	226	169	0.613275796	0.03549	1.53
MRPL33	NM_004891	138	134	159	185	70	178	0.613287413	0.02118	1.53
HIST1H1D	NM_005320	4507	3671	4555	6041	2290	4157	0.614031404	0.00024	1.53
MRPL21	NM_181514	1320	1180	1357	1604	846	1195	0.614097647	7.89E-05	1.53
QTRT1	NM_031209	104	94	109	118	84	94	0.614101778	0.02501	1.53

CASP3	NM_032991	279	269	393	310	267	288	0.614981961	0.01353	1.53
HRAS	NM_001130442	85	60	79	97	57	69	0.616888386	0.03778	1.53
BAIAP2	NM_017451	785	541	715	861	525	546	0.618449044	0.00288	1.54
BCL7C	NM_004765	441	462	425	544	289	447	0.61860916	0.00155	1.54
OGG1	NM_016820	255	197	258	236	234	187	0.619147685	0.02737	1.54
DDX3X	NM_001356	1730	2044	1944	2688	1150	1774	0.620465161	0.00028	1.54
FRMD5	NM_032892	136	138	157	209	91	129	0.620628845	0.00831	1.54
ADSL	NM_000026	1585	1565	1660	2204	1177	1234	0.620813069	0.00056	1.54
SUPT3H	NM_003599	150	162	153	157	136	149	0.621377768	0.02226	1.54
EIF4H	NM_022170	5855	4732	6592	6371	5045	4341	0.621454528	0.00542	1.54
MTOR	NM_004958	369	298	378	466	205	348	0.621535954	0.00117	1.54
TRIM23	NM_001656	59	62	88	99	54	58	0.621897628	0.04144	1.54
HSPA14	NM_016299	143	147	164	220	108	122	0.623724324	0.00888	1.54
SNRPG	NM_003096	158	140	169	196	102	158	0.623840528	0.00663	1.54
ZNF593	NM_015871	372	351	353	366	319	319	0.624412127	0.01084	1.54
MRPL2	NM_015950	411	299	301	396	255	321	0.625492691	0.00654	1.54
PPP1R14B	NM_138689	3536	3027	3263	4311	2298	2796	0.625889001	0.00013	1.54
WDR77	NM_024102	638	541	700	699	491	563	0.626084169	0.00163	1.54
IRF3	NM_001571	425	313	387	418	323	315	0.626098102	0.00785	1.54
RRP9	NM_004704	282	216	201	293	174	216	0.626338286	0.00867	1.54
NANS	NM_018946	624	548	621	746	451	507	0.626844517	0.00077	1.54
SIVA1	NM_006427	533	502	461	642	386	414	0.62717135	0.00252	1.54
DHPS	NM_001930	628	468	552	631	464	455	0.627323023	0.00522	1.54
RPS28	NM_001031	3965	3037	3968	5150	2627	2814	0.628992873	0.00045	1.55
ATG101	NM_001098673	135	115	103	153	110	87	0.629576024	0.03246	1.55
PSMG4	NM_001128592	337	173	267	322	211	220	0.629853527	0.01628	1.55
EIF4A3	NM_014740	344	300	387	420	287	271	0.630046566	0.00436	1.55
UBA3	NM_003968	534	547	425	570	330	575	0.630103438	0.00628	1.55
NAA10	NM_003491	346	276	305	368	253	264	0.630653278	0.00504	1.55
ZNF511	NM_145806	292	255	351	280	275	276	0.630820544	0.01591	1.55
PFDN5	NM_002624	719	590	475	757	339	705	0.631901271	0.00828	1.55
CUTA	NM_001014433	425	362	448	531	297	359	0.632656567	0.00066	1.55
PLEKHA2	NM_021623	1024	900	1203	1049	936	889	0.632701154	0.00654	1.55
ORC6	NM_014321	311	278	368	423	212	294	0.633279713	0.00075	1.55
PRMT1	NM_001536	2908	2329	2653	3230	1878	2402	0.633382582	0.00017	1.55
TRAPPC2L	NM_016209	735	599	800	911	470	669	0.633539625	0.00015	1.55
SSRP1	NM_003146	1668	1571	1983	1695	1555	1540	0.634454239	0.00598	1.55
WDR34	NM_052844	479	467	498	501	430	419	0.634503868	0.00694	1.55
TAF1A	NM_001201536	98	88	122	128	82	93	0.635317111	0.0175	1.55
FARSA	NM_004461	2939	2533	3318	3441	2246	2556	0.635839509	0.0003	1.55
PTPRN	NM_002846	56	74	78	103	48	65	0.636030884	0.03421	1.55
MRPL14	NM_032111	363	357	353	420	270	342	0.638275247	0.00183	1.56
PARP2	NM_005484	124	100	78	160	61	100	0.639075289	0.02809	1.56
METTL13	NM_015935	123	98	166	144	105	126	0.640624777	0.01722	1.56
CDC6	NM_001254	431	359	326	470	283	339	0.640934177	0.00332	1.56
UQCC2	NM_032340	661	542	671	818	369	655	0.641305834	0.0003	1.56

ZNF576	NM_001145347	145	104	152	182	115	100	0.641667867	0.01683	1.56
UNC45A	NM_018671	436	335	581	571	330	400	0.643128139	0.00195	1.56
GEMIN6	NM_024775	175	184	183	216	130	187	0.644888238	0.00487	1.56
C7orf55	NM_197964	396	347	372	454	293	329	0.644985679	0.00163	1.56
JMJD4	NM_023007	104	72	91	118	69	83	0.645058114	0.02102	1.56
FDX1L	NM_001031734	256	182	223	259	195	185	0.646170192	0.00995	1.57
HYPK	NM_016400	328	307	370	427	198	370	0.647365922	0.00101	1.57
SART3	NM_014706	498	429	608	594	425	437	0.647903466	0.00214	1.57
MANF	NM_006010	1710	1365	1531	1917	1019	1546	0.649159645	0.00012	1.57
PRKCDBP	NM_145040	854	735	858	1092	631	653	0.64927885	0.00049	1.57
GEMIN2	NM_003616	431	402	405	510	297	400	0.649305507	0.00068	1.57
FBL	NM_001436	1376	946	1133	1631	760	1044	0.649548271	0.00043	1.57
FIS1	NM_016068	334	268	260	433	182	265	0.649689858	0.00256	1.57
SLC16A3	NM_001206950	129	128	142	178	83	143	0.651134451	0.00652	1.57
WDR61	NM_025234	158	133	137	190	107	132	0.651145209	0.00692	1.57
WDR83OS	NM_016145	1838	1525	1731	2035	1351	1480	0.651154742	0.00047	1.57
EP300	NM_001429	176	176	72	238	85	158	0.651315769	0.04946	1.57
CCDC51	NM_024661	109	109	165	142	114	117	0.651995382	0.0181	1.57
NIP7	NM_016101	1282	997	1053	1391	749	1126	0.652265448	0.00035	1.57
DCTPP1	NM_024096	1151	1108	1170	1422	718	1226	0.653219888	0.00012	1.57
HSD17B7	NM_016371	78	78	81	103	54	87	0.653293314	0.01997	1.57
AURKA	NM_003600	101	87	143	147	90	94	0.653493411	0.01539	1.57
DNPH1	NM_006443	81	81	152	106	91	112	0.654414892	0.03421	1.57
ATOX1	NM_004045	725	631	781	946	537	595	0.654482297	0.00025	1.57
LMAN2	NM_006816	1717	1685	2020	2163	1350	1673	0.655083666	8.66E-05	1.57
EIF3J	NM_003758	290	244	243	380	168	243	0.65787056	0.00168	1.58
MRPL4	NM_015956	182	154	187	192	143	175	0.658082426	0.00657	1.58
CSTF2	NM_001325	312	271	310	304	304	242	0.658308926	0.01486	1.58
ADORA2B	NM_000676	96	70	73	128	58	68	0.658327143	0.02445	1.58
CD151	NM_001039490	2112	1632	2075	2088	1829	1562	0.658541269	0.00515	1.58
FAM175B	NM_032182	127	141	81	200	72	114	0.660903238	0.02633	1.58
RABAC1	NM_006423	1124	895	1317	1133	925	1088	0.661311251	0.00229	1.58
GIPC1	NM_202470	798	883	977	883	750	889	0.661398346	0.00295	1.58
PMVK	NM_006556	181	194	208	284	137	170	0.66200068	0.00218	1.58
LAMTOR4	NM_001008395	126	76	111	150	75	98	0.662052577	0.01433	1.58
PSMG1	NM_003720	800	623	907	819	595	812	0.663284078	0.00135	1.58
CHST11	NM_018413	83	65	80	109	54	75	0.664039358	0.01829	1.58
FKBP2	NM_004470	619	694	739	762	562	650	0.664758755	0.0012	1.59
NDUFB9	NM_005005	1196	985	1166	1408	790	1072	0.665575019	5.03E-05	1.59
SSR4	NM_001204527	720	628	581	974	419	584	0.665769573	0.00051	1.59
EGLN2	NM_053046	351	359	383	351	360	331	0.667022675	0.01073	1.59
SNF8	NM_007241	467	414	481	575	283	500	0.66716359	0.00032	1.59
S100A16	NM_080388	4148	3398	3712	4424	3003	3436	0.667512195	0.00035	1.59
CLDN12	NM_012129	43	56	77	80	37	72	0.667965067	0.03775	1.59
PALB2	NM_024675	110	95	138	133	81	132	0.669048067	0.01055	1.59
NRIP3	NM_020645	279	214	242	354	153	246	0.669238032	0.00138	1.59

NHP2	NM_017838	758	717	691	931	562	643	0.66934496	0.0004	1.59
NUDT6	NM_007083	75	49	67	90	45	68	0.671689211	0.02623	1.59
DHX16	NM_003587	353	330	439	507	251	361	0.672150686	0.0002	1.59
HEXB	NM_000521	158	134	150	213	92	153	0.67422924	0.00343	1.60
DCAF15	NM_138353	466	400	597	543	443	418	0.674319268	0.00364	1.60
DHX35	NM_021931	210	219	172	269	165	179	0.675393353	0.00547	1.60
CDK14	NM_012395	165	156	243	219	141	201	0.678573044	0.0043	1.60
IFRD2	NM_006764	537	481	574	714	351	529	0.678893543	4.58E-05	1.60
RPS3	NM_001005	12583	11172	11059	14610	6937	13758	0.678898113	0.00028	1.60
RUFY1	NM_025158	365	316	193	356	279	262	0.67959341	0.01854	1.60
ABCF3	NM_018358	331	321	239	426	206	296	0.679674743	0.00275	1.60
HNRNPD	NM_031370	696	684	892	929	525	784	0.680133105	8.05E-05	1.60
NTMT1	NM_014064	159	115	99	167	88	138	0.680472361	0.0125	1.60
C1orf52	NM_198077	114	110	116	150	88	111	0.681193028	0.00628	1.60
PCNA	NM_182649	859	804	947	1122	582	892	0.681733834	1.70E-05	1.60
ALG5	NM_013338	140	171	109	227	86	148	0.682500462	0.01223	1.60
RANBP17	NM_022897	168	164	193	247	74	254	0.683993573	0.01533	1.61
LRRC58	NM_001099678	279	283	374	395	216	326	0.68461272	0.00046	1.61
OXLD1	NM_001039842	52	48	67	78	61	39	0.684879116	0.04274	1.61
RPL18	NM_000979	10241	10226	11569	12783	7777	10809	0.685620127	1.34E-05	1.61
RPL37A	NM_000998	6435	4907	5594	7624	4179	5093	0.685723046	4.86E-05	1.61
PAGR1	NM_024516	388	314	367	493	277	307	0.685924049	0.00051	1.61
HIST1H2BG	NM_003518	703	615	680	896	488	615	0.685989371	5.72E-05	1.61
BAP1	NM_004656	1163	935	1205	1483	750	1069	0.687054144	1.09E-05	1.61
DUSP3	NM_004090	130	136	208	192	142	141	0.689227058	0.00804	1.61
POLR2G	NM_002696	305	261	328	360	242	285	0.690438171	0.00082	1.61
FAM118A	NM_017911	61	42	56	51	52	65	0.692014714	0.04274	1.62
FAH	NM_000137	38	46	34	68	22	50	0.692022876	0.04923	1.62
UQCRH	NM_006004	796	661	747	1010	550	658	0.692161312	7.10E-05	1.62
CCT7	NM_006429	1419	987	1309	1511	940	1228	0.693265686	0.00021	1.62
RUVBL2	NM_006666	662	531	681	848	441	597	0.694012655	3.51E-05	1.62
TJP2	NM_001170416	242	234	303	288	230	250	0.694033637	0.00272	1.62
NME3	NM_002513	87	62	71	100	73	60	0.695579501	0.02345	1.62
MED27	NM_004269	175	154	162	221	133	150	0.695868447	0.00247	1.62
SKA3	NM_145061	197	235	247	251	142	311	0.696842861	0.00598	1.62
TK1	NM_003258	1661	1326	1731	1709	1395	1472	0.697298437	0.00091	1.62
PARL	NM_018622	182	147	166	199	158	143	0.697681391	0.00677	1.62
RPS16	NM_001020	12417	11577	13750	15583	10220	11276	0.697992633	4.27E-05	1.62
PHB2	NM_001144831	1998	1827	1828	2232	1438	1955	0.69930037	0.00011	1.62
PTTG1	NM_004219	313	280	332	405	203	338	0.69997388	0.00024	1.62
R3HCC1	NM_001136108	146	133	141	209	88	149	0.70015882	0.00246	1.62
HIST3H2A	NM_033445	408	322	415	559	247	377	0.700548338	0.00012	1.63
POLR1E	NM_022490	637	569	540	797	372	641	0.700987158	0.00021	1.63
CKS1B	NM_001826	1327	1061	976	1810	597	1261	0.701138671	0.00118	1.63
SNRPB	NM_198216	3846	4167	4090	5620	3194	3424	0.701350476	6.56E-05	1.63
C4orf22	NM_001206997	81	91	101	124	63	103	0.701567399	0.00792	1.63

DGKH	NM_178009	45	35	42	53	36	46	0.701587941	0.03828	1.63
FAM98C	NM_174905	73	65	64	86	52	79	0.70220062	0.01703	1.63
EIF3G	NM_003755	842	608	728	860	641	662	0.704424187	0.00107	1.63
ESRRA	NM_004451	252	290	295	391	206	268	0.708256655	0.00033	1.63
LYSMD1	NM_212551	75	81	93	102	88	70	0.708459507	0.01849	1.63
FDPS	NM_002004	60	53	78	63	43	103	0.70880359	0.03453	1.63
HIST1H2BN	NM_003520	2024	1928	1864	2389	1509	1948	0.709641635	6.40E-05	1.64
HSP90B1	NM_003299	8464	8158	8300	10195	5579	9548	0.710062312	3.00E-05	1.64
FAU	NM_001997	1821	1682	1882	2010	1581	1709	0.710582139	0.0004	1.64
HSPE1	NM_002157	1342	1099	1231	1735	829	1214	0.710648713	7.31E-06	1.64
HYAL2	NM_003773	134	152	141	193	108	148	0.71277396	0.00238	1.64
GLIPR2	NM_022343	170	158	160	227	123	161	0.712986733	0.0013	1.64
PSMD13	NM_002817	350	311	380	453	220	406	0.714822441	0.00023	1.64
GADD45B	NM_015675	232	198	300	313	208	220	0.71520904	0.00125	1.64
NUP37	NM_024057	113	105	129	198	67	118	0.71523042	0.00425	1.64
DYNLL2	NM_080677	268	233	296	325	214	269	0.715627648	0.0005	1.64
HIST1H3B	NM_003537	8499	7233	9260	12784	5333	7910	0.715972234	6.85E-07	1.64
TSSC4	NM_005706	175	141	190	239	116	176	0.71638295	0.0009	1.64
RNF181	NM_016494	205	168	155	214	124	220	0.716998021	0.00385	1.64
PSMC4	NM_006503	1676	1433	2024	1980	1287	1849	0.717261207	6.77E-05	1.64
ABT1	NM_013375	208	160	215	276	146	186	0.718781827	0.00068	1.65
RPS5	NM_001009	3627	2868	3429	4114	2580	3274	0.719125358	2.31E-05	1.65
RUVBL1	NM_003707	854	662	847	948	692	721	0.719186643	0.0004	1.65
FRA10AC1	NM_145246	82	68	63	103	43	94	0.720812181	0.01533	1.65
CHCHD6	NM_032343	63	50	42	71	45	58	0.721196714	0.0251	1.65
MRPL48	NM_016055	89	96	92	146	69	88	0.721503751	0.00576	1.65
TATDN1	NM_032026	98	100	117	133	89	109	0.723122409	0.00468	1.65
KRT18	NM_000224	91	87	102	150	67	89	0.723790699	0.00474	1.65
PPAPDC1A	NM_001030059	34	29	40	56	32	32	0.724556197	0.03799	1.65
EIF2D	NM_006893	66	72	79	87	66	79	0.725148892	0.01255	1.65
AUP1	NM_181575	359	282	348	511	237	297	0.7258816	0.00016	1.65
BAG1	NM_001172415	73	71	71	111	50	78	0.726230396	0.00903	1.65
NDUFB4	NM_004547	1907	1702	1883	2402	1312	1903	0.726397301	2.15E-06	1.65
HSP90AB1	NM_007355	3846	3106	4914	5335	2621	4234	0.727383091	9.71E-06	1.66
SUPT16H	NM_007192	512	470	595	557	481	534	0.728177575	0.00107	1.66
PRMT5	NM_006109	1166	1219	1386	1571	1001	1242	0.728579021	2.15E-05	1.66
FKBP11	NM_016594	70	82	83	118	57	85	0.734206138	0.00675	1.66
HIST2H2AB	NM_175065	1315	1203	1435	1897	915	1295	0.73421241	3.68E-07	1.66
PRELID1	NM_013237	1593	1429	1707	2133	938	1927	0.735458552	3.42E-05	1.66
CTNNBL1	NM_030877	476	401	399	521	307	506	0.735699904	0.00035	1.67
CAMTA1	NM_015215	57	48	57	67	45	68	0.737572936	0.01731	1.67
LUC7L3	NM_016424	428	347	386	453	281	477	0.74016029	0.00046	1.67
SRPX	NM_006307	300	365	394	442	324	325	0.740522425	0.00082	1.67
PSMA7	NM_002792	3544	2987	3344	3649	2753	3597	0.743274573	0.00015	1.67
MRPL54	NM_172251	129	93	128	188	89	107	0.743692095	0.00249	1.67
RPS19	NM_001022	11878	11182	11040	16521	7749	11805	0.745209212	6.26E-07	1.68

DUS3L	NM_020175	144	120	122	162	108	143	0.747288611	0.00252	1.68
PTGES2	NM_025072	257	213	331	340	220	271	0.747385217	0.0004	1.68
RECK	NM_021111	41	26	47	49	39	43	0.748817042	0.03079	1.68
C14orf2	NM_004894	1806	1514	2003	2132	1524	1742	0.749101043	5.17E-05	1.68
KIAA0101	NM_014736	968	1089	1070	1395	848	1014	0.75116234	2.17E-05	1.68
TMEM160	NM_017854	265	217	286	324	216	260	0.751625794	0.00031	1.68
KRTCAP2	NM_173852	855	593	882	1034	658	718	0.751645582	0.00012	1.68
PAPD5	NM_001040284	113	108	153	173	110	117	0.751732644	0.00222	1.68
GRB14	NM_004490	39	33	37	53	36	39	0.755764508	0.02558	1.69
UROD	NM_000374	735	631	807	1051	483	777	0.757309969	1.54E-06	1.69
AP2A1	NM_014203	663	621	554	856	669	432	0.758725142	0.00373	1.69
TIMM10	NM_012456	539	521	449	731	349	563	0.761418787	7.91E-05	1.70
EPHA2	NM_004431	939	873	1088	1011	985	940	0.761725132	0.00113	1.70
LSP1	NM_001013253	28	27	25	43	24	33	0.761974141	0.03549	1.70
CKS2	NM_001827	326	297	395	462	206	437	0.762591465	0.00022	1.70
GSTO1	NM_004832	157	117	140	184	121	141	0.762992639	0.00149	1.70
CHCHD10	NM_213720	331	281	412	492	246	354	0.763290532	3.21E-05	1.70
EMC8	NM_006067	323	276	260	363	271	277	0.765739164	0.0009	1.70
ROMO1	NM_080748	1046	780	934	1393	565	1074	0.769191801	2.30E-05	1.70
C14orf80	NM_001134875	101	95	108	150	62	133	0.769332427	0.00266	1.70
PITRM1	NM_001242307	164	137	154	233	104	168	0.770065415	0.00045	1.71
LZIC	NM_032368	113	128	108	171	81	142	0.771136551	0.00194	1.71
SF3B5	NM_031287	825	725	758	1037	502	966	0.772973274	3.42E-05	1.71
RPS14	NM_001025070	476	361	295	722	201	441	0.773197379	0.00235	1.71
TMED9	NM_017510	1620	1272	1665	2014	1255	1495	0.773580421	5.50E-06	1.71
ATXN10	NM_013236	584	654	663	1095	352	730	0.777689854	9.22E-05	1.71
PSMD3	NM_002809	870	631	842	968	704	778	0.779339743	0.00011	1.72
OTUD3	NM_015207	78	67	59	93	59	82	0.780920296	0.00698	1.72
RPP21	NM_001199120	97	90	128	176	80	101	0.781352876	0.00151	1.72
HIST1H2AK	NM_003510	244	245	220	338	193	245	0.781770228	0.00018	1.72
SCO1	NM_004589	110	103	122	152	87	132	0.782758189	0.00109	1.72
CENPH	NM_022909	63	74	66	95	57	81	0.782769884	0.00588	1.72
DIO2	NM_013989	18	16	31	36	22	27	0.78441187	0.03886	1.72
CRLS1	NM_019095	192	186	232	254	142	272	0.78456814	0.00049	1.72
COX5B	NM_001862	899	752	993	1194	748	850	0.784965011	6.77E-06	1.72
MRPL24	NM_145729	1034	660	695	1252	562	870	0.787008893	0.00016	1.73
ATP5E	NM_006886	536	622	727	831	382	884	0.789929414	0.00024	1.73
MAGOHB	NM_018048	2065	1739	1545	2073	1485	2192	0.790734163	0.00023	1.73
MKKS	NM_170784	68	58	76	92	48	94	0.790800588	0.00576	1.73
DAPK3	NM_001348	353	295	360	394	377	294	0.791630643	0.00184	1.73
KIAA2013	NM_138346	128	157	170	210	148	142	0.792949879	0.00118	1.73
RPL35	NM_007209	7332	6658	7466	8884	5308	8742	0.794649284	2.53E-06	1.73
EMG1	NM_006331	677	579	732	834	551	726	0.795455007	1.03E-05	1.74
IGFBP6	NM_002178	239	179	188	291	180	199	0.795621733	0.00042	1.74
RPS21	NM_001024	5098	5400	5295	6550	5007	5156	0.798862329	5.17E-05	1.74
NDUFA13	NM_015965	1514	1326	1454	1868	1250	1475	0.805498165	4.96E-06	1.75

DACT3	NM_145056	17	35	40	45	36	36	0.805898031	0.02672	1.75
EIF6	NM_181468	1948	1618	2309	2559	1947	1721	0.806627037	0.00012	1.75
HIST1H2AB	NM_003513	898	860	853	1355	675	861	0.807158499	8.03E-07	1.75
COX6B1	NM_001863	2294	1955	2000	2757	1710	2291	0.80796762	2.77E-06	1.75
EBP	NM_006579	293	248	298	349	192	396	0.808913199	0.00028	1.75
CRABP2	NM_001878	337	310	305	374	312	344	0.809837203	0.0004	1.75
SSNA1	NM_003731	278	253	291	438	189	301	0.810680225	1.03E-05	1.75
OTOGL	NM_173591	10	15	23	26	17	26	0.811194851	0.04042	1.75
NTHL1	NM_002528	103	79	83	147	75	89	0.813405589	0.00181	1.76
PAAF1	NM_025155	260	140	137	304	119	229	0.813410326	0.00277	1.76
URM1	NM_001135947	157	146	178	304	100	167	0.814017072	0.00032	1.76
HIST1H3G	NM_003534	4515	3985	4223	6057	3117	4802	0.81437107	5.44E-08	1.76
RAB34	NM_001144943	754	763	777	923	690	856	0.81462959	3.61E-05	1.76
BOP1	NM_015201	371	298	311	487	308	295	0.815359079	0.00016	1.76
DKK1	NM_012242	61	59	71	114	40	80	0.817166105	0.00384	1.76
RPS3A	NM_001006	4770	4791	4482	7541	2791	5963	0.818644042	9.17E-06	1.76
UBE2S	NM_014501	3379	2654	3119	4455	2160	3533	0.819192372	1.27E-07	1.76
BRMS1	NM_001024957	607	612	361	794	427	629	0.822048797	0.00064	1.77
BCL2L12	NM_138639	822	686	730	895	766	754	0.823555043	0.00021	1.77
DPH5	NM_001077394	101	88	93	140	80	107	0.825299382	0.0009	1.77
CHCHD2	NM_016139	2954	2586	2879	3745	2233	3214	0.826412471	2.00E-07	1.77
NEDD8	NM_006156	381	334	622	484	634	616	0.826704349	0.03373	1.77
PSMD14	NM_005805	746	930	1071	1342	630	1126	0.826770307	4.56E-06	1.77
COL17A1	NM_000494	25	34	48	42	40	51	0.828156057	0.018	1.78
HIST1H4C	NM_003542	7431	7443	7468	11272	6082	7404	0.828346998	5.33E-08	1.78
MRTO4	NM_016183	715	601	660	813	654	677	0.828836324	8.77E-05	1.78
HIST1H2BD	NM_021063	6673	6072	6892	8357	5627	7264	0.830442618	6.84E-07	1.78
NDUFS5	NM_001184979	844	671	622	945	464	1076	0.830986458	0.00031	1.78
COMMD8	NM_017845	90	68	102	137	53	124	0.831259345	0.00208	1.78
PRDX4	NM_006406	88	67	67	128	55	88	0.833063423	0.00218	1.78
NDUFV1	NM_007103	2142	1865	2419	2807	1892	2265	0.835362404	9.08E-07	1.78
PSMC1	NM_002802	969	972	1434	1486	1088	1090	0.835735318	4.27E-05	1.78
TEX264	NM_001129884	132	113	101	191	96	123	0.836118153	0.00057	1.79
LAMTOR2	NM_014017	364	350	378	475	346	383	0.836727683	3.80E-05	1.79
ATP5G1	NM_001002027	297	374	408	556	276	397	0.837976976	6.80E-06	1.79
RPS12	NM_001016	7912	6938	8511	10403	6657	8498	0.845342868	8.76E-08	1.80
LGALS1	NM_002305	7757	5374	6310	9631	6338	5790	0.845364453	3.97E-05	1.80
HIST1H1A	NM_005325	447	353	255	688	251	381	0.852054834	0.00043	1.81
CEBPZ	NM_005760	138	140	91	209	71	197	0.856100911	0.00268	1.81
PCOLCE	NM_002593	384	431	347	561	357	417	0.857100607	3.64E-05	1.81
AUH	NM_001698	45	40	47	73	39	55	0.860307497	0.00457	1.82
IMP4	NM_033416	554	501	558	727	510	567	0.861021327	5.86E-06	1.82
GEM	NM_005261	345	370	449	536	315	468	0.861099345	2.53E-06	1.82
HIST1H4F	NM_003540	1743	1324	1374	2546	916	1914	0.865574033	8.64E-06	1.82
POLR2L	NM_021128	2731	2277	2538	3538	2324	2600	0.867633832	6.26E-07	1.82
TBRG4	NM_004749	532	419	407	641	406	519	0.872348822	1.34E-05	1.83

C3orf52	NM_024616	36	16	36	51	26	44	0.873543724	0.0125	1.83
PSMD4	NM_002810	42	23	31	34	67	29	0.877095978	0.02253	1.84
HIST1H2BE	NM_003523	1210	848	1004	1584	750	1279	0.885275068	6.54E-07	1.85
TRPV2	NM_016113	83	85	78	148	53	115	0.886077604	0.00078	1.85
COX7B	NM_001866	2462	2019	2343	3215	1631	3123	0.886358846	4.04E-07	1.85
AP2S1	NM_004069	1261	1064	1356	1987	865	1471	0.887320208	3.30E-09	1.85
NDUFA1	NM_004541	1205	976	1072	1805	846	1185	0.889709316	1.72E-08	1.85
SPHK1	NM_182965	829	700	885	1052	776	902	0.89051798	2.15E-06	1.85
CCT3	NM_005998	1651	2053	1616	2721	1424	2213	0.897654326	1.17E-06	1.86
HIST1H4B	NM_003544	2112	1736	1745	2756	1431	2419	0.897803276	2.52E-07	1.86
HIST3H2BB	NM_175055	632	707	676	975	600	771	0.898665456	3.79E-07	1.86
MYDGF	NM_019107	343	299	400	436	332	432	0.902947462	1.31E-05	1.87
IFI27L2	NM_032036	56	45	95	66	90	87	0.907095874	0.00635	1.88
ARMC10	NM_001161009	104	76	94	150	88	101	0.907592323	0.00028	1.88
RNF121	NM_018320	267	195	270	384	270	217	0.908033029	0.00011	1.88
NDUFS6	NM_004553	532	395	413	691	395	513	0.910341982	1.87E-06	1.88
METTL1	NM_005371	91	90	98	186	73	101	0.916284385	0.00018	1.89
EIF4E2	NM_004846	149	105	117	215	88	172	0.923109028	0.00011	1.90
UQCC3	NM_001085372	207	202	253	375	167	269	0.923255909	8.21E-07	1.90
ANO3	NM_031418	17	8	10	30	15	18	0.925132836	0.01791	1.90
KRTAP2-3	NM_001165252	11	9	20	32	16	20	0.925886516	0.01628	1.90
ATP5G3	NM_001002258	1530	1156	1467	2089	1254	1545	0.926903335	1.96E-08	1.90
C12orf73	NM_001135570	29	31	27	51	34	38	0.931108262	0.00544	1.91
UTP18	NM_016001	235	218	247	384	173	307	0.931549035	8.21E-07	1.91
HIST1H2BI	NM_003525	2080	1622	1767	3054	1324	2348	0.932283848	1.96E-08	1.91
MTFP1	NM_016498	189	118	151	240	147	179	0.932594882	5.62E-05	1.91
PARK7	NM_007262	3058	2492	2624	3841	2668	3120	0.933700321	3.08E-07	1.91
MCTS1	NM_014060	17	10	13	27	12	32	0.947140725	0.01379	1.93
PDIA6	NM_005742	2194	2184	2240	3207	1825	2953	0.949416632	4.64E-09	1.93
WDR46	NM_005452	141	122	190	151	155	257	0.950743281	0.00074	1.93
SYTL3	NM_001242384	142	122	109	265	95	142	0.953084363	6.94E-05	1.94
HIST1H4E	NM_003545	2510	1737	2150	3628	1849	2375	0.953907633	9.98E-09	1.94
LSM7	NM_016199	750	529	611	953	524	848	0.953959751	3.30E-07	1.94
IL24	NM_001185156	17	32	30	57	18	51	0.958041274	0.00653	1.94
TMEM208	NM_014187	145	125	131	239	106	176	0.969062364	6.77E-06	1.96
ATP5I	NM_007100	426	225	242	473	276	411	0.971822471	6.89E-05	1.96
EED	NM_003797	155	113	150	203	108	233	0.975965773	5.92E-05	1.97
CCDC47	NM_020198	488	363	312	578	348	586	0.99345379	9.71E-06	1.99
NDUFA8	NM_014222	404	399	432	642	349	566	0.994316681	9.17E-09	1.99
SLC50A1	NM_018845	176	196	234	306	202	259	0.99955655	1.04E-06	2.00
PSMC3	NM_002804	2092	1625	2286	2628	2296	2377	1.001605124	1.33E-06	2.00
RRP15	NM_016052	114	90	105	174	96	139	1.004497455	1.19E-05	2.01
DYNLT1	NM_006519	420	318	401	595	319	554	1.016280392	4.37E-08	2.02
HIST1H4H	NM_003543	5539	5078	3899	8708	3478	7757	1.021391999	1.01E-06	2.03
MMP3	NM_002422	43	42	42	61	50	72	1.028198627	0.00074	2.04
TMEM203	NM_053045	49	51	38	89	52	64	1.04749031	0.00032	2.07

CYC1	NM_001916	548	407	528	900	500	603	1.092430801	1.23E-10	2.13
POLR3K	NM_016310	273	247	279	435	271	376	1.096122651	2.86E-09	2.14
NOSIP	NM_015953	310	191	301	396	323	373	1.106828322	9.71E-07	2.15
SLCO4A1	NM_016354	42	28	29	57	42	63	1.124202852	0.00035	2.18
CARNMT1	NM_152420	24	23	23	47	31	45	1.149888996	0.00043	2.22
HIST1H4A	NM_003538	1941	1582	1666	2885	2214	2146	1.158358159	3.30E-09	2.23
TSEN54	NM_207346	35	34	47	82	54	59	1.211235891	2.38E-05	2.32
NDUFA2	NM_002488	399	332	210	676	359	487	1.224791363	1.11E-07	2.34
ZBED2	NM_024508	55	65	43	191	61	126	1.541948147	2.84E-09	2.91

Table S5. Gene Ontology terms overrepresented among the differentiating genes (q.value <0.05) in PROX1 depleted cells. ID–GO identifier, GeneRatio – the ratio of number of differentiating genes in a given term to the number of differentiating genes with GO identifier ; BgRatio—the ratio of number of not differentiating genes in a given term to the number of expressed genes with GO identifier; p-value-p-value in hypergeometric test; q-value-p-value after FDR correction; Count—number of differentiating genes contributing to a given term; BP—biological processes BP, MF—molecular function; CC—cellular component.

**Biological Process - upregulated**							
**ID**	**Description**	**GeneRatio**	**BgRatio**	**pvalue**	**p.adjust**	**qvalue**	**Count**
GO:0022613	ribonucleoprotein complex biogenesis	98/675	410/8624	4.2E-25	1.5E-21	1.37E-21	98
GO:0006413	translational initiation	54/675	166/8624	1.17E-20	1.73E-17	1.58E-17	54
GO:0006364	rRNA processing	64/675	230/8624	3.07E-20	2.74E-17	2.5E-17	64
GO:0006614	SRP-dependent cotranslational protein targeting to membrane	35/675	82/8624	4.26E-18	2.53E-15	2.31E-15	35
GO:0019080	viral gene expression	48/675	160/8624	7.23E-17	2.86E-14	2.61E-14	48
GO:0072599	establishment of protein localization to endoplasmic reticulum	35/675	90/8624	1.48E-16	5.29E-14	4.83E-14	35
GO:0000184	nuclear-transcribed mRNA catabolic process, nonsense-mediated decay	37/675	107/8624	1.76E-15	5.21E-13	4.76E-13	37
GO:0019083	viral transcription	44/675	149/8624	2.82E-15	7.72E-13	7.05E-13	44
GO:0044033	multi-organism metabolic process	48/675	177/8624	5.96E-15	1.42E-12	1.29E-12	48
GO:0022618	ribonucleoprotein complex assembly	43/675	176/8624	7.68E-12	1.3E-09	1.19E-09	43
GO:1901361	organic cyclic compound catabolic process	58/675	296/8624	3.22E-11	4.99E-09	4.56E-09	58
GO:0071826	ribonucleoprotein complex subunit organization	43/675	185/8624	4.48E-11	6.65E-09	6.07E-09	43
GO:0019439	aromatic compound catabolic process	57/675	292/8624	5.63E-11	8.03E-09	7.33E-09	57
GO:0006323	DNA packaging	36/675	139/8624	6.84E-11	9.38E-09	8.56E-09	36
GO:0046700	heterocycle catabolic process	56/675	289/8624	1.13E-10	1.49E-08	1.36E-08	56
GO:0044270	cellular nitrogen compound catabolic process	56/675	292/8624	1.71E-10	2.18E-08	1.99E-08	56
GO:0051290	protein heterotetramerization	15/675	28/8624	3.12E-10	3.7E-08	3.38E-08	15
GO:0006334	nucleosome assembly	29/675	101/8624	3.55E-10	4.09E-08	3.73E-08	29
GO:0034728	nucleosome organization	32/675	122/8624	5.62E-10	6.26E-08	5.71E-08	32

GO:0065004	protein-DNA complex assembly	38/675	165/8624	8.23E-10	8.62E-08	7.87E-08	38
GO:0019058	viral life cycle	59/675	329/8624	8.61E-10	8.76E-08	8E-08	59
GO:0006119	oxidative phosphorylation	25/675	83/8624	1.97E-09	1.85E-07	1.69E-07	25
GO:0045815	positive regulation of gene expression, epigenetic	20/675	63/8624	3.06E-08	2.47E-06	2.26E-06	20
GO:0006342	chromatin silencing	22/675	76/8624	4.14E-08	3.14E-06	2.86E-06	22
GO:0022900	electron transport chain	22/675	81/8624	1.45E-07	9.97E-06	9.1E-06	22
GO:0022904	respiratory electron transport chain	22/675	81/8624	1.45E-07	9.97E-06	9.1E-06	22
GO:0009126	purine nucleoside monophosphate metabolic process	38/675	198/8624	1.59E-07	1.05E-05	9.58E-06	38
GO:0009167	purine ribonucleoside monophosphate metabolic process	38/675	198/8624	1.59E-07	1.05E-05	9.58E-06	38
GO:1904872	regulation of telomerase RNA localization to Cajal body	9/675	15/8624	3.41E-07	2.06E-05	1.88E-05	9
GO:1904874	positive regulation of telomerase RNA localization to Cajal body	9/675	15/8624	3.41E-07	2.06E-05	1.88E-05	9
GO:0031145	anaphase-promoting complex-dependent catabolic process	19/675	70/8624	1.04E-06	5.25E-05	4.79E-05	19
GO:0097031	mitochondrial respiratory chain complex I biogenesis	16/675	52/8624	1.19E-06	5.37E-05	4.91E-05	16
GO:0045653	negative regulation of megakaryocyte differentiation	7/675	11/8624	4.36E-06	0.000183	0.000167	7
GO:0006457	protein folding	30/675	164/8624	8.89E-06	0.000333	0.000304	30
GO:0033238	regulation of cellular amine metabolic process	14/675	49/8624	1.45E-05	0.000506	0.000462	14
GO:0051444	negative regulation of ubiquitin-protein transferase activity	17/675	70/8624	1.95E-05	0.000648	0.000592	17
GO:1904668	positive regulation of ubiquitin protein ligase activity	17/675	70/8624	1.95E-05	0.000648	0.000592	17
GO:0006091	generation of precursor metabolites and energy	38/675	240/8624	2.02E-05	0.000653	0.000596	38
GO:1901657	glycosyl compound metabolic process	42/675	278/8624	2.39E-05	0.000761	0.000695	42
GO:0000387	spliceosomal snRNP assembly	11/675	35/8624	4.54E-05	0.001363	0.001244	11
GO:0030162	regulation of proteolysis	53/675	394/8624	5.9E-05	0.001722	0.001572	53
GO:0002220	innate immune response activating cell surface receptor signaling pathway	17/675	78/8624	8.56E-05	0.002365	0.002159	17
GO:0002223	stimulatory C-type lectin receptor signaling pathway	17/675	78/8624	8.56E-05	0.002365	0.002159	17
GO:0042176	regulation of protein catabolic process	40/675	275/8624	8.89E-05	0.002435	0.002223	40
GO:0002479	antigen processing and presentation of exogenous	12/675	44/8624	9.77E-05	0.002618	0.00239	12

	peptide antigen via MHC class I, TAP-dependent						
GO:0042590	antigen processing and presentation of exogenous peptide antigen via MHC class I	12/675	44/8624	9.77E-05	0.002618	0.00239	12
GO:0022411	cellular component disassembly	50/675	380/8624	0.000167	0.004153	0.003791	50
GO:0042776	mitochondrial ATP synthesis coupled proton transport	7/675	17/8624	0.000169	0.004183	0.003819	7
GO:0006289	nucleotide-excision repair	19/675	98/8624	0.000177	0.004317	0.003941	19
GO:0038061	NIK/NF-kappaB signaling	17/675	84/8624	0.000224	0.00526	0.004801	17
GO:0009894	regulation of catabolic process	47/675	355/8624	0.000226	0.00526	0.004801	47
GO:0030219	megakaryocyte differentiation	9/675	29/8624	0.000251	0.005658	0.005165	9
GO:0060071	Wnt signaling pathway, planar cell polarity pathway	17/675	86/8624	0.000301	0.006742	0.006155	17
GO:0007339	binding of sperm to zona pellucida	6/675	14/8624	0.000391	0.008393	0.007662	6
GO:0035036	sperm-egg recognition	6/675	14/8624	0.000391	0.008393	0.007662	6
GO:0031349	positive regulation of defense response	30/675	202/8624	0.000464	0.009724	0.008877	30
GO:0045727	positive regulation of translation	14/675	66/8624	0.000477	0.009889	0.009027	14
GO:0070203	regulation of establishment of protein localization to telomere	5/675	10/8624	0.000523	0.010702	0.00977	5
GO:0015985	energy coupled proton transport, down electrochemical gradient	7/675	20/8624	0.000547	0.010824	0.009882	7
GO:0019693	ribose phosphate metabolic process	40/675	307/8624	0.000899	0.016513	0.015075	40
GO:0001736	establishment of planar polarity	17/675	95/8624	0.000999	0.017971	0.016405	17
GO:0007164	establishment of tissue polarity	17/675	95/8624	0.000999	0.017971	0.016405	17
GO:0051897	positive regulation of protein kinase B signaling	10/675	42/8624	0.001189	0.020877	0.019058	10
GO:0033209	tumor necrosis factor-mediated signaling pathway	16/675	91/8624	0.001686	0.027943	0.025508	16
GO:0051169	nuclear transport	41/675	334/8624	0.002463	0.03883	0.035448	41
GO:0009988	cell-cell recognition	6/675	19/8624	0.002517	0.038994	0.035597	6
GO:0060765	regulation of androgen receptor signaling pathway	6/675	19/8624	0.002517	0.038994	0.035597	6
GO:0032481	positive regulation of type I interferon production	11/675	54/8624	0.002657	0.040625	0.037086	11
GO:0031329	regulation of cellular catabolic process	34/675	267/8624	0.003113	0.046208	0.042183	34
GO:0006940	regulation of smooth muscle contraction	6/675	20/8624	0.003361	0.048685	0.044444	6
GO:1905330	regulation of morphogenesis of an epithelium	18/675	115/8624	0.003424	0.049388	0.045086	18
**Molecular Function - upregulated**							
GO:0003735	structural constituent of ribosome	53/661	169/8488	1.37E-19	8.22E-17	7.55E-17	53

GO:0019843	rRNA binding	18/661	56/8488	1.12E-07	2.24E-05	2.06E-05	18
GO:0046982	protein heterodimerization activity	42/661	257/8488	2.84E-06	0.000425	0.000391	42
GO:0051082	unfolded protein binding	19/661	79/8488	6.88E-06	0.000687	0.000631	19
GO:0003954	NADH dehydrogenase activity	11/661	35/8488	4.33E-05	0.002838	0.002608	11
GO:0008137	NADH dehydrogenase (ubiquinone) activity	11/661	35/8488	4.33E-05	0.002838	0.002608	11
GO:0050136	NADH dehydrogenase (quinone) activity	11/661	35/8488	4.33E-05	0.002838	0.002608	11
GO:0043021	ribonucleoprotein complex binding	19/661	93/8488	7.94E-05	0.004322	0.003972	19
GO:0045296	cadherin binding	36/661	260/8488	0.000473	0.018892	0.017363	36
GO:0098631	protein binding involved in cell adhesion	36/661	260/8488	0.000473	0.018892	0.017363	36
GO:0070628	proteasome binding	5/661	10/8488	0.000511	0.019122	0.017575	5
GO:0015078	hydrogen ion transmembrane transporter activity	13/661	60/8488	0.000579	0.019559	0.017976	13
GO:0008026	ATP-dependent helicase activity	16/661	84/8488	0.000653	0.019559	0.017976	16
GO:0070035	purine NTP-dependent helicase activity	16/661	84/8488	0.000653	0.019559	0.017976	16
GO:0001054	RNA polymerase I activity	5/661	12/8488	0.001407	0.035474	0.032603	5
GO:0016779	nucleotidyltransferase activity	18/661	107/8488	0.001421	0.035474	0.032603	18
GO:0046933	proton-transporting ATP synthase activity, rotational mechanism	5/661	13/8488	0.002141	0.047503	0.043659	5
**Cellular Component - upregulated**							
GO:0005840	ribosome	60/693	189/9021	1.27E-22	6.16E-20	5.18E-20	60
GO:0044445	cytosolic part	54/693	166/9021	5.19E-21	1.26E-18	1.06E-18	54
GO:0044815	DNA packaging complex	33/693	67/9021	9.35E-20	1.13E-17	9.52E-18	33
GO:0000786	nucleosome	31/693	61/9021	3.79E-19	3.06E-17	2.57E-17	31
GO:0005743	mitochondrial inner membrane	76/693	363/9021	1.85E-16	1.28E-14	1.08E-14	76
GO:0032993	protein-DNA complex	38/693	117/9021	4.25E-15	2.58E-13	2.16E-13	38
GO:0070469	respiratory chain	23/693	67/9021	3.38E-10	1.17E-08	9.84E-09	23
GO:0098803	respiratory chain complex	19/693	56/9021	1.48E-08	3.98E-07	3.34E-07	19
GO:0005852	eukaryotic translation initiation factor 3 complex	8/693	15/9021	4.61E-06	7.46E-05	6.26E-05	8
GO:0031012	extracellular matrix	35/693	224/9021	3.89E-05	0.000553	0.000464	35
GO:1905369	endopeptidase complex	14/693	59/9021	0.000112	0.00133	0.001117	14
GO:1905368	peptidase complex	16/693	75/9021	0.000145	0.001597	0.001341	16
GO:0005682	U5 snRNP	6/693	14/9021	0.000353	0.003296	0.002768	6
GO:0005913	cell-cell adherens junction	35/693	270/9021	0.001478	0.012355	0.010378	35
GO:0070069	cytochrome complex	5/693	16/9021	0.005638	0.039063	0.032811	5
**Biological Process - downregulated**							
GO:0051270	regulation of cellular component movement	51/422	405/8624	2.79E-10	5.27E-07	4.54E-07	51
GO:0040012	regulation of locomotion	50/422	395/8624	3.53E-10	5.27E-07	4.54E-07	50
GO:2000145	regulation of cell motility	49/422	387/8624	5.35E-10	5.27E-07	4.54E-07	49

GO:0031175	neuron projection development	53/422	440/8624	6.26E-10	5.27E-07	4.54E-07	53
GO:0060284	regulation of cell development	48/422	418/8624	2.08E-08	9.99E-06	8.6E-06	48
GO:0048589	developmental growth	37/422	309/8624	3.4E-07	8.79E-05	7.56E-05	37
GO:0030198	extracellular matrix organization	24/422	155/8624	4.42E-07	9.28E-05	7.99E-05	24
GO:0043062	extracellular structure organization	24/422	155/8624	4.42E-07	9.28E-05	7.99E-05	24
GO:0008285	negative regulation of cell proliferation	40/422	354/8624	5.23E-07	0.000104	8.92E-05	40
GO:0051493	regulation of cytoskeleton organization	30/422	249/8624	4.03E-06	0.000589	0.000507	30
GO:0051384	response to glucocorticoid	13/422	63/8624	8.55E-06	0.00115	0.00099	13
GO:0009743	response to carbohydrate	18/422	115/8624	1.06E-05	0.001319	0.001136	18
GO:0014065	phosphatidylinositol 3-kinase signaling	13/422	73/8624	4.51E-05	0.003608	0.003107	13
GO:0032970	regulation of actin filament-based process	21/422	170/8624	7.97E-05	0.005253	0.004523	21
GO:0032535	regulation of cellular component size	22/422	191/8624	0.000154	0.00823	0.007087	22
GO:0030278	regulation of ossification	14/422	95/8624	0.000196	0.009986	0.008599	14
GO:0010810	regulation of cell-substrate adhesion	15/422	107/8624	0.000207	0.010218	0.008798	15
GO:0010463	mesenchymal cell proliferation	7/422	27/8624	0.000242	0.01129	0.009722	7
GO:0071385	cellular response to glucocorticoid stimulus	7/422	27/8624	0.000242	0.01129	0.009722	7
GO:0050673	epithelial cell proliferation	21/422	185/8624	0.000266	0.012075	0.010397	21
GO:0006935	chemotaxis	23/422	213/8624	0.000288	0.012915	0.01112	23
GO:0090066	regulation of anatomical structure size	24/422	229/8624	0.000335	0.014434	0.012429	24
GO:0043068	positive regulation of programmed cell death	32/422	346/8624	0.000368	0.015085	0.012989	32
GO:0050900	leukocyte migration	16/422	125/8624	0.000372	0.015085	0.012989	16
GO:0015804	neutral amino acid transport	5/422	14/8624	0.00038	0.015085	0.012989	5
GO:1905276	regulation of epithelial tube formation	5/422	14/8624	0.00038	0.015085	0.012989	5
GO:0010631	epithelial cell migration	17/422	138/8624	0.000389	0.015085	0.012989	17
GO:0090132	epithelium migration	17/422	138/8624	0.000389	0.015085	0.012989	17
GO:0032271	regulation of protein polymerization	14/422	102/8624	0.000417	0.015941	0.013727	14
GO:0007610	behavior	24/422	233/8624	0.000433	0.016163	0.013918	24
GO:1903825	organic acid transmembrane transport	7/422	31/8624	0.000604	0.020519	0.017668	7
GO:1905039	carboxylic acid transmembrane transport	7/422	31/8624	0.000604	0.020519	0.017668	7
GO:0071417	cellular response to organonitrogen compound	26/422	268/8624	0.000637	0.02143	0.018453	26
GO:0031589	cell-substrate adhesion	20/422	184/8624	0.000651	0.02143	0.018453	20
GO:0010001	glial cell differentiation	13/422	96/8624	0.000758	0.023592	0.020315	13
GO:1901699	cellular response to nitrogen compound	29/422	316/8624	0.000782	0.02391	0.020588	29

GO:0061448	connective tissue development	15/422	121/8624	0.000791	0.023964	0.020635	15
GO:0061387	regulation of extent of cell growth	9/422	53/8624	0.00096	0.027585	0.023753	9
GO:1901698	response to nitrogen compound	38/422	461/8624	0.001023	0.028663	0.024681	38
GO:0044087	regulation of cellular component biogenesis	38/422	462/8624	0.001065	0.029593	0.025482	38
GO:0090288	negative regulation of cellular response to growth factor stimulus	11/422	76/8624	0.00109	0.03005	0.025876	11
GO:0014070	response to organic cyclic compound	38/422	464/8624	0.001153	0.031027	0.026716	38
GO:0070373	negative regulation of ERK1 and ERK2 cascade	7/422	35/8624	0.001304	0.034266	0.029505	7
GO:1901655	cellular response to ketone	8/422	45/8624	0.001343	0.035	0.030138	8
GO:0098742	cell-cell adhesion via plasma-membrane adhesion molecules	10/422	67/8624	0.001432	0.036478	0.03141	10
GO:0051093	negative regulation of developmental process	33/422	393/8624	0.001607	0.04043	0.034813	33
GO:0043269	regulation of ion transport	19/422	184/8624	0.001628	0.04043	0.034813	19
GO:0009611	response to wounding	28/422	316/8624	0.001635	0.04043	0.034813	28
GO:0030203	glycosaminoglycan metabolic process	11/422	81/8624	0.001854	0.043291	0.037276	11
GO:0090342	regulation of cell aging	6/422	28/8624	0.00199	0.04584	0.039471	6
GO:0010563	negative regulation of phosphorus metabolic process	27/422	306/8624	0.002092	0.046598	0.040124	27
GO:0045936	negative regulation of phosphate metabolic process	27/422	306/8624	0.002092	0.046598	0.040124	27
GO:0003013	circulatory system process	18/422	178/8624	0.002694	0.053748	0.046281	18
GO:1902043	positive regulation of extrinsic apoptotic signaling pathway via death domain receptors	4/422	13/8624	0.002839	0.055729	0.047986	4
GO:0198738	cell-cell signaling by wnt	27/422	314/8624	0.003013	0.057246	0.049293	27
**Molecular Function - downregulated**							
GO:0035591	signaling adaptor activity	9/413	36/8488	4.08E-05	0.014981	0.013383	9
GO:0005070	SH3/SH2 adaptor activity	7/413	23/8488	7.63E-05	0.014981	0.013383	7
GO:0017022	myosin binding	8/413	31/8488	8.61E-05	0.014981	0.013383	8
GO:0019838	growth factor binding	12/413	72/8488	0.000162	0.016891	0.015089	12
GO:0004871	signal transducer activity	38/413	437/8488	0.000326	0.028337	0.025314	38
GO:0015175	neutral amino acid transmembrane transporter activity	5/413	15/8488	0.000533	0.039726	0.035488	5
GO:0008201	heparin binding	9/413	52/8488	0.000798	0.047426	0.042367	9
GO:0000982	transcription factor activity, RNA polymerase II core promoter proximal region sequence-specific binding	20/413	189/8488	0.00085	0.047426	0.042367	20
GO:0001077	transcriptional activator activity, RNA polymerase II core promoter proximal	15/413	124/8488	0.000963	0.047426	0.042367	15

	region sequence-specific binding						
GO:0001228	transcriptional activator activity, RNA polymerase II transcription regulatory region sequence-specific binding	18/413	164/8488	0.000999	0.047426	0.042367	18
GO:0019904	protein domain specific binding	33/413	387/8488	0.001126	0.048963	0.04374	33
GO:0016684	oxidoreductase activity, acting on peroxide as acceptor	6/413	26/8488	0.001283	0.050206	0.044851	6
GO:0016303	1-phosphatidylinositol-3-kinase activity	5/413	18/8488	0.001347	0.050206	0.044851	5
GO:0005539	glycosaminoglycan binding	10/413	68/8488	0.001539	0.053564	0.04785	10
**Cellular Component - downregulated**							
GO:0005578	proteinaceous extracellular matrix	22/439	115/9021	2.62E-08	9.82E-06	9.17E-06	22
GO:0044420	extracellular matrix component	14/439	57/9021	3.82E-07	7.15E-05	6.69E-05	14
GO:0005615	extracellular space	34/439	393/9021	0.000752	0.049225	0.046013	34
GO:0043235	receptor complex	15/439	122/9021	0.000819	0.049225	0.046013	15
GO:0098644	complex of collagen trimers	4/439	10/9021	0.000919	0.049225	0.046013	4
